# Synthesis and Chiroptical Properties of Bithiophene-Functionalized Open and Methylene-Bridged Binaphthyl Derivatives

**DOI:** 10.1021/acs.joc.5c01954

**Published:** 2025-10-02

**Authors:** Federico Dini, Giorgia Puntoni, Lilia Tinagli, Lorenzo Di Bari, Gennaro Pescitelli, Gianluigi Albano

**Affiliations:** † Dipartimento di Chimica e Chimica Industriale, 9310 Università di Pisa, Via Giuseppe Moruzzi 13, 56124 Pisa, Italy; ‡ Dipartimento di Scienze Chimiche, della Vita e della Sostenibilità Ambientale, Università degli Studi di Parma, Parco Area delle Scienze 17/A, 43124 Parma, Italy

## Abstract

Thiophene-based π-conjugated compounds are one of the most important classes of organic materials for optoelectronic applications. In this context, herein we report the synthesis of two enantiopure binaphthyl systems: an open and a methylene-bridged 1,1′-bi-2-naphthol (BINOL) derivative, functionalized at the C6/C6′ positions with lateral 2,2′-bithiophen-5-yl moieties (compounds (a*R*)-**3** and (a*R*)-**6**, respectively). Both final products were subjected, together with their synthetic precursors (i.e., the corresponding unsubstituted and 6,6′-dibrominated BINOL derivatives), to UV–vis absorption and electronic circular dichroism (ECD) investigation in solution and thin films. A computational analysis based on density functional theory (DFT) and time-dependent DFT (TD-DFT) allowed us to gain physical insight into the observed (chiro)­optical properties, emphasizing the crucial role of thiophene rings as C6/C6′ substituents. Moreover, the occurrence of induced ECD and circularly polarized luminescence (CPL) signals was observed in preliminary tests of chiroptical induction, performed for thin films of achiral benzothiadiazole (BTD) dye **7** in the presence of small percentages (0.5–5.0 mol %) of the methylene-bridged target molecule (a*R*)-**6** as chiral inducer.

## Graphical abstract

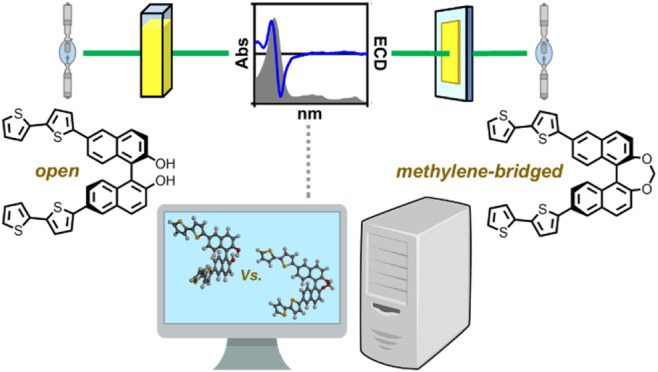

## 1 Introduction

In the last decades, chiral organic π-conjugated materials have undergone an extraordinary development as semiconducting active layers for a range of cutting-edge technological applications, thanks to their unique functional properties.^1–4^ One of them is the so-called “chirality-induced spin selectivity” (CISS) effect,^5–7^ successfully applied in spintronics^8–11^ and spin-controlled electrochemical reactions, such as oxygen evolution reaction or oxygen reduction reactions.^12^ Besides their peculiar electrochemical^13,14^ and magneto-chiral features,^15–17^ the most important are definitely their chiroptical properties, i.e., the result of differential absorption, emission, and/or depolarization of circularly polarized (CP) light with opposite handedness, which allows for the design of devices^18^ such as CP organic light-emitting diodes,^19–21^ organic field-effect transistors,^22^ and organic photodetectors.^23,24^

Due to the occurrence of linear anisotropies, the study of chiroptical properties in absorption (electronic circular dichroism, ECD) and in emission (circularly polarized luminescence, CPL) in thin films of chiral organic π-conjugated materials is more challenging than that in solution. As described by the Mueller matrix model,^25,26^ for solid-state samples, the experimental ECD and CPL signals can be expressed as the sum of several contributions, two of which are the most relevant for thin films: an *intrinsic* (or reciprocal) and a *nonreciprocal* (or apparent) component,^27^ each of them associated with a different structural chirality.^28^ The intrinsic term is invariant upon thin film orientation and proportional to the rotational strength of electronic transitions (which is the only responsible for ECD and CPL in isotropic media).^27,28^ It is the expression of a three-dimensional (3D) chirality, at the molecular or supramolecular level (nanoscopic scale, 1–10 nm and above).^29^ The nonreciprocal term is invariant upon sample rotation around the instrumental optical axis but inverts its sign upon sample flipping with respect to the optical axis. It arises from a skewed coupling between linear anisotropies: linear dichroism (LD) and linear birefringence (LB), in the case of nonreciprocal ECD; linear fluorescence anisotropy (*f*) and linear birefringence (LB), in the case of nonreciprocal CPL.^27,28^ The occurrence of nonparallel and nonorthogonal linear components is related to the lack of a mirror plane parallel to the optical axis. For this reason, it can be considered an expression of two-dimensional (2D) chirality.^29^ In the specific case of ECD measurements, it is possible to isolate the intrinsic and nonreciprocal components (also known as CDiso and LDLB, respectively) from the measured chiroptical spectra by taking, respectively, the semisum and the semidifference of the spectra recorded for the front face (organic layer toward the light source) and the back side (organic layer toward the detector).^28^ Furthermore, it is possible to quantitatively evaluate their relative weights by calculating the ratio ρ between the integrated areas of absolute values of semidifference (∫|LDLB|) and semisum (∫|CDiso|).

Embedding chirality in π-conjugated materials can be reached through several different approaches. A very common one consists in decorating the π-conjugated skeleton with enantiopure appendages. The possibility of modifying only the chiral side chain, while keeping the same chromophore, allows for modulating the aggregate features, while maintaining the gross of the spectroscopic (nonchiroptical) features of the chromophore. In such a way, one can easily predict where significant absorption bands will fall and where the ECD signals will be expected. In principle, it is also possible to predict the spectral region for emission spectroscopy, although aggregation-caused quenching may frequently lead to the collapse of luminescence. Although in a poorly predictable manner, the side chains structure greatly contributes in determining physicochemical properties of the aggregate, notably stability and solubility, which is key to improve processability in device fabrication.^30–32^ A different approach involves the use of compounds where chirality is incorporated directly into the π-conjugated backbone.^33^ Among them, 1,1′-binaphthyl systems exhibiting axial chirality are definitely the most investigated.^34^ The presence of substituents at the C2/C2′ positions is a requirement for achieving enantiopure 1,1′-binaphthyl compounds with a kinetically stable chiral configuration and avoiding racemization.^34^ Typical examples are 1,1′-bi-2-naphthol (BINOL) and its derivatives, obtained by etherification of the two hydroxyl groups with alkyl or aryl moieties.^35^

The molecular conformation of 2,2′-substituted-1,1′-binaphthyl compounds is strongly dependent on the nature of the substituents at C2/C2′ positions: when these are either small or capable of intramolecular hydrogen bonding, a *cisoid* conformation is preferred; when they are sterically hindered, a *transoid* conformation may be preferred.^34,36^ More specifically, most BINOL derivatives in solution show a *quasi*-orthogonal arrangement of the two naphthyl moieties,^37,38^ associated with an almost barrierless librational motion over a 90° ± 25° range. Therefore, small variations in the dihedral angle θ between the two naphthyl planes, depending on the solvent and/or other experimental conditions, could result in either cisoid or transoid conformation, even for the same enantiopure compound.^39–41^ A possible way for anchoring the dihedral angle of a BINOL, and in turn its molecular conformation, is to connect the two oxygen atoms through an alkyl chain bridge.^42^ In particular, methylene-bridged BINOL derivatives exhibit a highly rigid cisoid conformation with smaller dihedral angles compared with the corresponding open systems.

ECD spectra of 2,2′-substituted-1,1′-binaphthyl derivatives immediately reveal their absolute configuration and deliver quick information about the dihedral angle, as its value largely impacts both the signal intensity and the wavelength splitting of the excitonic couplet.^43–46^ It is not surprising that, for a long time, chiroptical investigations of both open and bridged BINOL derivatives have been limited to ECD spectra in solution for conformational analysis.^47^

After a few pioneering studies on enantiopure BINOL, either as monolayers at the air/quartz surface^48–50^ or as Langmuir–Blodgett–Schaefer thin films,^51^ reported in the late 1990s and early 2000s, only in the last 15 years the chiroptical investigation of BINOL derivatives in thin films has gained interest. Guy et al. investigated the pulsed laser deposition (PLD) technique to obtain highly isotropic thin films of methylene-bridged BINOL^52^ and other BINOLs,^53^ studying the impact of the laser fluence on the ECD signals, showing moderate to pronounced deviations from the corresponding solution spectra. Imai and Fujiki’s groups made an important contribution, studying ECD and CPL of many open and bridged BINOLs, involving different functionalization of hydroxy groups and different substituents at C3/C3′ positions, although only as blends in poly­(methyl methacrylate) (PMMA) thin films.^54–60^ Recently, Kartouzian et al. investigated thin films of enantiopure (a*R*)-BINOL prepared by ultrahigh-vacuum evaporation, which showed a progressive evolution of the chiroptical signals upon simple aging, from a pure intrinsic ECD spectrum (for freshly prepared samples) to an almost pure nonreciprocal contribution (after 3 days).^61,62^ Surprisingly, a systematic chiroptical study of thin films of simple open and methylene-bridged BINOLs, prepared as neat materials by spin coating or drop casting (which are the most widely used solution-processable techniques in organic optoelectronics), has not been reported so far.

Another interesting aspect is that enantiopure BINOL derivatives have been used as chirality inducers. Kartouzian et al. reported the occurrence of induced ECD signals for spin-coated thin films of achiral rhodamine dye in the presence of BINOL as a chiral modifier, differing strongly and nonlinearly in strength by changing the angular speed of the spin coating process or BINOL relative concentration.^63^ Other open BINOLs have been successfully employed for the same purpose, generating strong induced ECD and CPL signals in thin films of achiral or racemic π-conjugated polymers: polyacetylenes,^64^ poly­(9,9-dioctylfluorene-*co*-benzothiadiazole) (F8BT),^65^ and polythiophenes.^66,67^ In the past few years, there has been growing interest in the use of methylene-bridged BINOLs as chiroptical inducers, as the highly rigid cisoid conformation due to the anchored dihedral angle can greatly improve their ability to cause chiral induction. The impact of various substituents at the C3/C3′ positions of the methylene-bridged system was evaluated, observing the occurrence of remarkable induced ECD and CPL signals for thin films of achiral π-conjugated small molecules^68^ and polymers.^69–72^ However, there is still plenty of room for improvement, especially in the chiroptical induction of achiral low molecular weight π-conjugated dyes.

Thiophene-based π-conjugated compounds represent one of the most important classes of organic materials for optoelectronic and nanotechnology applications.^73–75^ For this reason, over the past decade, we contributed ourselves to this field with the development of several families of chiral thiophene-based organic π-conjugated dyes, consisting of an (hetero)­aromatic central core—hydroquinone (HQ), 1,5-dihydroxynaphthalene (Naph), benzo­[1,2-*b*:4,5-*b′*]­dithiophene-4,8-diol (BDT), 9*H*-carbazole (CBZ), 1,4-diketopyrrolo­[3,4-*c*]­pyrrole (DPP)—decorated with enantiopure branched alkyl chains from chiral pool and connected to lateral thiophene peripheries. Their chiroptical properties have been investigated in thin films, showing a variety of different situations, from pure intrinsic ECD and CPL signals to very strong nonreciprocal contributions.^76–84^ However, all of these thiophene-based compounds share the common feature of belonging to the first approach for embedding chirality in π-conjugated materials. Therefore, in this work, we developed two bithiophene-functionalized binaphthyl systems: the open BINOL derivative (a*R*)-**3** and the methylene-bridged BINOL derivative (a*R*)-**6**, functionalized at the C6/C6′ positions with lateral 2,2′-bithiophen-5-yl moieties (Figure 1), which have the merit of extending the π-conjugation and providing significant ECD/CPL signals in the visible region. Both target molecules were easily synthesized and subjected to a complete UV–vis absorption and ECD investigation, first in solution and then as thin films (prepared as neat materials by means of spin coating or drop casting). As a term of comparison, their corresponding synthetic precursors were also studied in a similar way. These differ only in the substituents at the C6/C6′ positions: the unsubstituted BINOLs (a*R*)-**1** (open) and (a*R*)-**4** (methylene-bridged), the 6,6′-dibrominated BINOLs (a*R*)-**2** (open) and (a*R*)-**5** (methylene-bridged). Computational studies allowed us to gain physical insight into the observed (chiro)­optical properties, emphasizing the crucial role of thiophene rings as C6/C6′ substituents. In the final part, we also explored the possibility of chiroptical induction, by means of preliminary ECD and CPL measurements performed on thin films of blends of the achiral benzothiadiazole (BTD) dye **7** with small percentages (0.5–5.0 mol %) of methylene-bridged bithiophene-BINOL derivative (a*R*)-**6** as chiral inducer.

**Figure 1:**
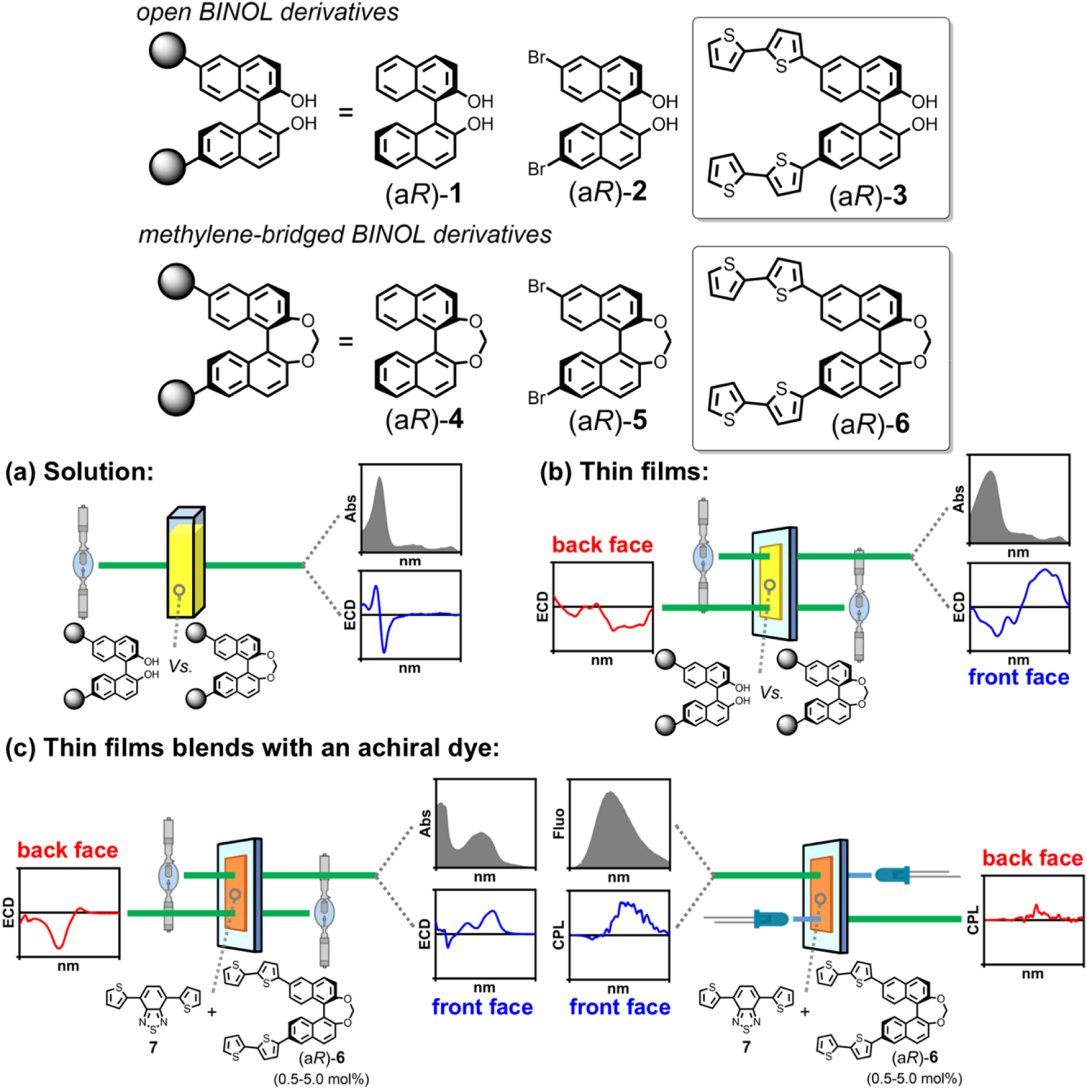
Aim of the work. Two enantiopure binaphthyl systems were investigated: the open BINOL derivative (a*R*)-**3** and the methylene-bridged BINOL derivative (a*R*)-**6**, functionalized at the C6/C6′ positions with lateral 2,2′-bithiophen-5-yl moieties. Both target molecules were subjected, together with their corresponding synthetic precursors (unsubstituted BINOL (a*R*)-**1** and (a*R*)-**4**; 6,6′-dibrominated BINOL (a*R*)-**2** and (a*R*)-**5**) to a comparative study by means of UV–vis absorbance and ECD spectroscopies, performed in (a) solution and (b) thin films as neat materials, also in combination with computational investigations. Finally, (c) preliminary ECD and CPL measurements were performed on thin films of blends of achiral BTD dye **7** with methylene-bridged BINOL derivative (a*R*)-**6** as chiral inducer.

## 2 Results and Discussion

### 2.1 Synthesis

All of the target molecules were synthesized starting from commercially available BINOL (a*R*)-**1**, as depicted in Scheme 1. For the preparation of the other open BINOL derivatives, (a*R*)-**1** was first subjected to a bromination reaction according to a literature protocol of Smith et al.,^85^ carried out with molecular bromine (3 equiv), in acetonitrile at 0 °C, affording quantitatively the corresponding 6,6′-dibromo BINOL (a*R*)-**2**. Then, a Suzuki–Miyaura cross-coupling reaction of (a*R*)-**2** with 2,2′-bithiophene-5-boronic acid pinacol ester (5 equiv), carried out in aqueous 2 M K_2_CO_3_/1,4-dioxane and in the presence of PdCl_2_(PPh_3_)_2_ (8 mol %) as the catalyst, afforded the corresponding open bithiophene-BINOL derivative (a*R*)-**3** in 29% yield after chromatographic purification. The modest yield of the product could be tentatively attributed to the presence of the −OH groups, which are susceptible to unwanted deprotonation under the basic conditions adopted for the cross-coupling step, followed by other side reactions. Moving on to the preparation of methylene-bridged BINOL derivatives **4–6**, the starting material (a*R*)-**1** was first subjected to an alkylation reaction with diiodomethane to give the corresponding methylene-bridged compound (a*R*)-**4**. In particular, according to a modified literature procedure,^86^ BINOL (a*R*)-**1** was treated with CH_2_I_2_ (3 equiv), in refluxing acetonitrile under basic conditions (K_2_CO_3_, 6 equiv), affording product (a*R*)-**4** in 79% yield. Since preliminary bromination attempts of intermediate (a*R*)-**4** were all unsuccessful, we decided to also synthesize 6,6′-dibromomethylene-bridged BINOL (a*R*)-**5** starting from the corresponding open system (a*R*)-**2**, by means of an alkylation reaction with diiodomethane. The protocol was very similar to that used for the synthesis of (a*R*)-**4**, except for the solvent (acetone instead of acetonitrile), and product (a*R*)-**5** was isolated in a 31% yield after column chromatography purification. Finally, (a*R*)-**5** was subjected to the Suzuki–Miyaura reaction with 2,2′-bithiophene-5-boronic acid pinacol ester (5 equiv), carried out again in aqueous 2 M K_2_CO_3_/1,4-dioxane and with PdCl_2_(PPh_3_)_2_ (8 mol %) as the catalyst: in this case, the methylene-bridged BINOL derivative (a*R*)-**6** was isolated as a pure product in 64% yield.

**Scheme 1:**
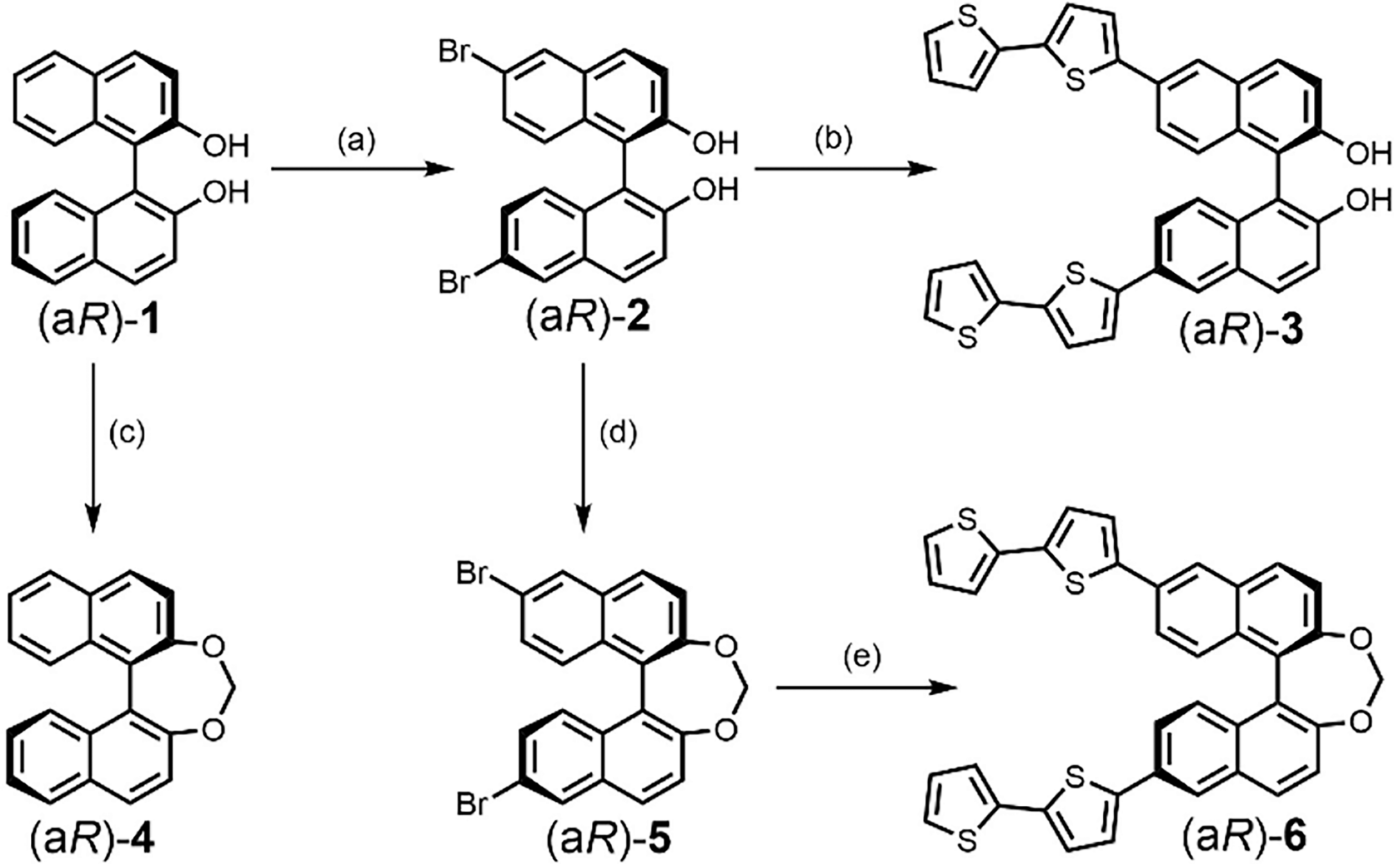
Synthesis of Enantiopure Open BINOL Derivatives **2–3** and Methylene-Bridged BINOL Derivatives **4–6** from Commercially Available BINOL (a*R*)-**1** ^a^

a Conditions: (a) Br_2_ (3 equiv), acetonitrile, 0 °C (ice bath), 3 h (yield: 99%); (b) 2,2′-bithiophene-5-boronic acid pinacol ester (5 equiv), PdCl_2_(PPh_3_)_2_ (8 mol %), K_2_CO_3_ 2M, 1,4-dioxane, reflux (oil bath), 48 h (yield: 29%); (c) CH_2_I_2_ (3 equiv), K_2_CO_3_ (6 equiv), acetonitrile, reflux (oil bath), 24 h (yield: 79%); (d) CH_2_I_2_ (3 equiv), K_2_CO_3_ (6 equiv), acetone, reflux (oil bath), 24 h (yield: 31%); (e) 2,2′-bithiophene-5-boronic acid pinacol ester (5 equiv), PdCl_2_(PPh_3_)_2_ (8 mol %), K_2_CO_3_ 2M, 1,4-dioxane, reflux (oil bath), 48 h (yield: 64%)

### 2.2 Optical and Chiroptical Investigation in Solution

With the six target molecules in hand, we started their systematic optical and chiroptical investigation in solution (Figure 2). UV–vis absorbance and ECD spectra were recorded for 1.0·10^–5^ M solutions in acetonitrile, chosen as the solvent because of its far UV cutoff (190 nm), which allows us to reliably measure both UV–vis absorbance and ECD spectra above this cutoff. Only in the case of methylene-bridged derivative (a*R*)-**6**, spectra were measured in CH_2_Cl_2_ solutions (UV cutoff: 233 nm) due to its poor solubility in acetonitrile. The most relevant (chiro)­optical data are summarized in Table 1, while in the Supporting Information, more detailed data can be found: Figures S1–S6 contain Lambert–Beer plots and Figures S7–S12 the *g*_abs_ vs wavelength plots.

**Figure 2:**
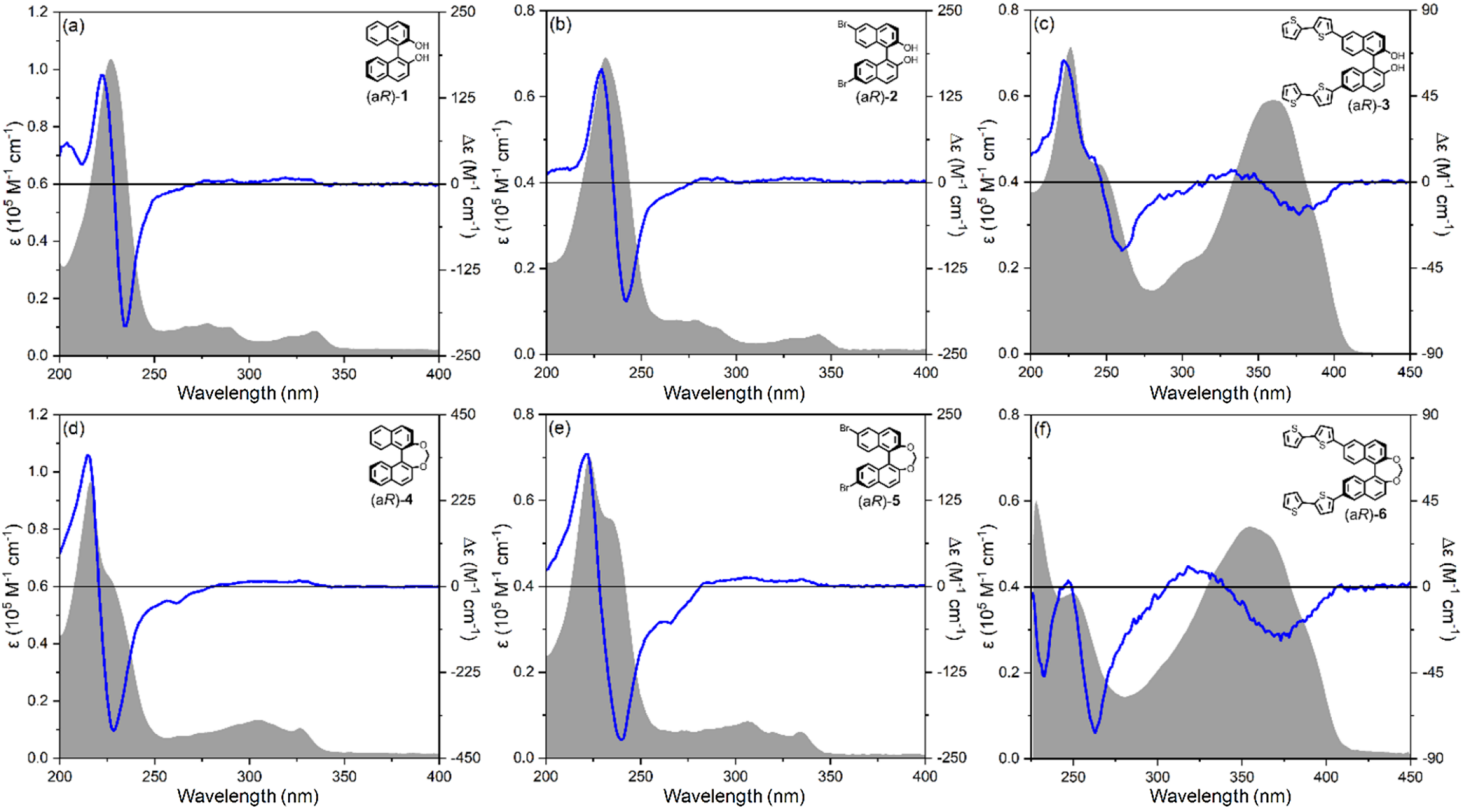
Optical and chiroptical investigation of enantiopure open BINOL derivatives **1–3** and methylene-bridged BINOL derivatives **4–6** in solution. UV–vis absorbance (gray profiles) and ECD (blue lines) spectra of (a) (a*R*)-**1** in acetonitrile; (b) (a*R*)-**2** in acetonitrile; (c) (a*R*)-**3** in acetonitrile; (d) (a*R*)-**4** in acetonitrile; (e) (a*R*)-**5** in acetonitrile; (f) (a*R*)-**6** in CH_2_Cl_2_. Cell length 1 cm; sample concentration 10^–5^ M.

**Table 1:** Optical and Chiroptical Properties of Enantiopure Open BINOL Derivatives **1–3** and Methylene-Bridged BINOL Derivatives **4–6** in Acetonitrile Solution

	λ_abs_^max^ ^a^	ε^max^ ^b^	λ_ECD_^max^ ^c^	*g*_abs_^max^ ^d^	*A*_CD_ ^e^	Δλ^obs^ ^f^		Δλ^true^ ^g^	
dye	(nm)	(10^4^ M^–1^ cm^–1^)	(nm)	(·10^–2^)	(M^–1^ cm^–1^)	(nm)	(eV)	(nm)	(eV)
(a*R*)-**1**	**227**	10.6	222.6	+0.18	365.6	12.0	0.285	6.2	0.148
	278	^h^	234.6	–0.30					
	334	^h^							
(a*R*)-**2**	**231**	5.7	228.8	+0.25	338.3	13.2	0.296	7.8	0.177
	278	^h^	242.0	–0.36					
	343	^h^							
(a*R*)-**3**	**226**	6.9	221.7	+0.097	99.6	38.3	0.824	^h^	^h^
	359	5.8	260.0	–0.12					
			376.8	–0.037					
(a*R*)-**4**	**216**	9.9	215.0	+0.37	721.0	13.5	0.341	7.8	0.200
	306	^h^	228.5	–0.62					
	327	^h^							
(a*R*)-**5**	**223**	8.1	221.4	+0.28	415.9	18.6	0.434	13.2	0.313
	306	^h^	240.0	–0.47					
	334	^h^							
(a*R*)-**6** ^i^	249	^h^	263.0	–0.31	n.a.	n.a.	n.a.	n.a.	n.a.
	**355**	7.4	317.9	+0.036					
			373.0	–0.059					

a Wavelength of maximum light absorbance (in bold, the highest value).

b Molar extinction coefficient corresponding to λ_abs_^max^.

c Wavelength of maximum ECD intensity.

d Maximum dissymmetry factor value, defined as *g*_abs_ = (mdeg of ellipticity/32980)/Absorbance, calculated at λ_ECD_^max^.

e Amplitude of the ^1^B_b_ ECD couplet, defined as the difference between the maximum (peak) and the minimum (trough) ECD values of the couplet.

f Apparent Davydov splitting of the ^1^B_b_ ECD couplet, defined as the wavelength separation between the maximum and the minimum values of the couplet components experimentally observed.

g True Davydov splitting of the ^1^B_b_ ECD couplet, calculated by fitting the two experimental couplet components with two Gaussian curves.

h Not calculated.

i Optical and chiroptical properties recorded in dichloromethane solution.

The solution UV–vis and ECD spectra of BINOL (a*R*)-**1** and its derivatives (a*R*)-**2**, (a*R*)-**4**, and (a*R*)-**5** fit the well-known behavior of 1,1′-binaphthyl derivatives.^43,87,88^ In the UV–vis absorbance spectra (Figure 2a,b,d,e, gray profiles), three major bands are visible from left to right corresponding to ^1^B_b_, ^1^L_a_, and ^1^L_b_ transitions in Platt’s nomenclature,^89,90^ respectively, polarized along the long, short, and long axis in the parent naphthalene chromophore. ECD spectra (Figure 2a,b,d,e, blue lines) display negative couplets between 200 and 250 nm, arising from the degenerate exciton coupling between the ^1^B_b_ transitions. The couplet sign is in accord with (a*R*) configuration and a cisoid arrangement between the two naphthalene rings, or at least with θ < 110°, and its crossover point (i.e., where it crosses the zero line) roughly coincides with the UV–vis absorbance maximum. Additional ECD bands are found in correspondence with ^1^L_a_ and ^1^L_b_ transitions, all with a positive sign. The major effect of the methylene bridge on ECD spectra is evident in the wavelength separation between the ^1^B_b_ couplet components, which increases from Δλ^obs^ = 12.0 nm for (a*R*)-**1** to Δλ^obs^ = 13.5 nm for (a*R*)-**4**, in keeping with theoretical predictions on the dependence of Δλ^obs^ on the dihedral angle θ.^43,46^ Because of reduced mutual cancellation of oppositely signed bands, this also produces a stronger couplet,^87,88^ with amplitude *A*_CD_ (defined as peak-to-trough ΔΔε) increasing from 365.6 M^–1^ cm^–1^ for open BINOL (a*R*)-**1** to 721.0 M^–1^ cm^–1^ for methylene-bridged BINOL (a*R*)-**4**.

A similar increase in the wavelength splitting can be found in the dibrominated open BINOL derivative (a*R*)-**2** (Δλ^obs^ = 13.2 nm), due in this case not to a conformational but rather to an electronic effect of the 6-Br substituent, which tilts the ^1^B_b_ electric dipole transition moment (EDTM) away from the naphthalene long axis (see Figure S13 of the Supporting Information). It has been shown that such an effect contributes positively to the Davydov splitting, i.e., the energy separation between the exciton-split excited states.^91^ As a result of the combination of both conformational and electronic effects, the corresponding methylene-bridged compound (a*R*)-**5** reaches the highest Δλ^obs^ value of 18.6 nm. It must be recalled that the apparent splitting (Δλ^obs^) is always larger than the theoretical one (Δλ^true^). The true Davydov splitting Δλ^true^ values, extracted by fitting experimental couplets with two Gaussian components (Figures S7, S8, S10, and S11 of the Supporting Information), follow the same trend as the apparent splitting Δλ^obs^ values (Table 1). In addition to the change in the appearance and intensity of the ^1^B_b_ ECD couplet, larger Davydov splittings in methylene-bridged compounds (a*R*)-**4** and (a*R*)-**5** also produce a broadening of the main UV–vis absorbance band and the emergence of a long-wavelength shoulder. Moreover, the ^1^L_a_ band (both in UV–vis and ECD spectra) is red-shifted and largely superimposed with the ^1^L_b_ band. Apart from these relatively minor effects, UV–vis absorbance and ECD spectra of compounds (a*R*)-**1**, (a*R*)-**2**, (a*R*)-**4**, and (a*R*)-**5** are all quite consistent with each other (Figure 2a,b,d,e).

On the contrary, the introduction of bithiophene moieties on the C6/C6′ positions in compounds (a*R*)-**3** and (a*R*)-**6** is responsible for a dramatic change in the photophysical properties in solution. The UV–vis absorbance spectrum of (a*R*)-**3** (Figure 2c, gray profile) revealed two broad bands of similar intensity: one at high energy, between 200 and 275 nm, with maximum at 226 nm (ε = 6.9·10^4^ M^–1^ cm^–1^) and a shoulder at around 245 nm; the other at lower energy, between 275 and 400 nm, centered at 359 nm and a shoulder at around 305 nm. The low-energy band is related to electronic transitions mainly involving the two lateral bithienyl scaffolds (see the computational analysis below). They also produce a weak negative ECD exciton couplet between 300 and 400 nm (Figure 2c, blue line), apparently related to a negative chirality between EDTMs. In the high-energy region between 200 and 300 nm, two further bands of opposite sign appear. However, the very large apparent splitting (Δλ^obs^ = 38.3 nm) and the relative position of ECD maxima with respect to UV–vis absorbance ones suggest that this is not a simple exciton couplet but actually emerges from the combination of several transitions.

Further deviation from the typical spectral behavior of 1,1′-binaphthyl derivatives was observed for the methylene-bridged compound (a*R*)-**6**. UV–vis absorbance and ECD spectra were recorded in CH_2_Cl_2_ instead of acetonitrile; therefore, it was not possible to explore the window between 200 and 225 nm. Nevertheless, in the absorbance spectrum (Figure 2f, gray profile), part of the highest energy band was still observed between 225 and 275 nm, with the shoulder centered at 249 nm. The major band has its maximum at 355 nm (ε = 7.4·10^4^ M^–1^ cm^–1^), close to its open analogue (a*R*)-**3**. In the solution ECD spectrum of (a*R*)-**6** (Figure 2f, blue line), the corresponding ECD couplet observed between 300 and 415 nm was slightly more intense than that of (a*R*)-**3**. At high energy, the typical signature of 1,1′-binaphthyl derivatives is no longer recognizable, and two negative bands appear with maxima at 263 and 232 nm. To confirm that the solvent change (from acetonitrile to CH_2_Cl_2_) in (a*R*)-**6** did not result in significant variations in optical and chiroptical properties, we repeated the UV–vis absorbance and ECD spectra measurements of enantiopure open BINOL derivatives **1–3** and methylene-bridged BINOL derivatives **4–5** also in CH_2_Cl_2_ solution (Figure S14 in the Supporting Information). As expected, no significant differences were observed.

A computational analysis based on density functional theory (DFT) and time-dependent DFT (TD-DFT) was performed on all of the compounds investigated. The main aim of this analysis was to justify the differences observed between simple BINOL derivatives (a*R*)-**1**/(a*R*)-**2** and (a*R*)-**4**/(a*R*)-**5** and their 6,6′-di­([2,2′-bithiophen]-5-yl) derivatives (a*R*)-**3** and (a*R*)-**6**. To restrict the number of investigated conformers, the rotamerism around the C2–OH and C2′–OH bonds was neglected, and only the lowest-energy rotamers were considered (with each O–H directed toward the other naphthalene ring). Similarly, a single conformer was considered for the biaryl torsion θ, thus overlooking the possible impact of libration motions with broad energy wells. With such approximations, for compounds (a*R*)-**1**, (a*R*)-**2**, (a*R*)-**4**, and (a*R*)-**5**, a single conformer was considered, optimized at the B3LYP-D3BJ/6–311+G­(d,p) level and including the SMD solvent model for acetonitrile, endowed with *C*_2_ symmetry. The structures are shown in the insets of Figure 3. Vertical excitations were calculated at the CAM-B3LYP/def2-TZVP and B3LYP/def2-TZVP levels, including the IEF-PCM solvent model for acetonitrile. The results obtained with the two functionals were consistent; therefore, only CAM-B3LYP results are discussed here for their superior performance. Calculated UV–vis absorbance and ECD spectra (Figure 3) fit well experimental spectra (after applying a standard wavelength correction^92^ and a small intensity scaling), in agreement with previous TD-DFT calculations of BINOL derivatives.^93–96^ The main discrepancy is observed in the long-wavelength region, due to the known difficulty of DFT to describe ^1^L_a_ states of aromatic compounds.^97^ Another apparent problem is the overestimation of the ^1^B_b_ Davydov splitting for compound (a*R*)-**2** and a consequent overestimation of the couplet amplitude. Nevertheless, the transitions responsible for the major UV–vis absorbance and ECD bands are safely assigned to exciton-split ^1^B_b_ transitions, as witnessed by hole–electron plots (Figure 3).

**Figure 3:**
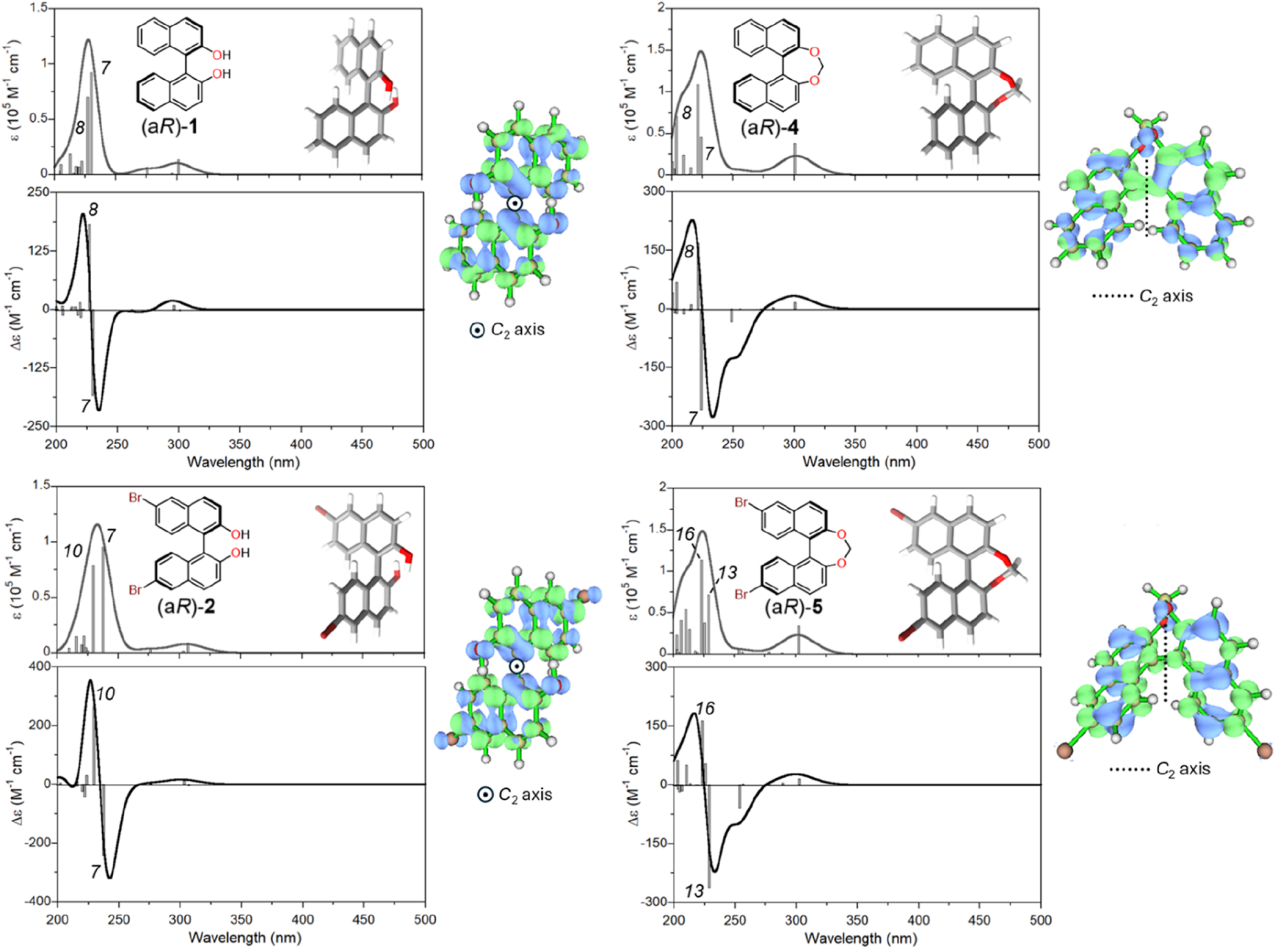
TD-DFT calculated UV–vis absorbance and ECD spectra of compounds (a*R*)-**1**, (a*R*)-**2**, (a*R*)-**4**, and (a*R*)-**5** at the CAM-B3LYP/def2-TZVP//B3LYP-D3BJ/6–311+G­(d,p) level including PCM for acetonitrile. Vertical bars represent oscillator and rotational strengths; numbers in italics indicate the transitions corresponding to exciton-split ^1^B_b_ components. Plotting parameters: Gaussian width σ = 0.25 eV; wavelength correction of +10 nm (compounds (a*R*)-**1** and (a*R*)-**2**) or +5 nm (compounds (a*R*)-**3** and (a*R*)-**4**); intensity scaling factor = 1.5. Inset: DFT-optimized structure for each compound. On the right side, hole (blue) and electron (green) densities are shown for the low-energy ^1^B_b_ component (isovalue 0.02).

In the case of compounds (a*R*)-**3** and (a*R*)-**6**, we anticipated that the rotamerism around the C6-thiophene and thiophene-thiophene bonds (see colored curved arrows in Figure 4) would strongly impact the calculated properties. Therefore, we sampled these torsional angles by taking into account the 8 possible distinct conformers (see Figures S15 and S16 and Tables S1 and S2 of the Supporting Information). Calculated UV–vis absorbance and ECD spectra in Figure 4 were evaluated as the Boltzmann averages (300 K) over 8 conformers for each compound. A perfect reproduction of experimental spectra would require more extensive conformational sampling. Still, the agreement is satisfactory and allows rationalization of the main effects produced by the bithienyl substituents. In the high-energy regions, a large number of transitions with strong oscillator and rotational strengths contribute to the final spectra, which is at odds with simpler derivatives (a*R*)-**1**/(a*R*)-**2** and (a*R*)-**4**/(a*R*)-**5** where the exciton-split ^1^B_b_ transitions dominate. The reason for the large discrepancy seen in the experimental spectra of the two sets of compounds is therefore obvious. In particular, one should refrain from recognizing a simple exciton couplet in Figure 2c for (a*R*)-**3**. In the long-wavelength region, the calculations nicely reproduce both the negative sign, band position, and the relative intensity of the ECD couplets (compare Figures 4 with [Fig fig2]c–f). Analysis of hole–electron densities allows recognizing two transitions arising from the exciton coupling of π–π* transitions localized on the bithiophene moieties. Very interestingly, the amplitude, the position, and even the sign of the couplet vary considerably among the various conformers (see expansions in Figure 4) because the rotamerism around the C6-thiophene and thiophene-thiophene bonds affects the orientation of the EDTMs localized on the bithiophene moieties. In turn, this is a reason for the resulting overall weak ECD couplets found both theoretically and experimentally above 300 nm.

**Figure 4:**
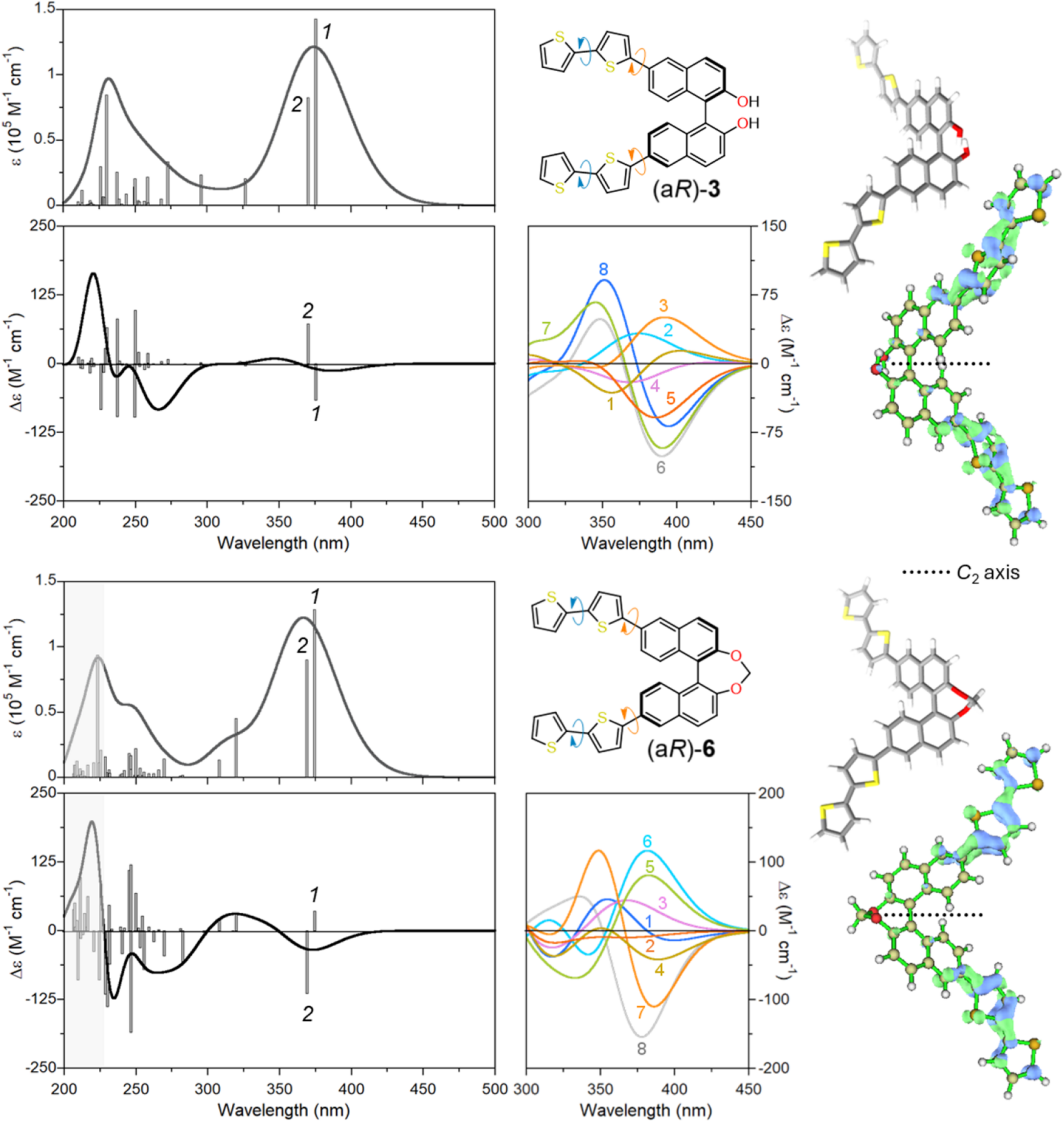
TD-DFT calculated UV–vis absorbance and ECD spectra of compounds (a*R*)-**3** and (a*R*)-**6** at the CAM-B3LYP/def2-TZVP//B3LYP-D3BJ/6–311+G­(d,p) level including PCM for acetonitrile (compound (a*R*)-**3**) or DCM (compound (a*R*)-**6**); Boltzmann average over 8 conformers in each case. Vertical bars represent oscillator and rotational strengths for the lowest-energy C_2_-symmetric structure shown on the right; numbers in italics indicate π–π* transitions localized on the bithiophene moieties. Plotting parameters: Gaussian width σ = 0.25–0.3 eV; wavelength correction of +15 nm (compound (a*R*)-**3**) or +6 nm (compound (a*R*)-**6**); no intensity scaling. The shaded area in the spectra of (a*R*)-**6** is not accessible by experiment. On the right side, hole (blue) and electron (green) densities are shown for the lowest-energy band (isovalue 0.02). The expansions show calculated ECD spectra for the 8 conformers (due to rotamerism around the curved arrows) in the low-energy region, numbered according to their relative population from the largest (1) to the smallest (8) (see Figures S15 and S16).

### 2.3 Optical and Chiroptical Investigation in Thin Films

As already mentioned in the Introduction, although the chiroptical properties of both open and chain-bridged BINOL derivatives have been extensively studied in thin films, most of these studies involve blends in poly­(methyl methacrylate) (PMMA) or, alternatively, neat materials prepared by ultrahigh-vacuum evaporation. Chiroptical studies of thin films of simple BINOL derivatives prepared as neat materials by solution-processable techniques (such as spin coating or drop casting) are very rare. Considering the potential application of these systems in organic optoelectronics, we decided to investigate UV–vis absorbance and ECD spectra in thin films as neat materials (Figure 5 and Table 2). More in detail, samples were prepared by spin coating, depositing 100 μL of a 2·10^–2^ M CH_2_Cl_2_ solution of each dye on quartz plates; only in the case of methylene-bridged derivative (a*R*)-**6**, thin films were fabricated by drop casting a 3·10^–3^ M CH_2_Cl_2_ solution because of its incomplete solubility at higher concentrations. Even in the thin film state, significant differences were found between the two families of BINOL derivatives (open vs methylene-bridged) and, for each of them, depending on the substituents present on the C6/C6′ positions (hydrogen or bromine atoms vs bithiophene moieties).

**Figure 5:**
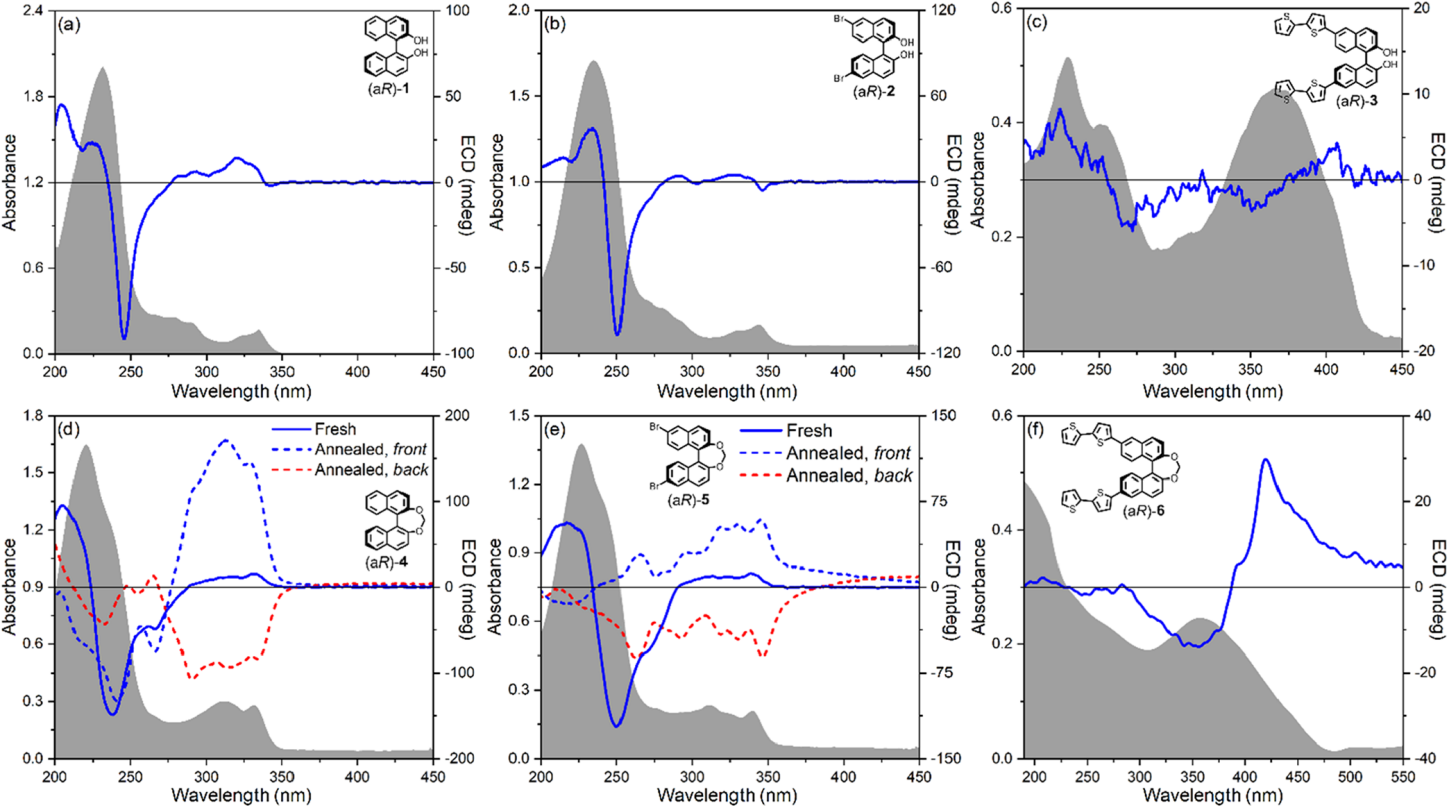
Optical and chiroptical investigation of enantiopure open BINOL derivatives **1–3** and methylene-bridged BINOL derivatives **4–6** in thin films. UV–vis absorbance (gray profiles) and ECD (blue continuous lines) spectra of as-cast samples of (a) (a*R*)-**1**, (b) (a*R*)-**2**, (c) (a*R*)-**3**, (d) (a*R*)-**4**, and (e) (a*R*)-**5**, prepared by spin coating from a 2·10^–2^ M CH_2_Cl_2_ solution; (f) (a*R*)-**6**, prepared by drop casting from a 3·10^–3^ M CH_2_Cl_2_ solution. In panels (d) and (e), blue and red dashed lines are the ECD spectra recorded, respectively, for the front face and the back face of the same samples after annealing (for (a*R*)-**4**: CH_2_Cl_2_, 5 min; for (a*R*)-**5**: 120 °C, 10 min).

**Table 2:** Optical and Chiroptical Properties of Enantiopure Open BINOL Derivatives **1–3** and Methylene-Bridged BINOL Derivatives **4–6** in Thin Films, Prepared by a Spin Coating Technique from a 2·10^–2^ M CH_2_Cl_2_ Solution

dye	annealing^a^	λ_abs_^max^ ^b^ (nm)	λ_ECD_^max^ ^c^ (nm)	*g*_abs_^max^ ^d^ (·10^–2^)	ρ^e^
(a*R*)**-1**	no	**231**	222	+ 0.038	n.a.
		335	245	– 0.22	
(a*R*)**-2**	no	**235**	234	+ 0.066	n.a.
		343	250	– 0.29	
(a*R*)**-3**	no	**229**	224	+ 0.051	n.a.
		368	272	– 0.067	
(a*R*)**-4**	no	**221**	205	+ 0.26	n.a.
		332	238	– 0.38	
	CH_2_Cl_2_, 5 min	**221**	*front:* 313	+ 1.24	1.92
		332	*back:* 290	– 0.93	
(a*R*)**-5**	no	**227**	217	+ 0.15	n.a.
		340	250	– 0.43	
	120 °C, 10 min	**227**	*front:* 346	+ 1.23	2.46
		340	*back:* 346	– 1.31	
(a*R*)**-6** ^f^	no	**357**	356	– 0.17	n.a.
			420	+ 0.69	

a Experimental details of annealing (if any) performed before optical and chiroptical measurements.

b Wavelength of maximum light absorbance (in bold, the highest value).

c Wavelength of maximum ECD intensity.

d Maximum dissymmetry factor value, defined as *g*_abs_ = (mdeg of ellipticity/32980)/Absorbance, calculated at λ_ECD_^max^.

e Ratio of the integrated areas of the absolute value of semidifference (∫|LDLB|) and semisum (∫|CDiso|).

f Thin films were prepared by drop casting technique from a 3·10^–3^ M CH_2_Cl_2_ solution.

The UV–vis absorbance spectrum of (a*R*)-**1** in thin film (Figure 5a, gray profile) was roughly similar to that in acetonitrile solution. The corresponding ECD spectrum (Figure 5a, blue line) exhibited an asymmetric negative exciton couplet between 200 and 270 nm, also in keeping with the solution spectrum. With respect to the latter, a very weak extra Cotton effect appears just below 350 nm, which could be interpreted as the low-energy side of an asymmetric ^1^L_b_ couplet with crossover at 337 nm. Interestingly, this band has been already reported by Kartouzian et al. for films of (*R*)-BINOL prepared by ultrahigh-vacuum evaporation, after 1 h of aging. Together with the broadening of the main absorbance band and the nonsymmetric appearance of the ^1^B_b_ couplet, the extra band might be a manifestation of intermolecular exciton coupling between closely lying aggregated BINOL moieties, similarly to what occurs for ECD spectra of microcrystalline samples.^98,99^

The 6,6′-dibrominated open BINOL derivative (a*R*)-**2** showed optical and chiroptical properties in thin films (Figure 5b, gray profile and blue line) very similar to its nonbrominated homologue (a*R*)-**1**, including the extra low-energy band. The data clearly suggest that in thin films, molecules of the open BINOL derivative (a*R*)-**2** are arranged in a very similar way to (a*R*)-**1**.

On the contrary, the introduction of bithiophene moieties at the C6/C6′ positions was responsible for a substantial change in thin film properties. In particular, the ECD spectrum of (a*R*)-**3** (Figure 5c, blue line) showed a marked decrease in signal intensity compared with its open BINOL homologues (a*R*)-**1** and (a*R*)-**2**, with maximum dissymmetry factor values *g*_abs_ about 1 order of magnitude lower (see Table 2 and Figures S17–S19). Nevertheless, two consecutive couplets seem to be present: the high-energy couplet is negative (similar to the corresponding ECD band of compounds (a*R*)-**1** and (a*R*)-**2**), while the low-energy couplet is positive. In the light of computational results, we interpret the thin film ECD spectrum of (a*R*)-**3** as the result of a conformationally disordered situation where the contributions from multiple conformers tend to cancel each other.

A further point to emphasize for all open BINOL compounds **1–3** is that the ECD profiles recorded for both faces of the sample (front and back) were almost perfectly coincident, thus indicating the presence of only intrinsic chiroptical signals due to molecular (or possibly first-order supramolecular) 3D chirality. At the same time, no significant variations in the ECD spectra were observed upon postdeposition treatments such as solvent or thermal annealing, suggesting stability of the solid-state arrangement. In this regard, completely different (and, to some extent, unexpected) chiroptical behavior was found for thin films of methylene-bridged BINOL derivatives (a*R*)-**4** and (a*R*)-**5**. On the one hand, freshly prepared thin films of both compounds still showed reciprocal ECD spectra (Figure 5d,e, blue lines), consisting of a strong asymmetric negative couplet between 200 and 285 nm and a further positive weaker band between 280 and 350 nm. These correspond to the two main bands present in the UV–vis absorbance spectra (Figure 5d,e, gray profiles) and also parallel solution spectra (Figure 2d,e). However, when the same samples were subjected to very short postdeposition treatments (for compound (a*R*)-**4**, 5 min of solvent annealing in a closed chamber with CH_2_Cl_2_ vapors; for compound (a*R*)-**5**, 10 min of thermal annealing in an oven at 120 °C), a strong nonreciprocal contribution appeared in the ECD spectra. In detail, (a*R*)-**4** showed a broad positive band between 275 and 350 nm (λ_ECD_^max^ = 313 nm) for the front face, and a negative, more structured band in the same range (λ_ECD_^max^ = 290 nm) for the back face, corresponding to a ρ ratio value of 1.92. Compound (a*R*)-**5** revealed an even more predominant LDLB term, with almost mirror-like dichroic profiles for the front and back faces, associated with a ρ ratio of 2.46 (Figure 5d,e, dashed lines). This evolution of ECD signals from pure intrinsic CDiso to predominant nonreciprocal LDLB upon annealing was also accompanied by an increase in dissymmetry factor *g*_abs_ values (see Table 2 and Figures S20–S23). While molecules with open BINOL systems (a*R*)-**1**/(a*R*)-**2** can adopt a stable solid-state arrangement immediately upon deposition as thin films, the methylene-bridged derivatives (a*R*)-**4**/(a*R*)-**5** are initially trapped in kinetically favored aggregation states, exhibiting 3D (supra)­molecular chirality. However, with increased molecular mobility, these systems evolve into a more thermodynamically stable organization, characterized by long-range 2D chirality. Very interesting is the finding that ECD spectra dominated by LDLB, apart from being nonreciprocal, have no resemblance with CDiso traces: the most intense ECD signals are associated with ^1^L_a_/^1^L_b_ transitions rather than ^1^B_b_ ones. This confirms the substantially different character of the two phenomena.^100^

As mentioned above, thin films of compound (a*R*)-**6** were prepared by drop casting. Due to its limited solubility in CH_2_Cl_2_, we were able to prepare a solution of (a*R*)-**6** with a maximum concentration of 3·10^–3^ M. Spinning such a solution resulted in excessively thin films with very low absorbance. Therefore, we opted for drop casting, which allows the entire volume of the solution to be deposited on the quartz plate. Similarly to the solution, the UV–vis absorbance spectrum of the thin film (Figure 5f, gray profile) revealed a first prominent band between 300 and 475 nm (λ_abs_^max^ = 357 nm), together with a second stronger band at high energy. Moving on to its ECD spectrum (Figure 5f, blue line), a strong positive couplet appeared between 300 and 550 nm, including a pronounced long-wavelength tailing due to scattering (quite common in drop-casted samples). At high energy, only very weak chiroptical signals were detected (dissymmetry factor *g*_abs_ dropped by about 2 orders of magnitude, see also Figure S24). Thin film ECD spectra suggest for compound (a*R*)-**6** a very different supramolecular arrangement from its open analog (a*R*)-**3**, favoring intermolecular exciton coupling between bithiophene units in a right-handed helical arrangement. A pictorial representation of a small oligomer, compatible with the shape of the aggregate ECD spectra, is displayed in Figure 6.

**Figure 6:**
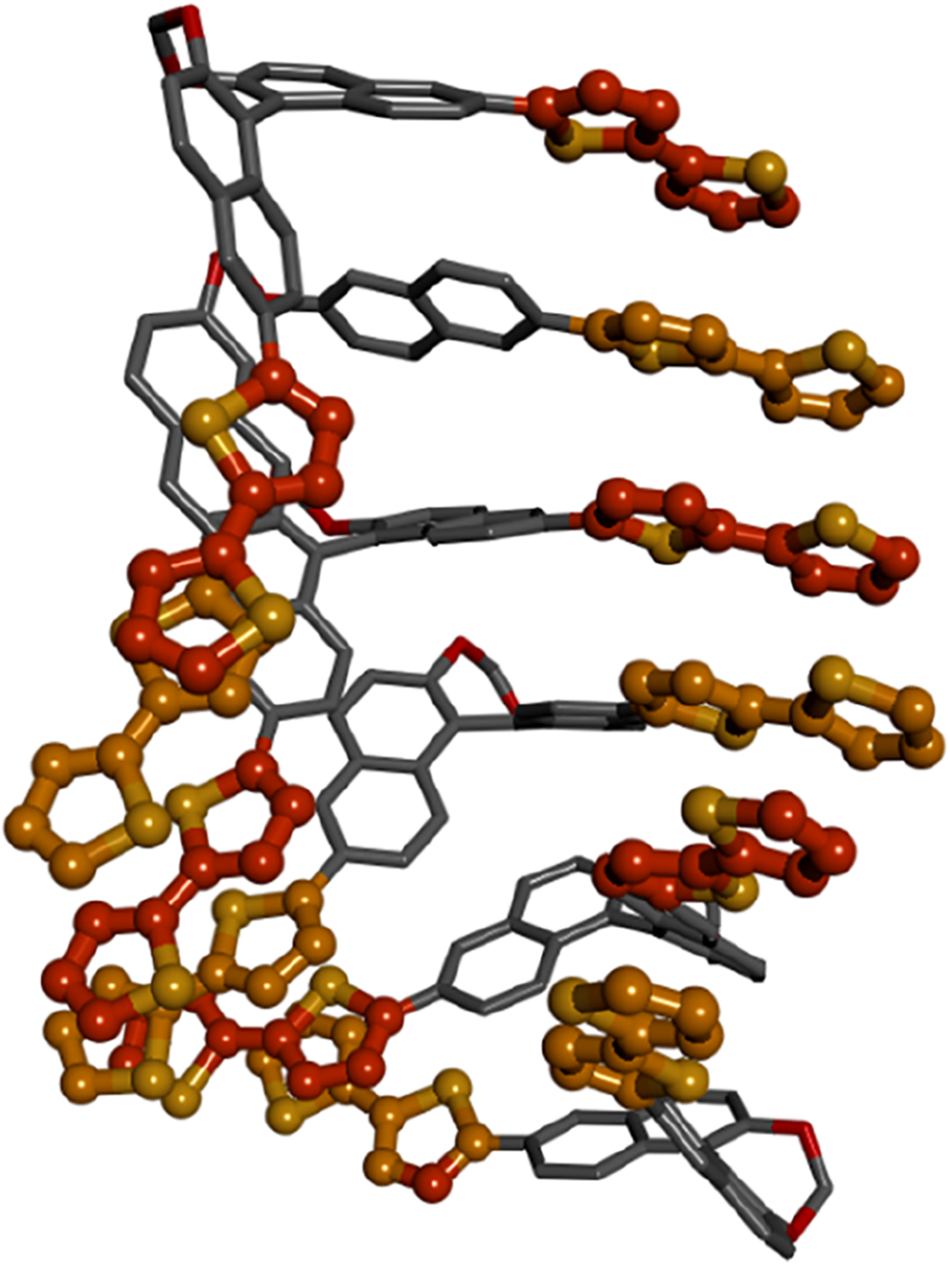
Hexamer of (a*R*)-**6** obtained by molecular mechanics (MMFF force field) optimization from the DFT structure displaying a right-handed helical arrangement of bithiophene units. The BINOL skeleton is shown as gray sticks, while the thiophene rings are shown as orange balls and sticks.

### 2.4 Preliminary Tests of Chiroptical Induction

Given the recent interest in the use of enantiopure BINOL compounds as inducers of chiroptical properties in thin films of achiral π-conjugated small molecules^63,68^ and polymers,^64–67,69–72^ we ran similar tests with our compounds, as well. Thin films of blends of the achiral benzothiadiazole (BTD) dye **7**, synthesized according to a modified literature procedure^101^ (see the Supporting Information for details), with small percentages (0.5–5.0 mol %) of methylene-bridged bithiophene-BINOL derivative (a*R*)-**6** as the chiral inducer, were prepared by the spin coating technique. Among all BINOL compounds investigated in the present work, (a*R*)-**6** was selected based on the results obtained on thin films, indicating a larger propensity for ordered supramolecular structures, and for the presence of bithiophene lateral moieties, which can favor π–π interactions with the thiophene rings of BTD dye **7**, thus facilitating chiral induction.

First, we ascertained the absence of dichroic signals in as-cast thin films of the achiral dye **7**, prepared under the same experimental conditions but in the absence of the chiral inductor (a*R*)-**6** (Figure 7a). Then, we recorded UV–vis absorbance and ECD spectra of as-cast samples of blends with four different amounts of BINOL (a*R*)-**6**: 0.5, 1.0, 2.5, and 5.0 mol % (Figure 7b–e and Table 3). Absorbance profiles were almost independent of the chiral inducer concentration (Figure 7b–e, gray profiles), showing in all of the samples the two broad bands typical of BTD chromophore between 380 and 600 nm and below 380 nm, respectively; however, a hyperchromic effect was observed in the presence of 2.5–5.0 mol % (a*R*)-**6**. The ECD spectra of all blends (Figure 7b–e, blue and red lines) displayed strong chiroptical signals in correspondence with BTD transitions, demonstrating the occurrence of the desired ECD induction effect. Moreover, they exhibited a strong dependence on the chiral inducer concentration, with significant changes not only in signal intensity but also in terms of spectral shape and ρ ratio (Table 3 and Figures S25–S28). These findings clearly demonstrate that the concentration of (a*R*)-**6** plays a crucial role in directing the solid-state organization of dye **7** at different hierarchical levels.

**Figure 7:**
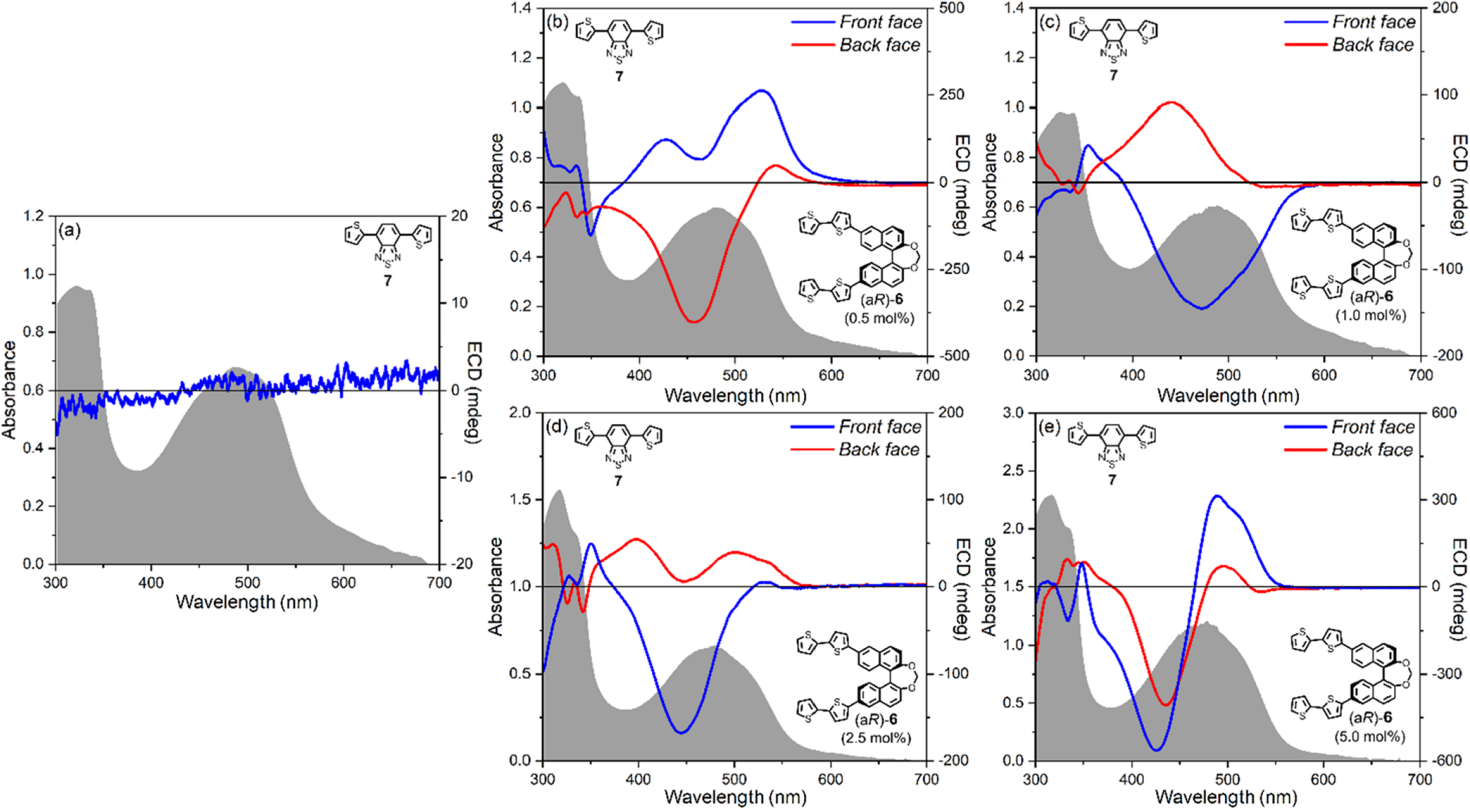
Optical and chiroptical investigation in absorption of blends of the achiral π-conjugated dye **7** with methylene-bridged BINOL chiral inducer (a*R*)-**6** as thin films, prepared by the spin coating technique from a 0.1 M CHCl_3_ solution of **7** with different amounts of (a*R*)-**6**. (a) UV–vis absorbance (gray profile) and ECD (blue line) spectra of as-cast samples of pure achiral π-conjugated dye **7**. UV–vis absorbance (gray profiles) and ECD spectra recorded for the front face (blue lines) and the back face (red lines) of as-cast samples of blends of achiral dye **7** with (b) 0.5 mol %, (c) 1.0 mol %, (d) 2.5 mol %, (e) 5.0 mol % chiral inducer (a*R*)-**6**.

**Table 3:** Chiroptical Properties of Blends of the Achiral π-Conjugated Dye **7** with Methylene-Bridged BINOL Chiral Inducer (a*R*)-**6** as Thin Films, Prepared by the Spin Coating Technique from a 0.1 M CHCl_3_ Solution of **7** with Different Amounts of (a*R*)-**6**

(a*R*)-6 (mol %)	λ_ECD_^max^ (nm)^a^	*g*_abs_^max^ (·10^–2^)^b^	ρ^c^	λ_CPL_^max^ (nm)^d^	*g*_lum_^max^ (·10^–2^)^e^
0.5	*front:* 528	+1.86	1.52		
	*back:* 457	–2.16			
1.0	*front:* 472	–0.68	1.94		
	*back:* 440	+0.55			
2.5	*front:* 445	–0.88	1.57		
	*back:* 399	+0.52			
5.0	*front:* 426	–2.01	0.52	*front:* 593	+6.47
	489	+0.81			
	*back:* 436	–1.27		*back:* 593	+3.04
	496	+0.20			

a Wavelength of maximum ECD intensity.

b Maximum dissymmetry factor value, defined as *g*_abs_ = (mdeg of ellipticity/32980)/Absorbance, calculated at λ_ECD_^max^.

c Ratio of the integrated areas of the absolute value of semidifference (∫|LDLB|) and semisum (∫|CDiso|).

d Wavelength of maximum CPL intensity.

e Maximum luminescence dissymmetry factor value, defined as *g*_lum_ = Δ*I*/*I*, calculated at λ_CPL_^max^.

Since BTD dye **7** is highly fluorescent in the solid state, for the blend containing 5.0 mol % (a*R*)-**6**, we also measured (chiro)­optical properties in emission (Figure 8). The photoluminescence spectrum (Figure 8, gray profile) showed a broad band between 525 and 725 nm, with a maximum centered at 604 nm. This spectrum is very similar, in both shape and intensity, to that of the undoped film (Figure S29 of the Supporting Information), suggesting that the luminescence response is essentially unaffected by the presence of the chiral dopant. More interestingly, the CPL spectrum revealed in the same wavelength range a positive band, with a maximum at 593 nm. However, the signal intensity was remarkably different for the two faces of the sample: the maximum luminescence dissymmetry factor *g*_lum_^max^ value was +6.47·10^–2^ for the front face and +3.04·10^–2^ for the back face (Table 3 and Figure S30). This trend seems to mirror that of the low-energy region (between 460 and 525 nm) in the corresponding ECD spectrum (see Figure 7e, blue and red lines), where we found at 489 nm *g*_abs_^max^ = +0.81·10^–2^ for the front face and *g*_abs_^max^ = +0.20·10^–2^ for the back face.

**Figure 8:**
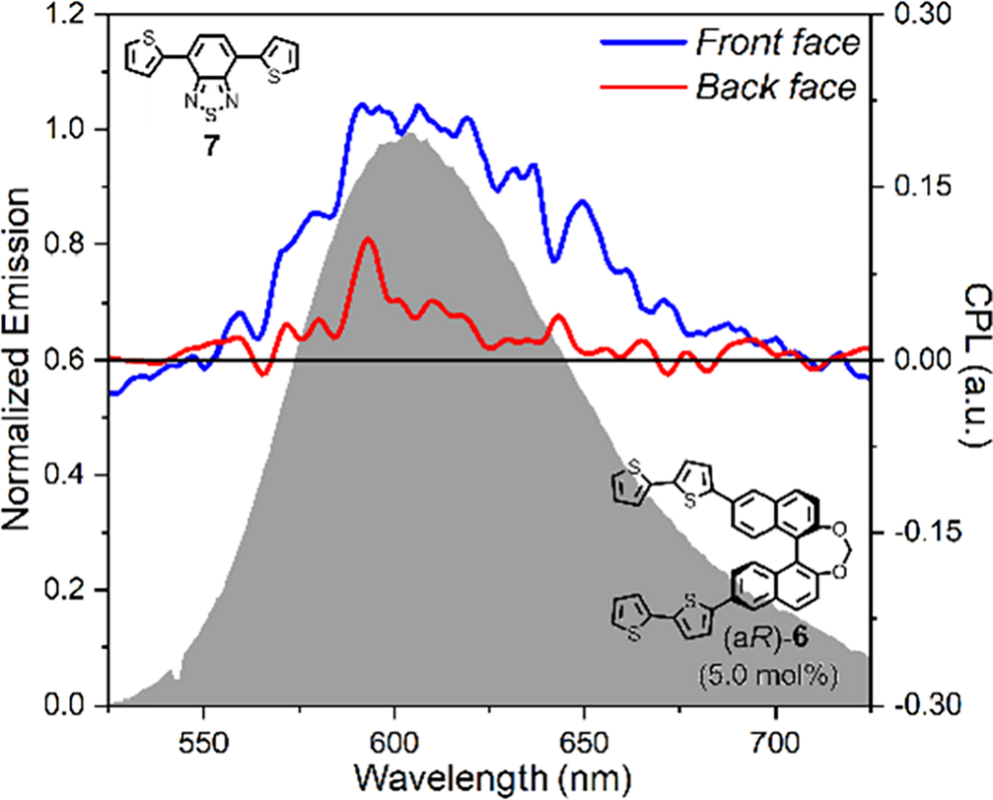
Optical and chiroptical investigation in emission of blends of the achiral π-conjugated dye **7** with methylene-bridged BINOL chiral inducer (a*R*)-**6** as thin films, prepared by the spin coating technique from a 0.1 M CHCl_3_ solution of **7** with 5 mol % (a*R*)-**6**: photoluminescence (gray profile) and CPL spectra recorded for the front face (blue line) and the back face (red line) of as-cast samples. Excitation wavelength: 365 nm.

Although more systematic studies are needed to gain a deeper understanding of the structural features of these blends and to rationalize the chiroptical properties of the resulting thin films, these preliminary results demonstrate the ability of the methylene-bridged BINOL derivative (a*R*)-**6** to act as a chiroptical inducer with potential applications in chiral optoelectronics.

## 3 Conclusions

In conclusion, in this work we synthesized two enantiopure binaphthyl systems: the open BINOL derivative (a*R*)-**3** and the methylene-bridged BINOL derivative (a*R*)-**6**, functionalized at the C6/C6′ positions with lateral 2,2′-bithiophen-5-yl moieties. Both target molecules were subjected, together with their corresponding synthetic precursors (i.e., unsubstituted BINOLs (a*R*)-**1**/(a*R*)-**4** and 6,6′-dibrominated derivatives (a*R*)-**2**/(a*R*)-**5**) to a systematic study of their optical and chiroptical properties. In solution, besides the expected impact of the dihedral angle value θ (open vs methylene-bridged systems), we found a crucial role of the bithiophene moieties at the C6/C6′ positions. Computational studies allowed us to gain physical insight into the observed (chiro)­optical properties. Differences in the chiroptical properties were much more pronounced in thin films as neat materials: the type of chirality present in the solid state (3D-chiral molecular/supramolecular structures vs 2D-chiral micro/mesoscopic domains) and their thermodynamic stability appear to be strongly influenced by both the dihedral angle and the substituents at the C6/C6′ positions. Finally, preliminary studies on chiroptical induction revealed that small amounts (0.5–5.0 mol %) of the methylene-bridged BINOL derivative (a*R*)-**6** in thin films of blends with the achiral BTD dye **7** are capable of generating strong and highly modulable ECD and CPL signals.

This study demonstrates that binaphthyl derivatives with simple chemical structures still hold great potential in the field of chiral organic materials. Their noteworthy (and, in some cases, totally unexpected) chiroptical properties in thin films along with their efficient chiral induction capabilities make them highly attractive candidates for future exploration. These findings pave the way for novel and cutting-edge applications in the growing field of chiral organic optoelectronics.

## Supplementary Material



## Data Availability

The data underlying this study are available in the published article, in its Supporting Information, and openly available in the repository Zenodo at 10.5281/zenodo.17063267.

## Notes

Experimental section: synthetic procedures; experimental section: characterization; computational section; supporting figures; supplementary tables; ^1^H NMR and ^13^C­{^1^H} NMR spectra; Cartesian coordinates; supplementary references (PDF)
