# Supplementary material for: Synthesis and Chiroptical Properties of Bithiophene-Functionalized Open and Methylene-Bridged Binaphthyl Derivatives
Source: J Org Chem. 2025 Oct 2;90(41):14754–68. doi: 10.1021/acs.joc.5c01954 (PMC12538583; doi:10.1021/acs.joc.5c01954)
Supplement: Supplementary file 1 [file jo5c01954_si_001.pdf]

Supporting Information

Synthesis and Chiroptical Properties of Bithiophene-Functionalized Open and Methylene-Bridged Binaphthyl Derivatives

Federico Dini,[a] Giorgia Puntoni,[a] Lilia Tinagli,[a] Lorenzo Di Bari,[a] Gennaro Pescitelli,*[a] Gianluigi Albano*[a,b]

[a] Dipartimento di Chimica e Chimica Industriale, Università di Pisa, Via Giuseppe Moruzzi 13, 56124 Pisa, Italy
[b] Dipartimento di Scienze Chimiche, della Vita e della Sostenibilità Ambientale, Università degli Studi di Parma, Parco Area delle Scienze 17/A, 43124 Parma, Italy

*E-mail: gennaro.pescitelli@unipi.it, gianluigi.albano@unipr.it

Table of contents

Experimental Section: Synthetic Procedures S2
General information S2
Synthesis of open BINOL derivatives 2–3 .... S3
Synthesis of methylene-bridged BINOL derivatives 4–6 .... S5
Synthesis of the achiral π-conjugated dye 7 .... S7
Experimental Section: Characterization S8
Characterization in solution S8
Characterization in thin films S8
Computational Section S10
Supplementary Figures S11
Supplementary Tables S41
^{1}H NMR and ^{13}C{^{1}H} NMR spectra S49
Cartesian Coordinates S61
Supplementary References S85

Experimental Section: Synthetic Procedures

General information

Chemicals. All chemicals were purchased from Merck and used as received without purification. Commercial grade solvents were purified by conventional methods, distilled and stored over activated molecular sieves under nitrogen atmosphere. All the operations under inert atmosphere were carried out using standard Schlenk techniques and employing dry N_2.

Thin-layer chromatography. Rection conversions were monitored by thin-layer chromatography (TLC) analysis on pre-coated silica gel plates ALUGRAM® Xtra SIL G/UV_{254} (0.2 mm) purchased from VWR Macherey-Nagel.

Preparative column chromatography. Column chromatography purifications were carried out with Fluka silica gel, pore size 60 Å, 70-230 mesh, 63-200 µm.

NMR spectroscopy. ^{1}H NMR and ^{13}C{^{1}H} NMR spectra were recorded at room temperature in $CDCl_3$ solution with a JEOL YH400 spectrometer, operating at 400 MHz for ^{1}H and 100 MHz for ^{13}C, using the residual solvent peaks as internal reference; chemical shifts (δ) values are given in parts per million (ppm) and coupling constants (*J*) in Hertz.

Elemental Analysis. Elemental analyses of pure products were performed on an Elementar Vario Micro Cube CHNS-analyzer.

Synthesis of open BINOL derivatives 2–3

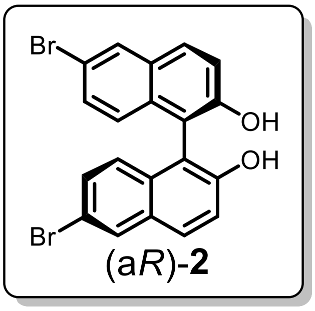

(*R*)-6,6'-Dibromo-[1,1'-binaphthalene]-2,2'-diol ((a*R*)-2). (*R*)-[1,1'-binaphthalene]-2,2'-diol ((a*R*)-**1**) (501 mg, 1.75 mmol) and acetonitrile (10 mL) were mixed together, then the solution was cooled to 0 °C (ice bath) and bromine (269 µL, 5.25 mmol) was added dropwise. [**Caution!** *Bromine must be handled with great care: it is fatal if inhaled and causes severe skin burns and eye damage.*] The reaction mixture was stirred at 0 °C for 3 h, then it was heated at room temperature, hydrolyzed with a 10 wt.% $Na_2S_2O_4$ aqueous solution (30 mL) and extracted with CH_2Cl_2 (3×50 mL). The organic phase was washed with brine (3×50 mL), dried over anhydrous Na_2SO_4 and the solvent was removed under vacuum, to give (*R*)-6,6'-dibromo-[1,1'-binaphthalene]-2,2'-diol ((a*R*)-**2**) (768 mg, 1.73 mmol, yield 99%) as a white solid. Analytical data are in agreement with those previously reported in the literature.[S1] **^{1}H NMR** (400 MHz, $CDCl_3$), δ (ppm): 8.05 (d, *J* = 2.0 Hz, 2H); 7.90 (d, *J* = 9.0 Hz, 2H); 7.40 (d, *J* = 9.0 Hz, 2H); 7.37 (dd, *J* = 9.0, 2.0 Hz, 2H); 6.96 (d, *J* = 9.0 Hz, 2H); 5.02 (s, 2H). **^{13}C{^{1}H} NMR** (100 MHz, $CDCl_3$), δ (ppm): 153.0; 132.1; 130.5; 130.4; 130.3; 126.1; 119.1; 117.7; 116.4; 111.5.

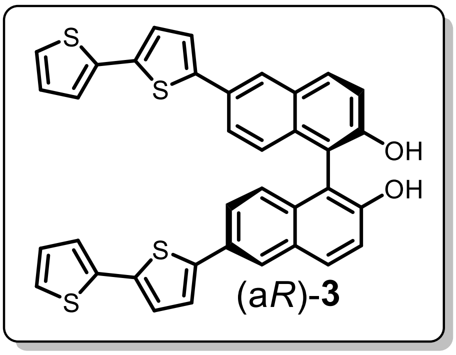

(*R*)-6,6'-Di([2,2'-bithiophen]-5-yl)-[1,1'-binaphthalene]-2,2'-diol ((a*R*)-3). (*R*)-6,6'-Dibromo-[1,1'-binaphthalene]-2,2'-diol ((a*R*)-**2**) (147 mg, 0.33 mmol), 2,2'-bithiophene-5-boronic acid pinacol ester (482 mg, 1.65 mmol), potassium carbonate 2 M aqueous solution (3.0 mL, 6.00 mmol) and 1,4-dioxane (10 mL) were mixed together. The solution was degassed by bubbling nitrogen for 30 minutes, then $PdCl_2(PPh_3)_2$ (18.3 mg, 0.026 mmol) was added. The resulting mixture was refluxed (oil bath) under stirring for 48 h, then it was cooled to room temperature, hydrolyzed with a saturated ammonium chloride solution (50 mL) and extracted with CH_2Cl_2 (3×50 mL). The combined organic phases were washed with brine (3×50 mL), dried over anhydrous Na_2SO_4 and the solvent was removed under vacuum. The crude product was purified through column chromatography (SiO_2, *n*-hexane/ethyl acetate 6:4) to give (*R*)-6,6'-di([2,2'-bithiophen]-5-yl)-[1,1'-binaphthalene]-2,2'-diol ((a*R*)-**3**) (59.0 mg, 0.096 mmol, yield 29%) as yellow solid. Analytical data are in agreement with those previously reported in the literature.[S2] **^{1}H NMR** (400 MHz, $CDCl_3$), δ (ppm): 8.09 (d, *J* = 1.9 Hz, 2H), 8.00 (d, *J* = 8.9 Hz, 2H), 7.57 (dd, *J* = 8.9, 1.9 Hz, 2H), 7.41 (d, *J* = 8.9 Hz, 2H), 7.28 (d, *J* = 3.8 Hz, 2H), 7.24 – 7.21 (m, 4H), 7.19 – 7.16 (m, 4H), 7.03 (dd, *J* = 5.1, 3.8 Hz, 2H), 5.16 (s,

2H). **^{13}C{^{1}H} NMR** (100 MHz, $CDCl_3$), δ (ppm): 153.1, 142.9, 137.5, 136.9, 132.9, 131.8, 130.1, 129.8, 128.0, 125.8, 125.1, 124.9, 124.7, 124.6, 124.1, 123.8, 118.8, 111.1. **M.p.**: 257-261 °C. **Anal.** calcd for $C_{36}H_{22}O_2S_4$: C, 70.33; H, 3.61; S, 20.86; found: C, 70.43; H, 3.69; S, 20.85.

Synthesis of methylene-bridged BINOL derivatives 4–6

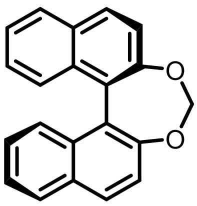

(a*R*)-**4**

(*R*)-Dinaphtho[2,1-*d*:1',2'-*f*][1,3]dioxepine ((a*R*)-4). (*R*)-[1,1'-binaphthalene]-2,2'-diol ((a*R*)-**1**) (501 mg, 1.75 mmol), potassium carbonate (14.5 g, 10.5 mmol) and acetonitrile (10 mL) were mixed together, then diiodomethane (423 µL, 5.25 mmol) was added dropwise. The reaction mixture was refluxed (oil bath) under stirring for 24 h, then it was cooled at room temperature, filtered and concentrated under vacuum. The crude product was purified through column chromatography (SiO_2, *n*-hexane → *n*-hexane/CH_2Cl_2 1:1) to give (*R*)-dinaphtho[2,1-*d*:1',2'-*f*][1,3]dioxepine ((a*R*)-**4**) (412 mg, 1.38 mmol, yield 79%) as a white solid. Analytical data are in agreement with those previously reported in the literature.[S3] **^{1}H NMR** (400 MHz, $CDCl_3$), δ (ppm): 7.99 (d, *J* = 8.7 Hz, 2H); 7.96 – 7.93 (m, 2H); 7.54 – 7.51 (m, 2H); 7.49 (d, *J* = 8.7 Hz, 2H); 7.48 – 7.43 (m, 2H); 7.33 – 7.29 (m, 2H); 5.71 (s, 2H). **^{13}C{^{1}H} NMR** (100 MHz, $CDCl_3$), δ (ppm): 151.4; 132.3; 131.9; 130.5; 128.5; 127.0; 126.2; 126.1; 125.1; 121.1; 103.3.

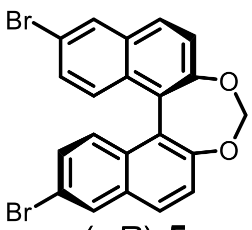

(a*R*)-**5**

(*R*)-9,14-Dibromodinaphtho[2,1-*d*:1',2'-*f*][1,3]dioxepine ((a*R*)-5). (*R*)-6,6'-Dibromo-[1,1'-binaphthalene]-2,2'-diol ((a*R*)-**2**) (560 mg, 1.26 mmol), potassium carbonate (10.45 g, 7.56 mmol) and acetone (100 mL) were mixed together, then diiodomethane (305 µL, 3.78 mmol) was added dropwise. The reaction mixture was refluxed (oil bath) under stirring for 24 h, then it was cooled at room temperature, filtered and concentrated under vacuum. The residue was dissolved in H_2O (100 mL) and extracted with CH_2Cl_2 (3×100 mL), then the organic phase was washed with brine (3×100 mL), dried over anhydrous Na_2SO_4 and the solvent was removed under vacuum. The crude product was purified through column chromatography (SiO_2, *n*-hexane/CH_2Cl_2 6:4) to give (*R*)-9,14-dibromodinaphtho[2,1-*d*:1',2'-*f*][1,3]dioxepine ((a*R*)-**5**) (178 mg, 0.39 mmol, yield 31%) as a white solid. Analytical data are in agreement with those previously reported in the literature.[S4] **^{1}H NMR** (400 MHz, $CDCl_3$), δ (ppm): 8.11 (d, *J* = 2.0 Hz, 2H); 7.90 (d, *J* = 8.7 Hz, 2H); 7.50 (d, *J* = 8.7 Hz, 2H); 7.39 (dd, *J* = 9.1, 2.0 Hz, 2H); 7.31 (d, *J* = 9.1 Hz, 2H); 5.69 (s, 2H). **^{13}C{^{1}H} NMR** (100 MHz, $CDCl_3$), δ (ppm): 151.7; 133.1; 130.7; 130.6; 129.8; 128.5; 126.0; 122.4; 119.3; 103.3.

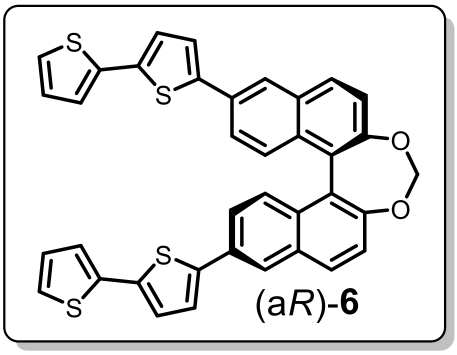

(*R*)-9,14-Di([2,2′-bithiophen]-5-yl)dinaphtho[2,1-*d*:1′,2′-*f*][1,3]dioxepine ((a*R*)-6). (*R*)-9,14-Dibromodinaphtho[2,1-*d*:1′,2′-*f*][1,3]dioxepine ((a*R*)-**5**) (151 mg, 0.33 mmol), 2,2′-bithiophene-5-boronic acid pinacol ester (482 mg, 1.65 mmol), potassium carbonate 2 M aqueous solution (3.0 mL, 6.00 mmol) and 1,4-dioxane (10 mL) were mixed together. The solution was degassed by bubbling nitrogen for 30 minutes, then $PdCl_2(PPh_3)_2$ (18.3 mg, 0.026 mmol) was added. The resulting mixture was refluxed (oil bath) under stirring for 48 h, then it was cooled to room temperature, hydrolyzed with a saturated ammonium chloride solution (50 mL) and extracted with CH_2Cl_2 (3×50 mL). The combined organic phases were washed with brine (3×50 mL), dried over anhydrous Na_2SO_4 and the solvent was removed under vacuum. The crude product was purified through column chromatography (SiO_2, *n*-hexane/CH_2Cl_2 9:1 → CH_2Cl_2) to give (*R*)-9,14-di([2,2′- bithiophen]-5-yl)dinaphtho[2,1-*d*:1′,2′-*f*][1,3]dioxepine ((a*R*)-**6**) (132 mg, 0.21 mmol, yield 64%) as yellow solid. **^{1}H NMR** (400 MHz, $CDCl_3$), δ (ppm): 8.14 (d, *J* = 1.7 Hz, 2H), 8.00 (d, *J* = 8.7 Hz, 2H), 7.61 – 7.49 (m, 6H), 7.35 (d, *J* = 3.8 Hz, 2H), 7.25 – 7.23 (m, 4H), 7.20 (d, *J* = 3.8 Hz, 2H), 7.06 (dd, *J* = 4.9, 3.8 Hz, 2H), 5.72 (s, 2H). **^{13}C{^{1}H} NMR** (100 MHz, $CDCl_3$), δ (ppm): 151.6, 142.8, 137.5, 137.2, 132.2, 131.5, 130.9, 130.6, 128.1, 127.7, 126.2, 124.9, 124.7, 124.5, 124.4, 124.4, 123.9, 121.9, 103.4. **M.p.**: 236-240 °C. **Anal.** calcd for $C_{37}H_{22}O_2S_4$: C, 70.90; H, 3.54; S, 20.46; found: C, 71.03; H, 3.65; S, 20.44.

Synthesis of the achiral π-conjugated dye 7

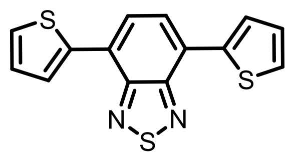

7

4,7-Di(thiophen-2-yl)benzo[*c*][1,2,5]thiadiazole (7). 4,7-Dibromobenzo[*c*][1,2,5]thiadiazole (506 mg, 1.72 mmol), thiophen-2-ylboronic acid (660 mg, 5.16 mmol), potassium carbonate 2 M aqueous solution (10 mL, 20.0 mmol) and 1,4-dioxane (30 mL) were mixed together. The solution was degassed by bubbling nitrogen for 30 minutes, then $PdCl_2(PPh_3)_2$ (60.4 mg, 0.086 mmol) was added. The resulting mixture was refluxed (oil bath) under stirring for 24 h, then it was cooled to room temperature, hydrolyzed with water (50 mL) and extracted with CH_2Cl_2 (3×50 mL). The combined organic phases were washed with brine (3×50 mL), dried over anhydrous Na_2SO_4 and the solvent was removed under vacuum. The crude product was purified by recrystallization from ethanol (3 times) to give 4,7-di(thiophen-2-yl)benzo[*c*][1,2,5] thiadiazole (**7**) (322 mg, 1.07 mmol, yield 62%) as orange solid. Analytical data are in agreement with those previously reported in the literature.[S5] **^{1}H NMR** (400 MHz, $CDCl_3$), δ (ppm): 8.12 (dd, *J* = 3.7, 1.1 Hz, 2H); 7.89 (s, 2H); 7.46 (dd, *J* = 5.1, 1.1 Hz, 2H); 7.22 (dd, *J* = 5.1, 3.7 Hz, 2H). **^{13}C{^{1}H} NMR** (100 MHz, $CDCl_3$), δ (ppm): 152.6; 139.4; 128.1; 127.6; 126.8; 126.0; 125.7.

Experimental Section: Characterization

Characterization in solution

Absorbance. UV-Vis absorbance measurements of BINOL derivatives in solution were performed at room temperature with a Jasco V-650 spectrophotometer. Stock solutions of each dye in acetonitrile (for compounds (a*R*)-**1–5**) or CH_2Cl_2 (for compound (a*R*)-**6**) were 10^{-2} M, while working solutions were 10^{-5} M. Molar extinction coefficients ε were calculated at one or more wavelengths of maximum absorbance, by using five working solutions in the same solvent (acetonitrile for compounds (a*R*)-**1–5**, CH_2Cl_2 for compound (a*R*)-**6**) at different concentrations, between $1.0 \cdot 10^{-6}$ M and $5.0 \cdot 10^{-6}$ M.

Electronic circular dichroism. Electronic circular dichroism (ECD) measurements of BINOL derivatives in solution were performed at room temperature using a Jasco J-1500 Circular Dichroism Spectrophotometer. Stock solutions of each dye in acetonitrile (for compounds (a*R*)-**1–5**) or CH_2Cl_2 (for compound (a*R*)-**6**) were 10^{-2} M, while working solutions were 10^{-5} M. The corresponding dissymmetry factor g_{abs} spectra were calculated as "Not normalized ECD spectrum/UV-Vis absorbance spectrum", *i.e.*, as g_{abs} = (Not normalized Ellipticity (mdeg)/32980)/Absorbance.

Characterization in thin films

Thin films preparation. Thin films of BINOL derivatives (a*R*)-**1–5** were prepared by spin coating technique, depositing 100 µL of a $2.0 \cdot 10^{-2}$ M CH_2Cl_2 solution of the chiral dye on circular quartz plates with a WS-650MZ-23NPPB (Laurell Technologies Corp., North Wales, PA, USA) spin-coater, under the following experimental conditions: duration 30 s; spinning rate 1000 rpm; acceleration 1000 rpm s^{-1}. Thin films of methylene-bridged BINOL derivative (a*R*)-**6** were prepared by drop casting technique, depositing 100 µL of a $3.0 \cdot 10^{-3}$ M CH_2Cl_2 solution of the chiral dye on circular quartz plates, followed by slow evaporation in an atmosphere saturated with CH_2Cl_2 vapours. Solvent annealing was carried out on selected samples by keeping them in a closed chamber saturated with CH_2Cl_2 vapours for a given time. Thermal annealing was carried out on selected samples by keeping them in an oven at 120 °C for a given time.

Thin films of blends of the achiral π-conjugated dye **7** with methylene-bridged BINOL chiral inducer (a*R*)-**6** were prepared by spin coating technique, depositing 150 µL of a 0.1 M $CHCl_3$ solution of **7** with different amounts (0.5 – 5.0 mol%) of (a*R*)-**6** on circular quartz plates with a WS-650MZ-23NPPB (Laurell Technologies Corp., North Wales, PA, USA) spin-coater, under the following experimental conditions: duration 30 s; spinning rate 1000 rpm; acceleration 1000 rpm s^{-1}.

Absorbance. UV-Vis absorbance measurements of both BINOL derivatives and blends in thin films were recorded at room temperature using a Jasco V-650 spectrophotometer, placing samples orthogonally with respect to the instrumental optical path.

Photoluminescence. Photoluminescence measurements of blends in thin films were recorded at room temperature with a home-made spectrofluoropolarimeter,[S6] using a 365 nm LED excitation source arranged on the same direction of collection of the emitted light (ideally, 0° geometry with respect to the detector).

Electronic circular dichroism. ECD measurements of both BINOL derivatives and blends in thin films were performed at room temperature using a Jasco J-1500 Circular Dichroism Spectrophotometer. In all cases, at least 3 independent thin film samples were prepared and subjected to full ECD analysis. First, invariance of ECD upon sample rotation was testified, by rotating thin film sample by 90°, 180° and 270° around the optical axis: in no cases were found variations. Then, for each sample two ECD measurements were recorded: one with the organic layer facing the light source (*front face*) and one with the organic layer facing the detector (*back face*). In the presence of non-negligible differences between the two spectral profiles, the semi-sum and semi-difference of front and back ECD spectra were calculated to obtain intrinsic CDiso and non-reciprocal LDLB contributions, according to the following equations:

$$\mathrm{CDiso} = \tfrac{1}{2} \times (\mathrm{ECD}_{\mathrm{front}} + \mathrm{ECD}_{\mathrm{back}}) \qquad \text{(S1)}$$

$$\mathrm{LDLB} = \tfrac{1}{2} \times (\mathrm{ECD}_{\mathrm{front}} - \mathrm{ECD}_{\mathrm{back}}) \qquad \text{(S2)}$$

For each measured ECD spectrum, the corresponding dissymmetry factor g_{abs} spectrum was calculated as "Not normalized ECD spectrum/UV-Vis absorbance spectrum", *i.e.*, as g_{abs} = (Not normalized Ellipticity (mdeg)/32980)/Absorbance.

Circularly polarized luminescence. Circularly polarized luminescence (CPL) measurements of blends in thin films were recorded at room temperature with a home-made spectrofluoropolarimeter,[S6] using a 365 nm LED excitation source arranged on the same direction of collection of the emitted light (ideally, 0° geometry with respect to the detector). The light emitted by thin film samples passed through a photoelastic modulator (PEM), which converts alternatively (50 KHz) right-handed and left-handed circularly polarized light into linearly polarized light. A component of the linearly polarized light is then selected by a linear polarizer (at 45° with respect to PEM easy axis), rendered monochromatic by the emission monochromator (diffraction grating, Jasco CT-10, 1200 grooves/mm blazed at 500 nm) and sent to the detector (photomultiplier tube, PMT, Hamamatsu R376). A lock-in amplifier, in phase with the PEM, extracts the left and right polarized component of the emission and therefore the CPL signal (CPL = $I_L - I_R$). At the same time, the instrument acquires the total luminescence signal ($I = I_L + I_R$). The two signals are referred to as alternating current (AC) and direct current (DC). respectively. A programmable electronic prototyping platform (Arduino UNO) checked the step motor driving the emission monochromator and digitalized the analog signals (both AC and DC) from the instrument. These data are transferred to a Microsoft Excel spreadsheet through a data acquisition macro (PLX-DAQ) and the CPL and total emission spectra are plotted in real time. The following acquisition parameters were employed: slit-width 5 nm, scan-speed 1 nm/sec, integration time 1 sec, PMT HV 600 V, PEM modulation 50 KHz. In all cases, at least 3 independent thin film samples were prepared and subjected to full CPL analysis. First, invariance of CPL amplitudes upon sample rotation was testified, through successive 90° increments around the optical axis: in no cases were found significant variations. Then, for each sample two CPL measurements were recorded: one with the organic layer facing the light source (*front face*) and one with the organic layer facing the detector (*back face*). Residual fluorescence linear polarization was also measured, using lock-in detection at double frequency (100 KHz) with respect to the PEM modulation frequency; in all cases, it was found to be negligible.

For each measured CPL spectrum, the corresponding luminescence dissymmetry factor g_{lum} spectrum was calculated as $g_{lum} = 2 \times (I_L - I_R)/(I_L + I_R)$.

Computational Section

All calculations were run with Gaussian16, rev. C.01,[S7] with default grids and convergence criteria. All structures were built in GaussView and optimized at B3LYP-D3BJ/6-311+G(d,p) level, including SMD solvent model for acetonitrile. For compounds (a*R*)-**1**, (a*R*)-**2**, (a*R*)-**4** and (a*R*)-**5**, a single conformer with C_2 symmetry was considered, with each O-H directed toward the other naphthalene ring. For compounds (a*R*)-**3** and (a*R*)-**6**, the input structures were generated systematically varying the torsions the C6-thiophene and thiophene-thiophene bonds, yielding 8 distinct conformers which were optimized at the same level as above. Their structures and relative energies are listed in Figures S27-S28. To simulate absorption and ECD spectra, TD-DFT calculations were run using two functionals (B3LYP and CAM-B3LYP), def2-TZVP basis set, and IEF-PCM solvent model for acetonitrile or dichloromethane. The two functionals led to consistent results, with CAM-B3LYP reproducing better the experimental spectra. Model chromophores (2-naphthol and 6-bromo-2-naphthol) were similarly analysed at CAM-B3LYP/def2-TZVP//B3LYP-D3BJ/6-311+G(d,p) level. Weighted-average spectra were obtained using Boltzmann populations at 300K, estimated from internal energies, and plotted using SpecDis v. 1.71 (T. Bruhn, A. Schaumlöffel, Y. Hemberger, G. Pescitelli, SpecDis version 1.71, Berlin, Germany, 2017, http://specdis-software.jimdo.com). Plotting parameters are reported in the main text. Hole-electron plots were generated using MultiWfn v3.8.[S8]

Supplementary Figures

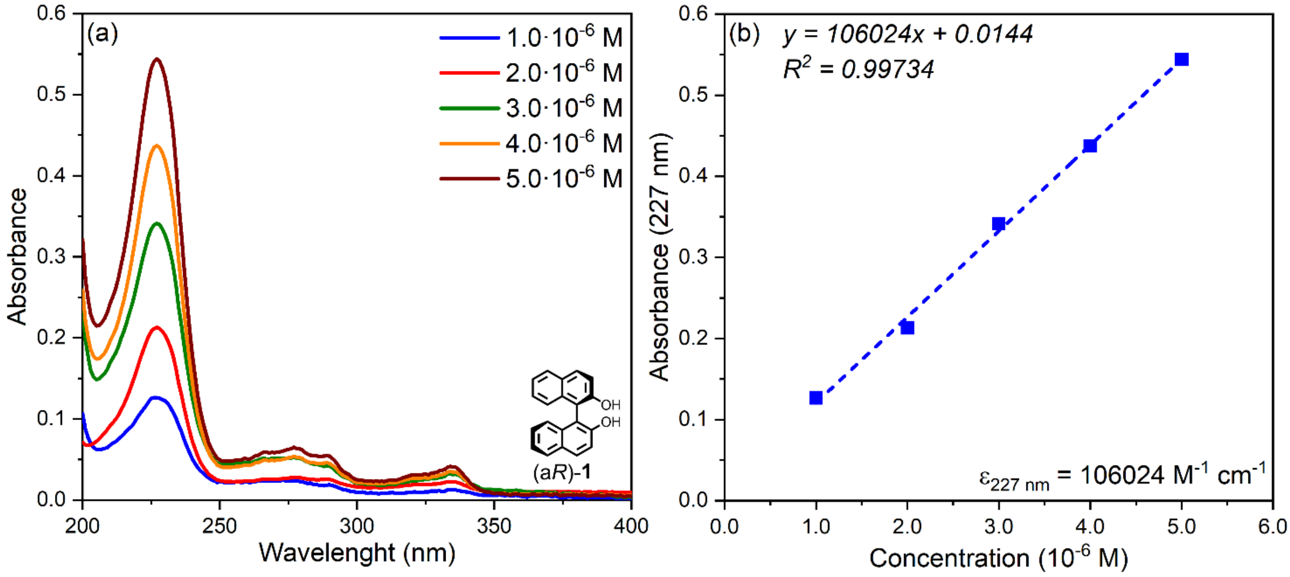

Figure S1.

Determination of molar extinction coefficients ε of BINOL (a*R*)-**1** in acetonitrile solution: (a) UV-Vis absorbance spectra at different concentrations (from $1.0 \cdot 10^{-6}$ M to $5.0 \cdot 10^{-6}$ M); (b) absorbance at 227 nm *vs.* concentration plot.

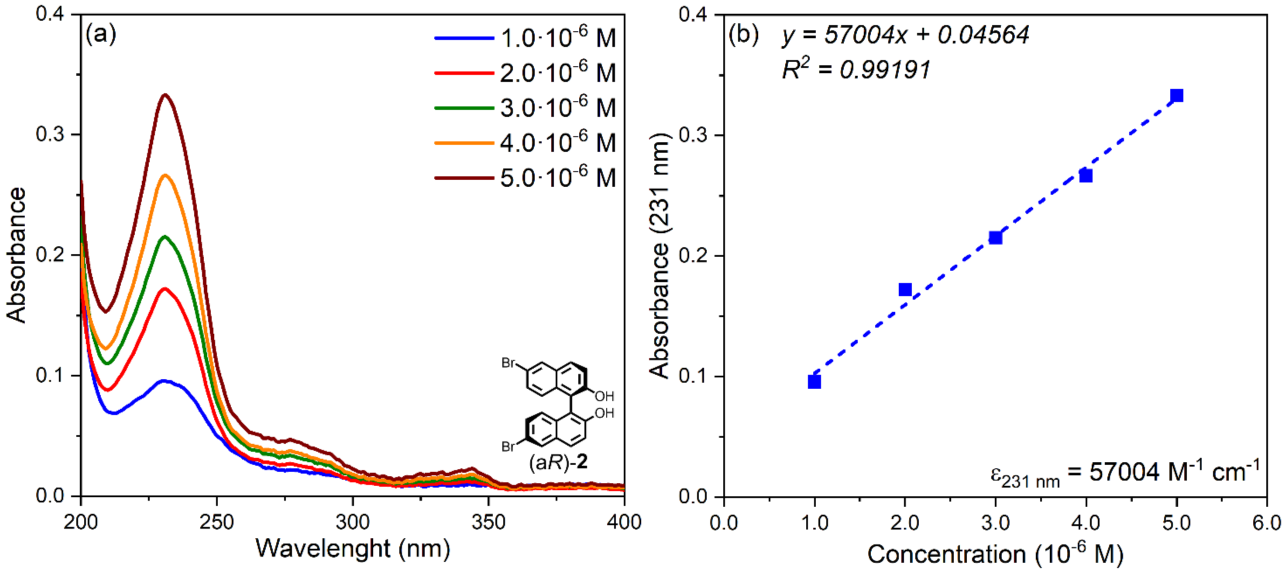

Figure S2.

Determination of molar extinction coefficients ε of open BINOL derivative (a*R*)-**2** in acetonitrile solution: (a) UV-Vis absorbance spectra at different concentrations (from 1.0 · 10^{-6} M to 5.0 · 10^{-6} M); (b) absorbance at 231 nm *vs.* concentration plot.

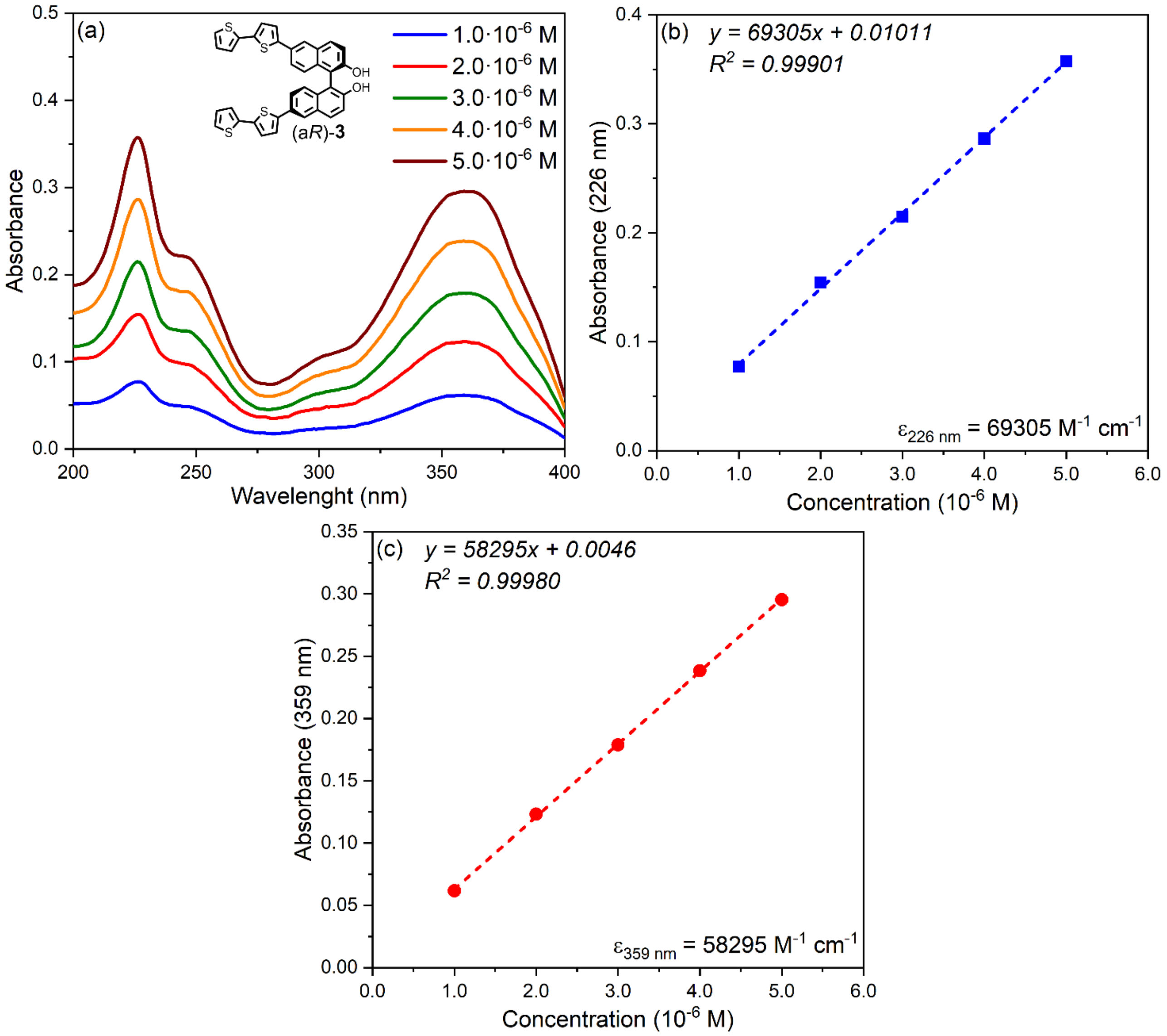

Figure S3.

Determination of molar extinction coefficients ε of open BINOL derivative (a*R*)-**3** in acetonitrile solution: (a) UV-Vis absorbance spectra at different concentrations (from $1.0 \cdot 10^{-6}$ M to $5.0 \cdot 10^{-6}$ M); (b) absorbance at 226 nm *vs.* concentration plot; (c) absorbance at 359 nm *vs.* concentration plot.

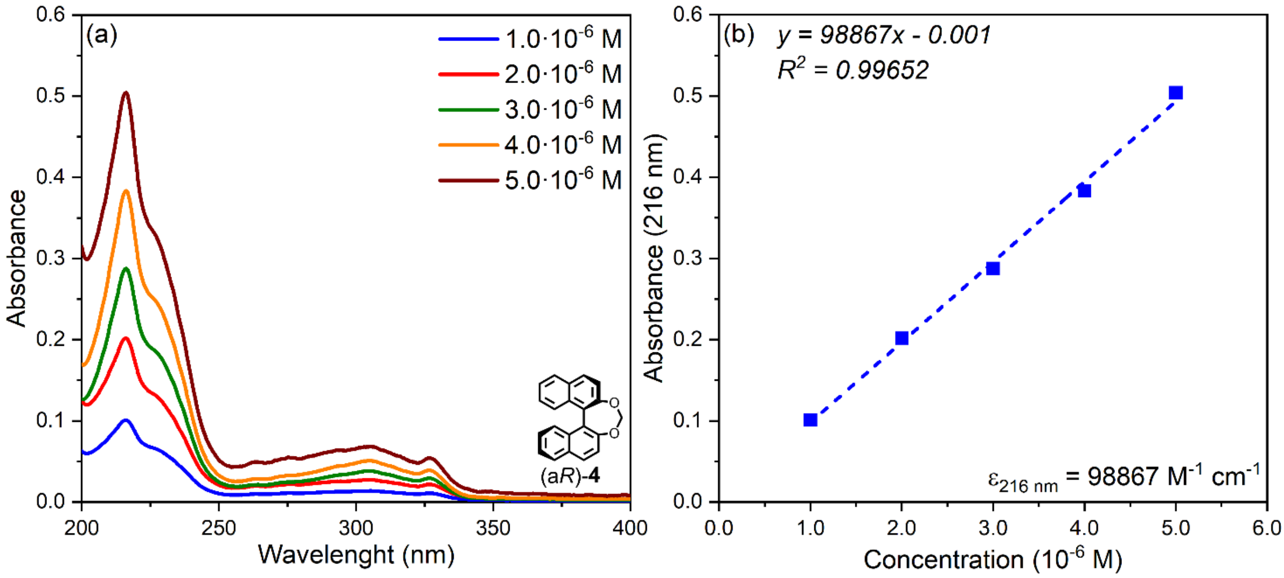

Figure S4.

Determination of molar extinction coefficients ε of methylene-bridged BINOL derivative (a*R*)-**4** in acetonitrile solution: (a) UV-Vis absorbance spectra at different concentrations (from $1.0 \cdot 10^{-6}$ M to $5.0 \cdot 10^{-6}$ M); (b) absorbance at 216 nm *vs.* concentration plot.

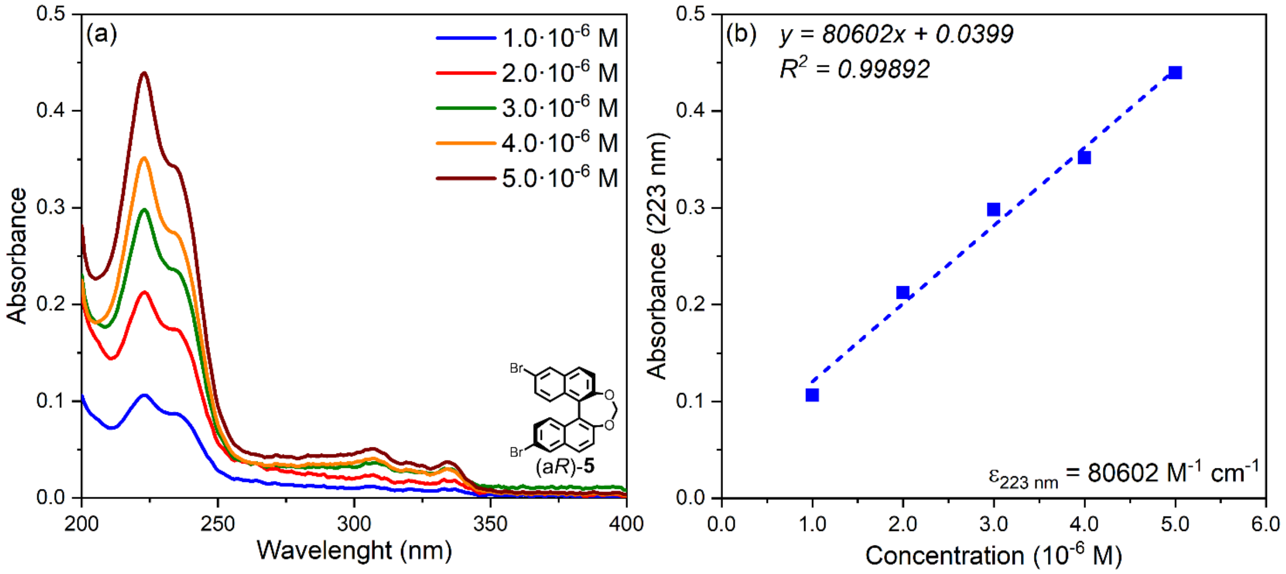

Figure S5.

Determination of molar extinction coefficients ε of methylene-bridged BINOL derivative (a*R*)-**5** in acetonitrile solution: (a) UV-Vis absorbance spectra at different concentrations (from $1.0 \cdot 10^{-6}$ M to $5.0 \cdot 10^{-6}$ M); (b) absorbance at 223 nm *vs.* concentration plot.

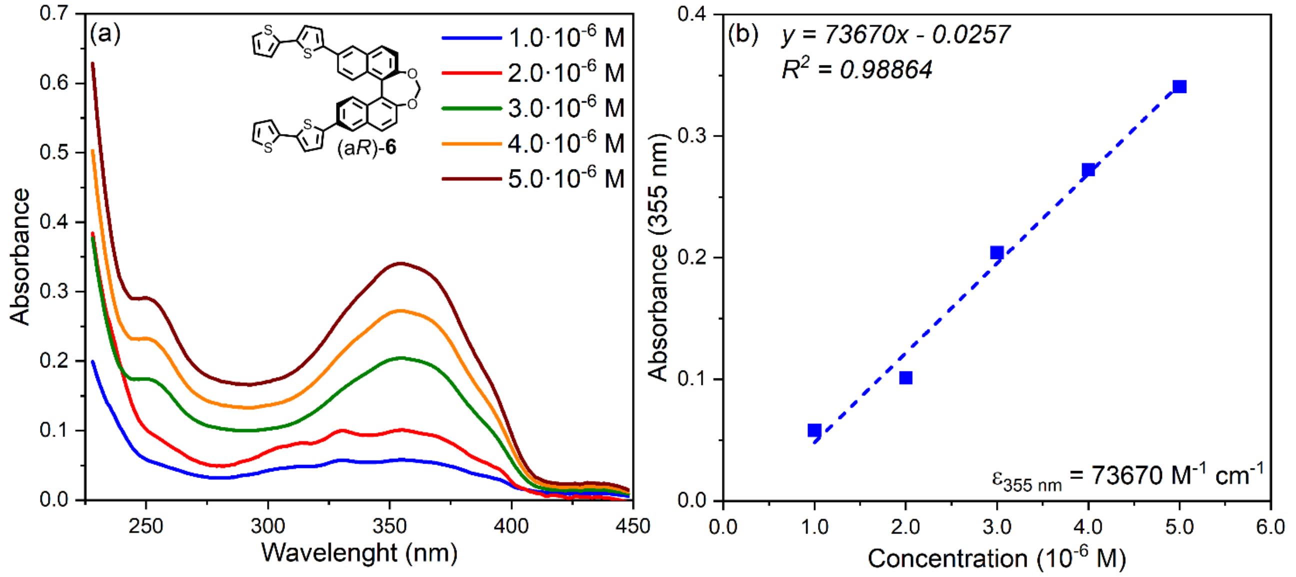

Figure S6.

Determination of molar extinction coefficients ε of methylene-bridged BINOL derivative (a*R*)-**6** in CH_2Cl_2 solution: (a) UV-Vis absorbance spectra at different concentrations (from $1.0 \cdot 10^{-6}$ M to $5.0 \cdot 10^{-6}$ M); (b) absorbance at 355 nm *vs.* concentration plot.

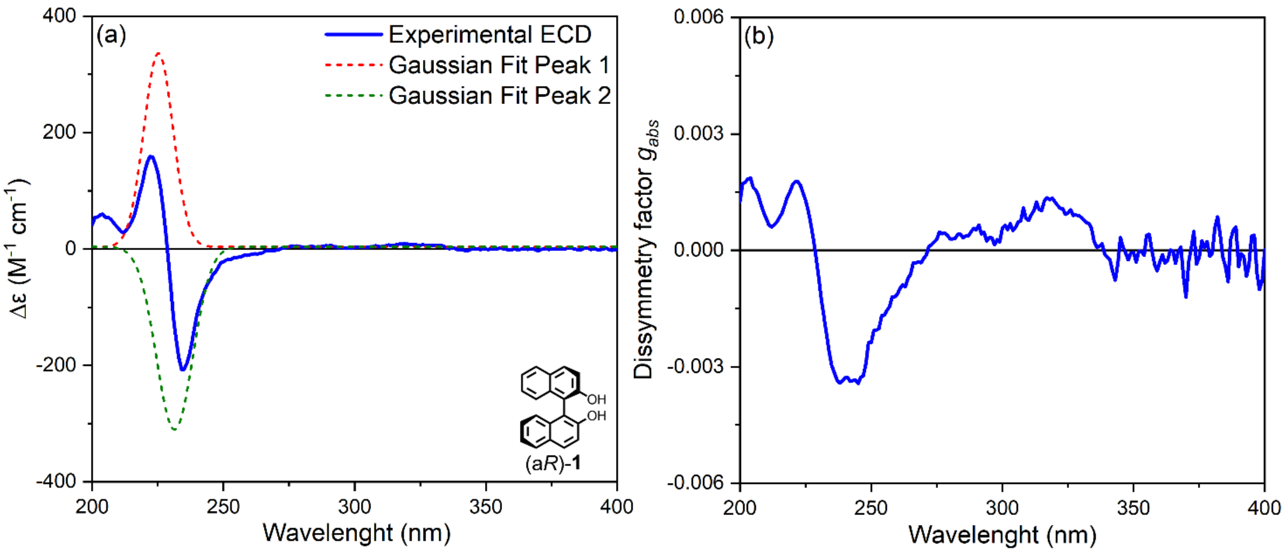

Figure S7.

(a) Multi-peak Gaussian fitting for the main couplet in the experimental ECD spectrum and (b) dissymmetry factor g_{abs} spectrum of BINOL (a*R*)-**1** in acetonitrile solution.

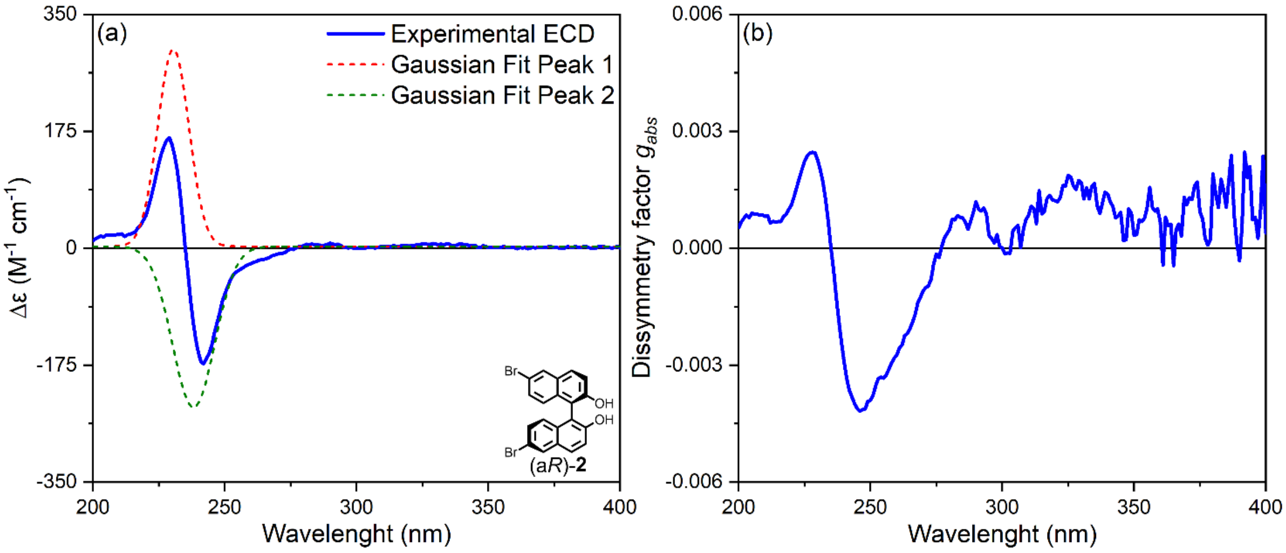

Figure S8.

(a) Multi-peak Gaussian fitting for the main couplet in the experimental ECD spectrum and (b) dissymmetry factor g_{abs} spectrum of open BINOL derivative (a*R*)-**2** in acetonitrile solution.

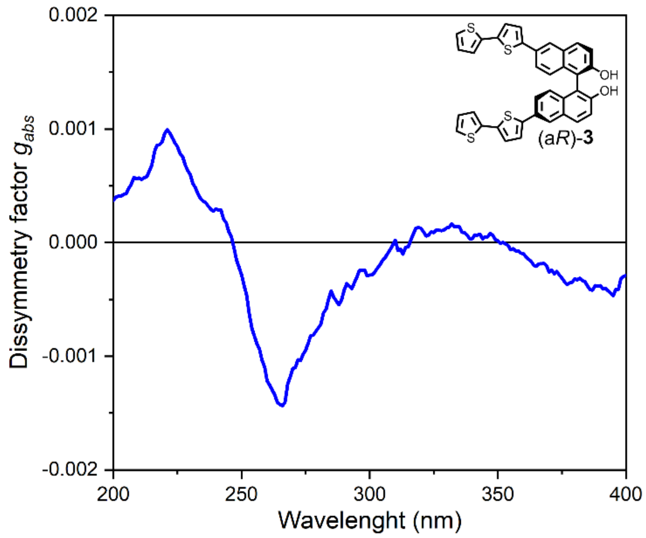

Figure S9.

Dissymmetry factor g_{abs} spectrum of open BINOL derivative (a*R*)-**3** in acetonitrile solution.

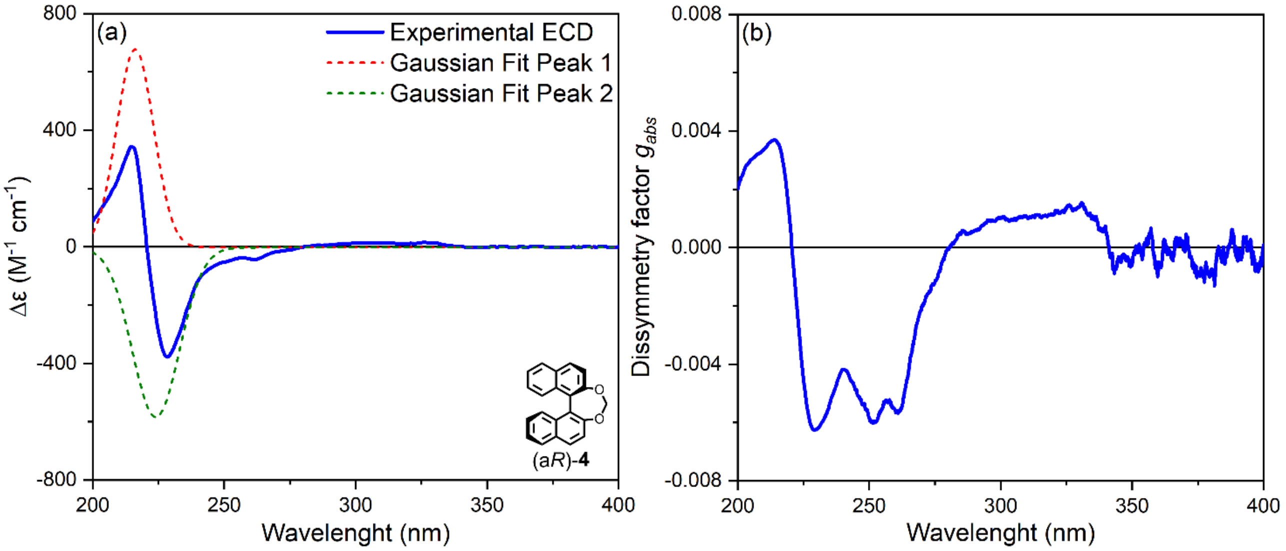

Figure S10.

(a) Multi-peak Gaussian fitting for the main couplet in the experimental ECD spectrum and (b) dissymmetry factor g_{abs} spectrum of methylene-bridged BINOL derivative (a*R*)-**4** in acetonitrile solution.

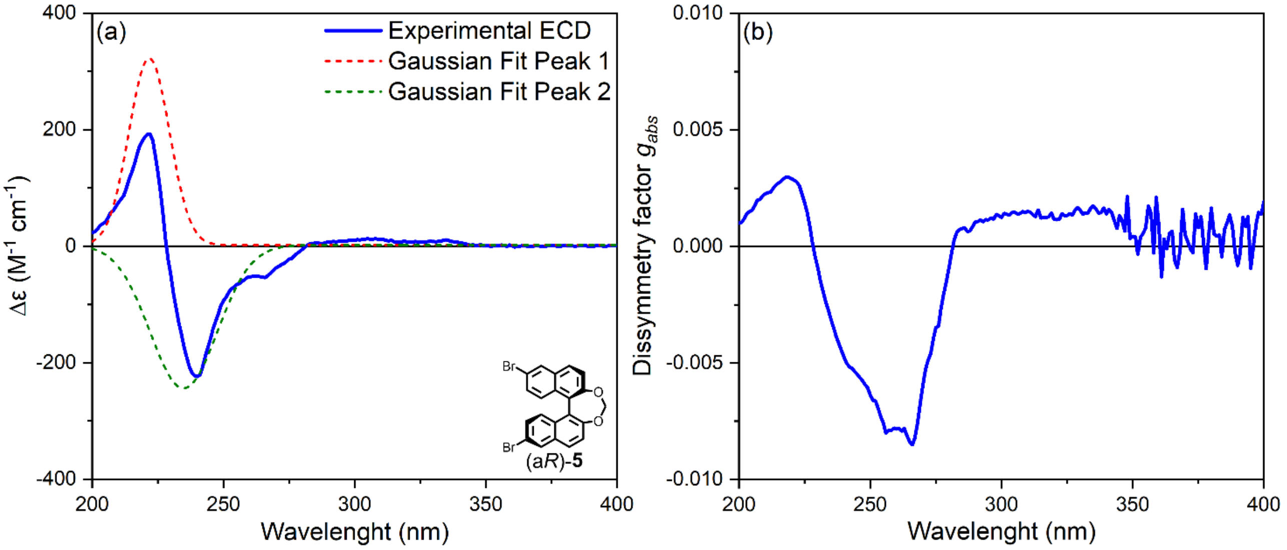

Figure S11.

(a) Multi-peak Gaussian fitting for the main couplet in the experimental ECD spectrum and (b) dissymmetry factor g_{abs} spectrum of methylene-bridged BINOL derivative (a*R*)-**5** in acetonitrile solution.

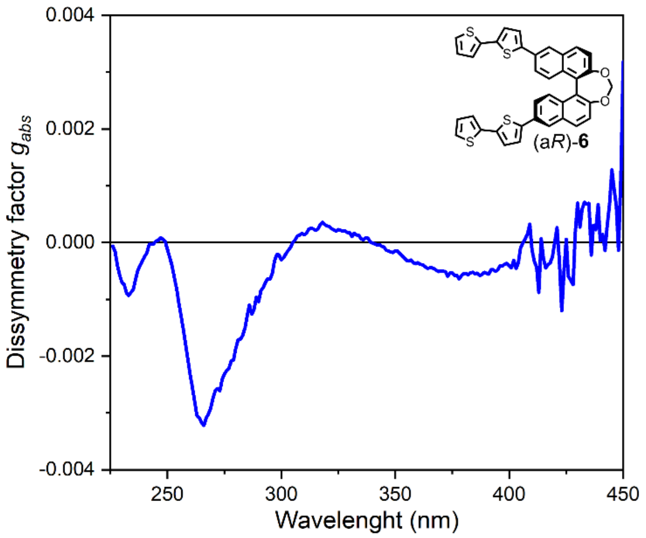

Figure S12.

Dissymmetry factor g_{abs} spectrum of methylene-bridged BINOL derivative (a*R*)-**6** in CH_2Cl_2 solution.

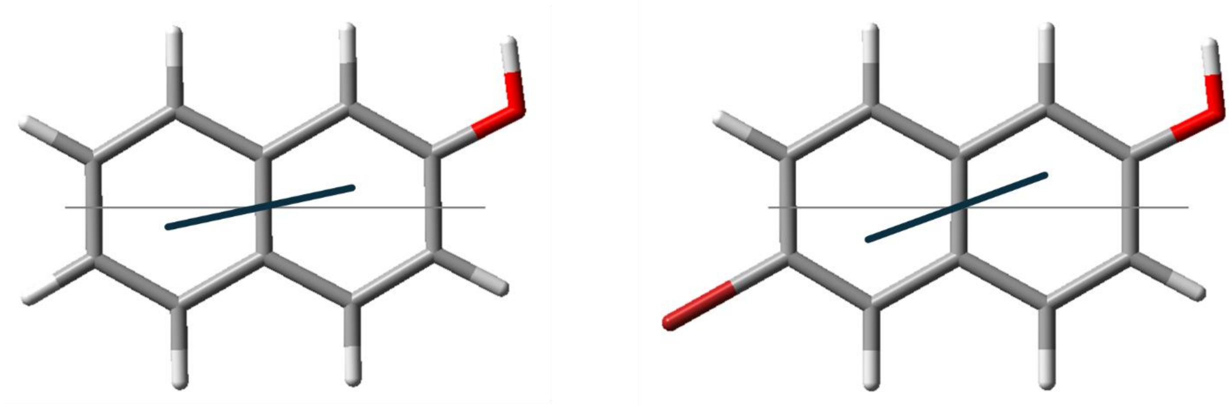

Figure S13.
Orientation of ^1B_b EDTM calculated for 2-naphthol (left) and 6-bromo-2-naphthol (right) at CAM-B3LYP/def2-TZVP//B3LYP-D3BJ/6-311+G(d,p) level. The angles with respect to the horizontal axis are 11.7° and 19.7°, respectively.

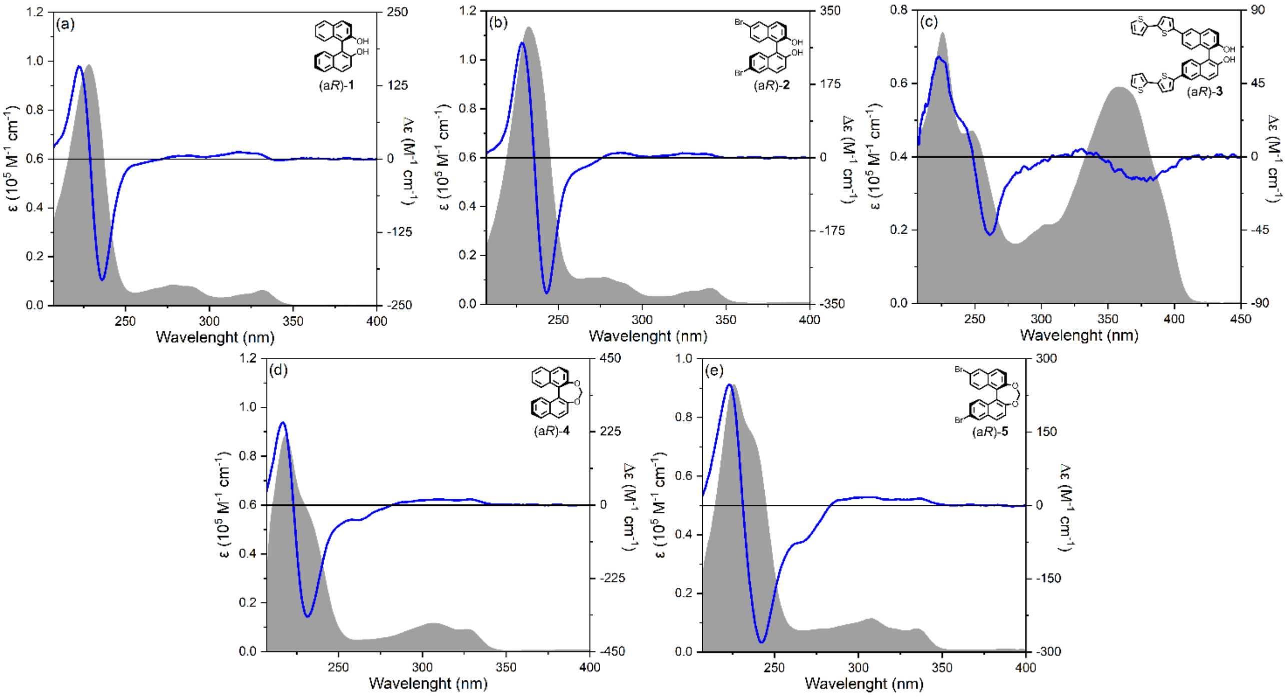

Figure S14.

UV-Vis absorbance (grey profile) and ECD (blue line) spectra in CH_2Cl_2 solution of: (a) (a*R*)-**1**; (b) (a*R*)-**2**; (c) (a*R*)-**3**; (d) (a*R*)-**4**; (e) (a*R*)-**5**. Cell length 0.01 cm; sample concentration 10^{-3} M.

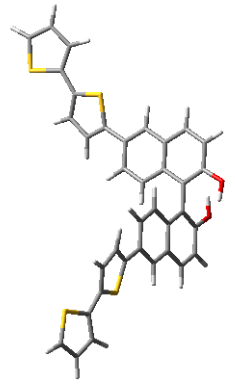<br>Absolute minimum	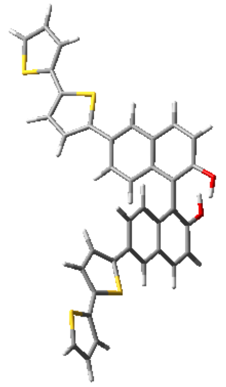<br>+0.01 kcal/mol
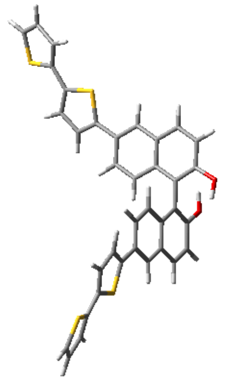<br>+0.11 kcal/mol	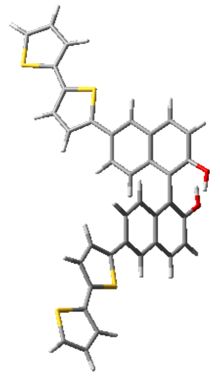<br>+0.13 kcal/mol
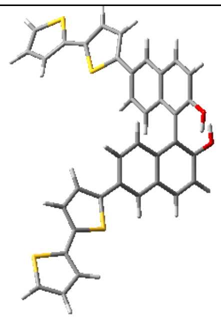<br>+0.37 kcal/mol	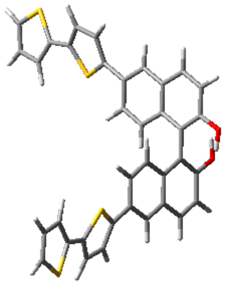<br>+0.50 kcal/mol
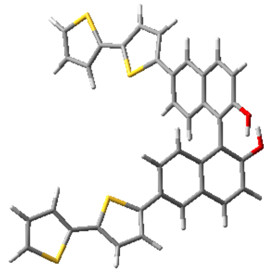<br>+0.51 kcal/mol	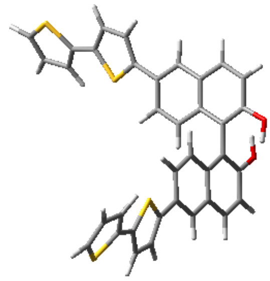<br>+0.63 kcal/mol

Figure S15.
DFT-optimized structures of (a*R*)-**3** at B3LYP-D3BJ/6-311+G(d,p) level, with relative internal energies.

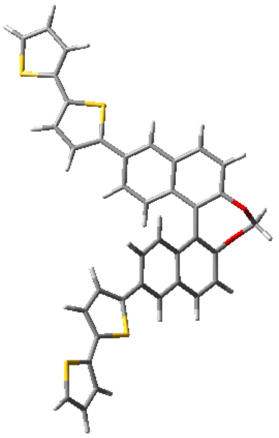

Absolute minimum

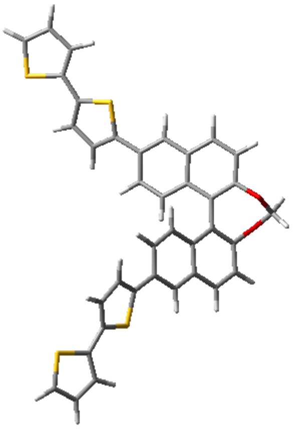

+0.02 kcal/mol

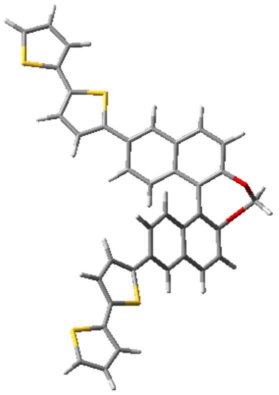

+0.10 kcal/mol

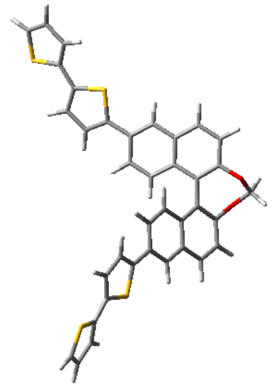

+0.17 kcal/mol

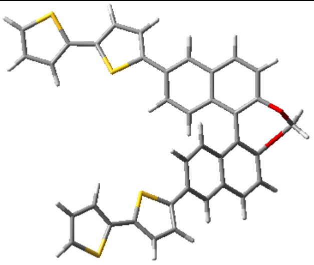

+0.57 kcal/mol

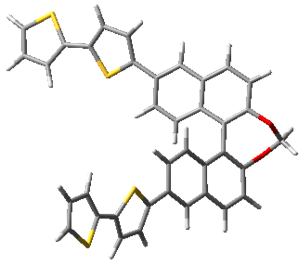

+0.61 kcal/mol

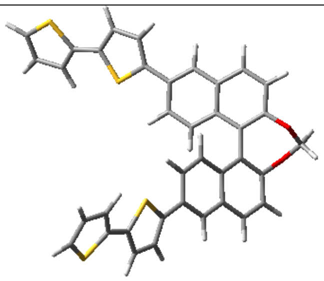

+0.66 kcal/mol

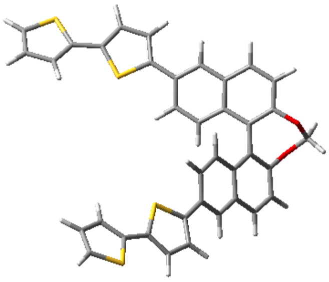

+0.66 kcal/mol

Figure S16.

DFT-optimized structures of (a*R*)-**6** at B3LYP-D3BJ/6-311+G(d,p) level, with relative internal energies.

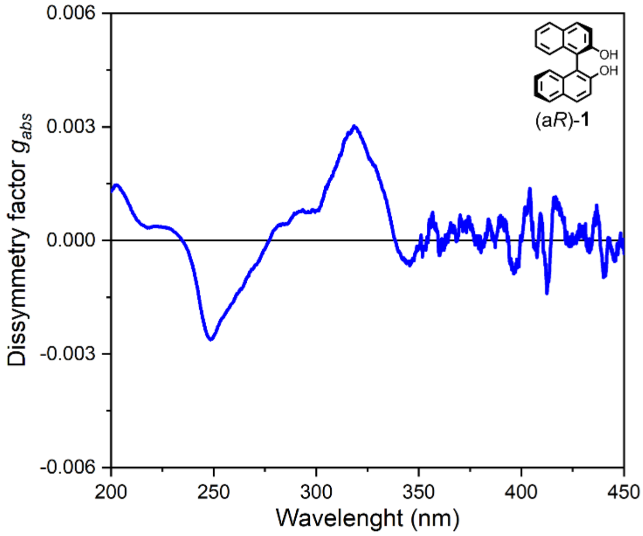

Figure S17.

Dissymmetry factor g_{abs} spectrum of as-cast thin film samples of BINOL (a*R*)-**1**, prepared by spin coating from a $2{\cdot}10^{-2}$ M CH_2Cl_2 solution.

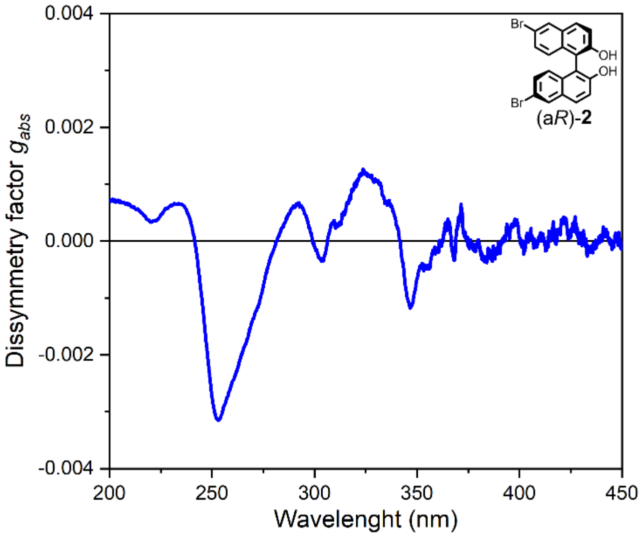

Figure S18.

Dissymmetry factor g_{abs} spectrum of as-cast thin film samples of open BINOL derivative (a*R*)-**2**, prepared by spin coating from a $2{\cdot}10^{-2}$ M CH_2Cl_2 solution.

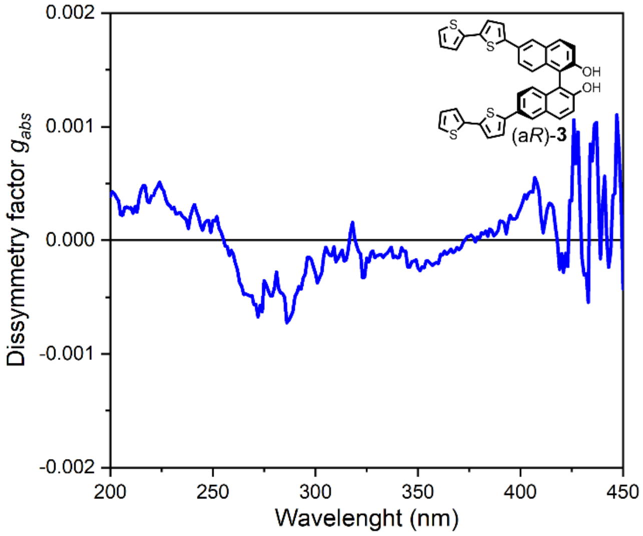

Figure S19.
Dissymmetry factor g_{abs} spectrum of as-cast thin film samples of open BINOL derivative (a*R*)-**3**, prepared by spin coating from a $2{\cdot}10^{-2}$ M CH_2Cl_2 solution.

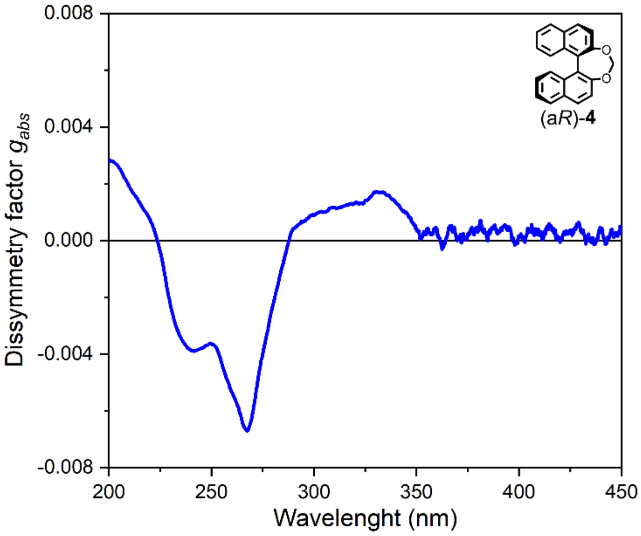

Figure S20.

Dissymmetry factor g_{abs} spectrum of as-cast thin film samples of methylene-bridged BINOL derivative (a*R*)-**4**, prepared by spin coating from a $2{\cdot}10^{-2}$ M CH_2Cl_2 solution.

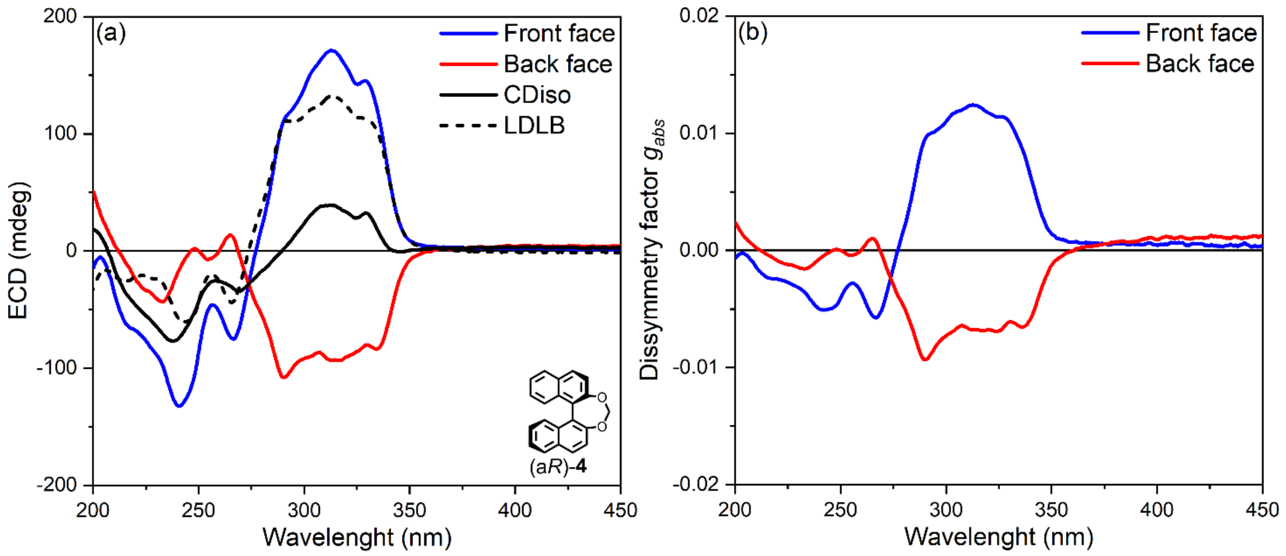

Figure S21.

(a) ECD and (b) dissymmetry factor g_{abs} spectra recorded for the front face (blue line) and the back face (red line) of thin film samples of methylene-bridged BINOL derivative (a*R*)-**4**, prepared by spin coating from a $2 \cdot 10^{-2}$ M CH_2Cl_2 solution, after 5 min of solvent annealing under CH_2Cl_2 vapours. For panel (a): black continuous line is the front-back semi-sum of ECD, *i.e.*, calculated CDiso contribution; black dashed line is the front-back semi-difference of ECD, *i.e.*, calculated LDLB contribution.

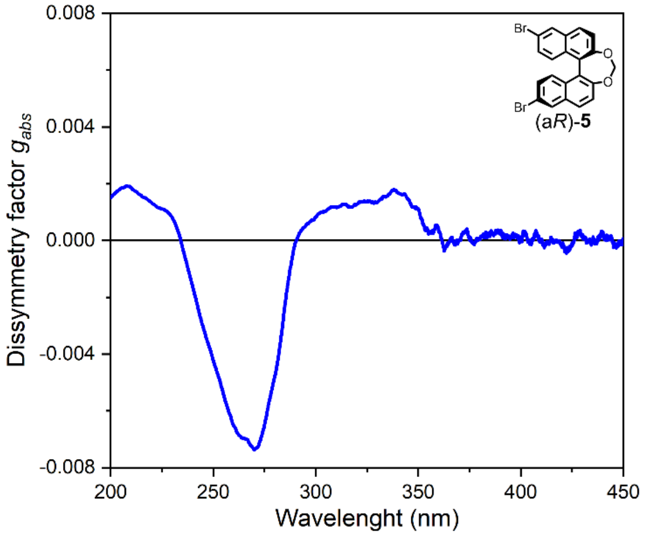

Figure S22.

Dissymmetry factor g_{abs} spectrum of as-cast thin film samples of methylene-bridged BINOL derivative (a*R*)-**5**, prepared by spin coating from a $2{\cdot}10^{-2}$ M CH_2Cl_2 solution.

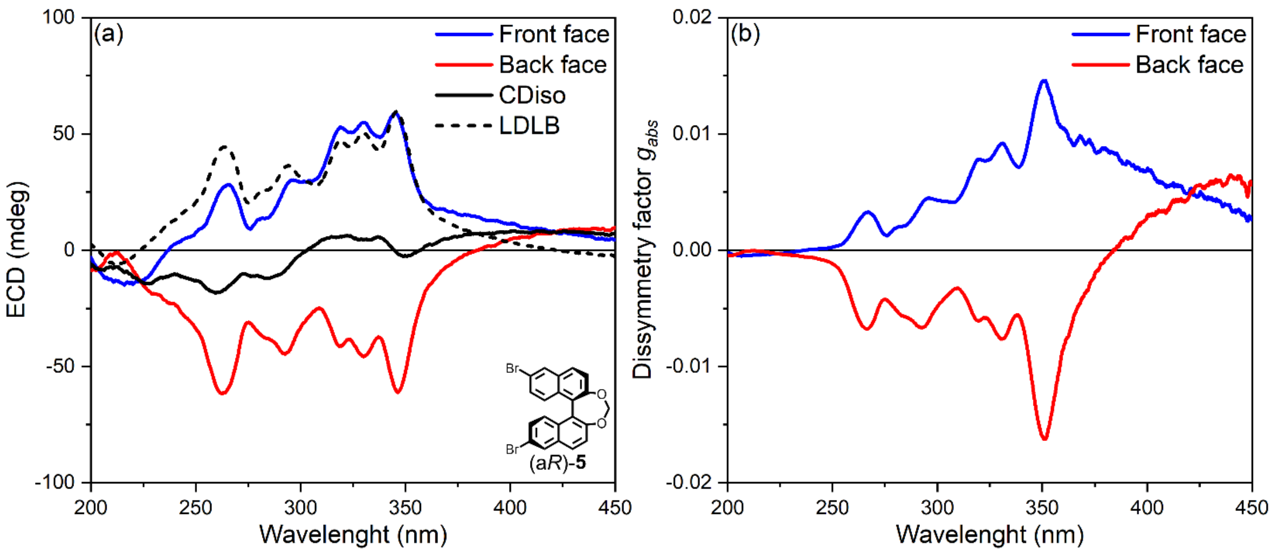

Figure S23.

(a) ECD and (b) dissymmetry factor g_{abs} spectra recorded for the front face (blue line) and the back face (red line) of thin film samples of methylene-bridged BINOL derivative (a*R*)-**5**, prepared by spin coating from a $2{\cdot}10^{-2}$ M CH_2Cl_2 solution, after 10 min of thermal annealing at 120 °C. For panel (a): black continuous line is the front-back semi-sum of ECD, *i.e.*, calculated CDiso contribution; black dashed line is the front-back semi-difference of ECD, *i.e.*, calculated LDLB contribution.

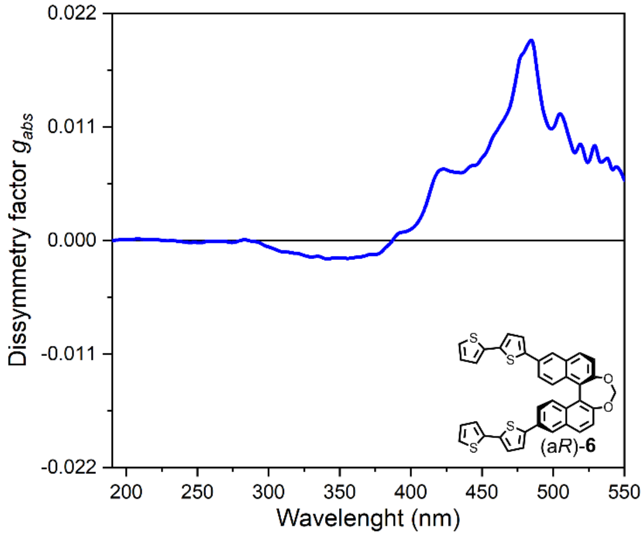

Figure S24.

Dissymmetry factor g_{abs} spectrum of as-cast thin film samples of methylene-bridged BINOL derivative (a*R*)-**6**, prepared by drop coating from a $3{\cdot}10^{-3}$ M CH_2Cl_2 solution.

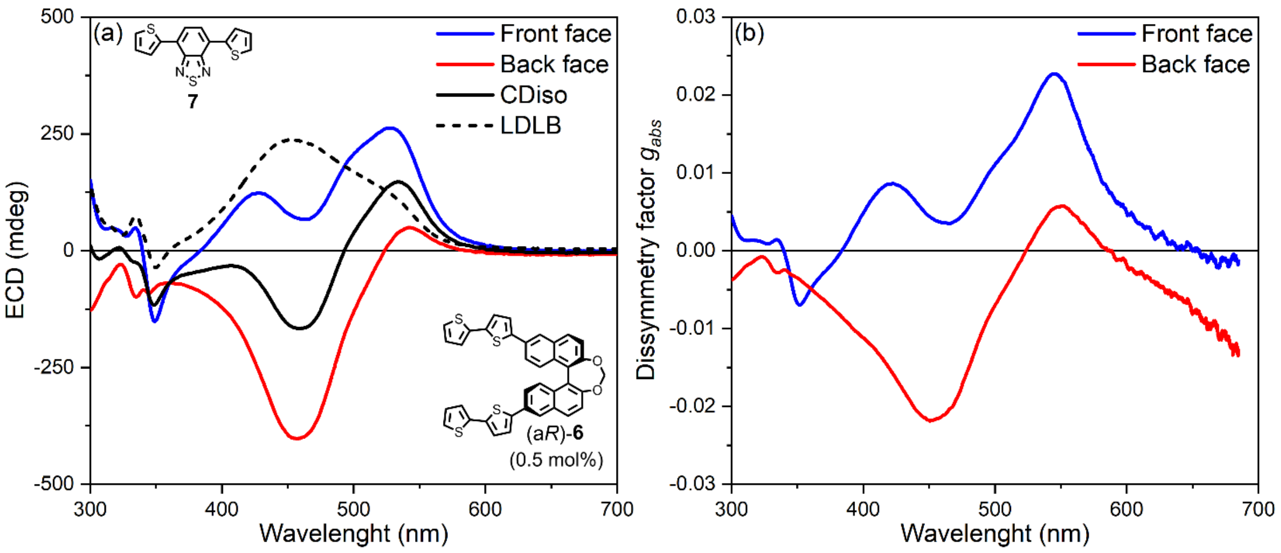

Figure S25.

(a) ECD and (b) dissymmetry factor g_{abs} spectra recorded for the front face (blue line) and the back face (red line) of as-cast thin film samples of blends of achiral π-conjugated dye **7** with methylene-bridged BINOL chiral inducer (a*R*)-**6**, prepared by spin coating technique from a 0.1 M $CHCl_3$ solution of **7** with 0.5 mol% of (a*R*)-**6**. For panel (a): black continuous line is the front-back semi-sum of ECD, *i.e.*, calculated CDiso contribution; black dashed line is the front-back semi-difference of ECD, *i.e.*, calculated LDLB contribution.

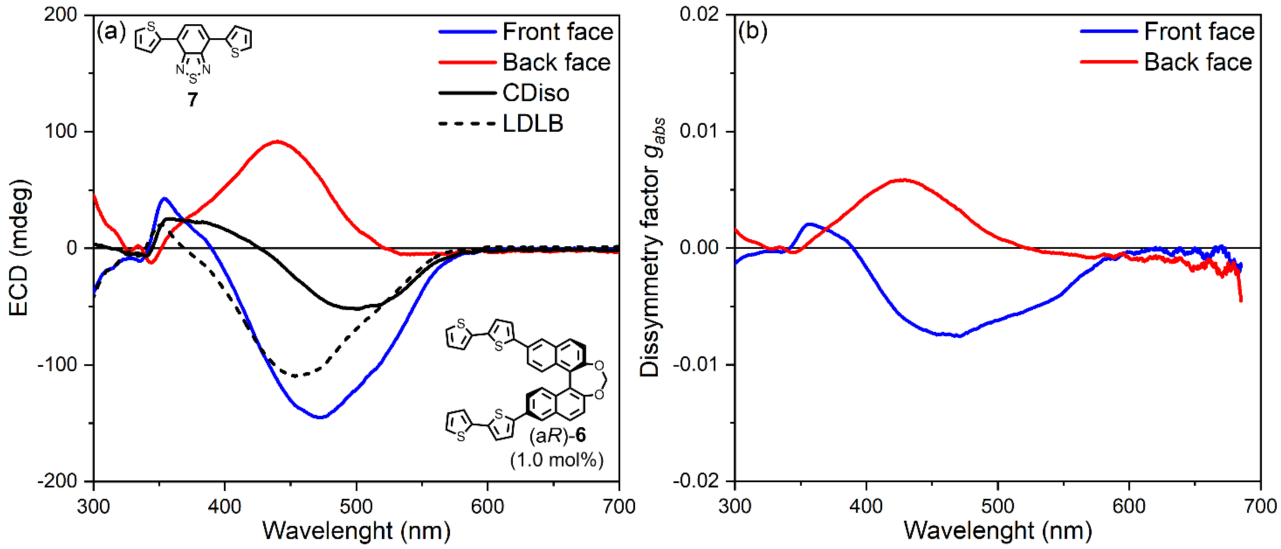

Figure S26.

(a) ECD and (b) dissymmetry factor g_{abs} spectra recorded for the front face (blue line) and the back face (red line) of as-cast thin film samples of blends of achiral π-conjugated dye **7** with methylene-bridged BINOL chiral inducer (a*R*)-**6**, prepared by spin coating technique from a 0.1 M $CHCl_3$ solution of **7** with 1.0 mol% of (a*R*)-**6**. For panel (a): black continuous line is the front-back semi-sum of ECD, *i.e.*, calculated CDiso contribution; black dashed line is the front-back semi-difference of ECD, *i.e.*, calculated LDLB contribution.

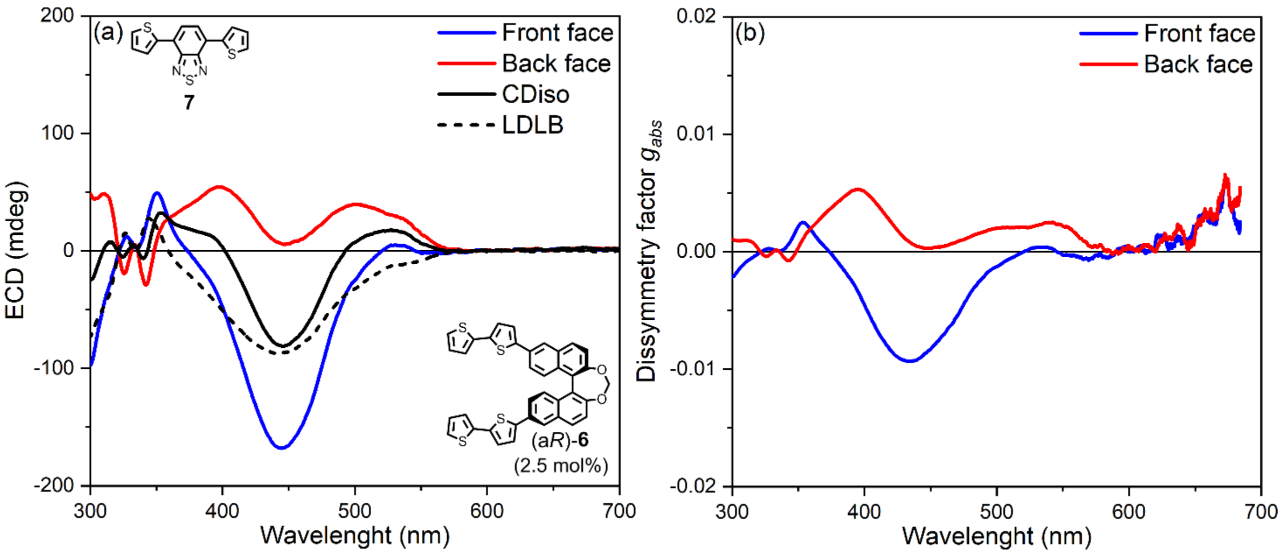

Figure S27.

(a) ECD and (b) dissymmetry factor g_{abs} spectra recorded for the front face (blue line) and the back face (red line) of as-cast thin film samples of blends of achiral π-conjugated dye **7** with methylene-bridged BINOL chiral inducer (a*R*)-**6**, prepared by spin coating technique from a 0.1 M $CHCl_3$ solution of **7** with 2.5 mol% of (a*R*)-**6**. For panel (a): black continuous line is the front-back semi-sum of ECD, *i.e.*, calculated CDiso contribution; black dashed line is the front-back semi-difference of ECD, *i.e.*, calculated LDLB contribution.

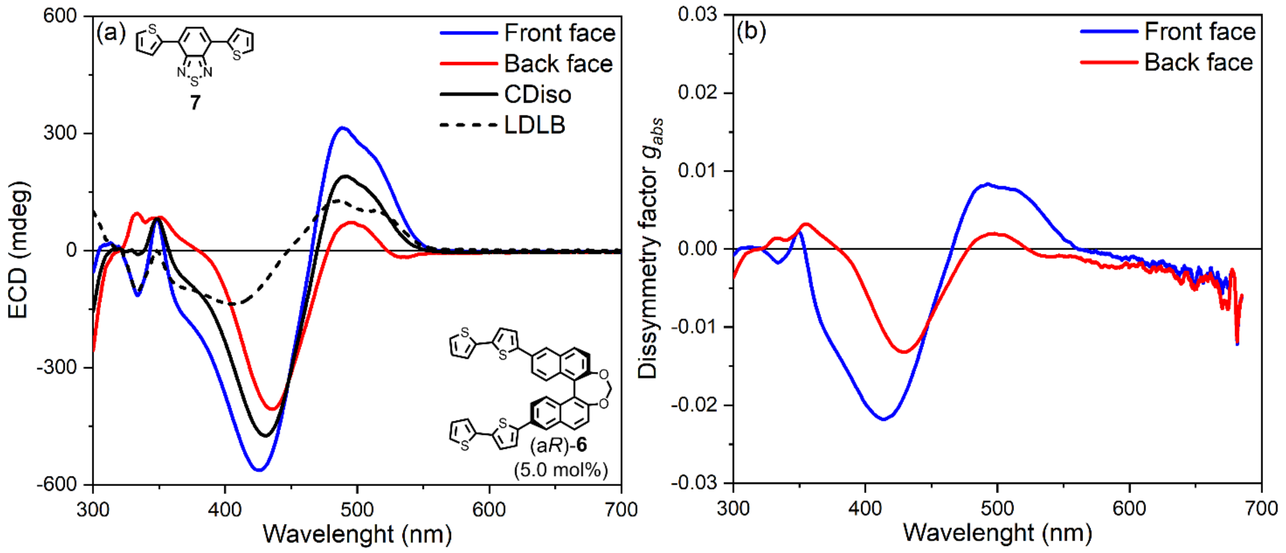

Figure S28.

(a) ECD and (b) dissymmetry factor g_{abs} spectra recorded for the front face (blue line) and the back face (red line) of as-cast thin film samples of blends of achiral π-conjugated dye **7** with methylene-bridged BINOL chiral inducer (a*R*)-**6**, prepared by spin coating technique from a 0.1 M $CHCl_3$ solution of **7** with 5.0 mol% of (a*R*)-**6**. For panel (a): black continuous line is the front-back semi-sum of ECD, *i.e.*, calculated CDiso contribution; black dashed line is the front-back semi-difference of ECD, *i.e.*, calculated LDLB contribution.

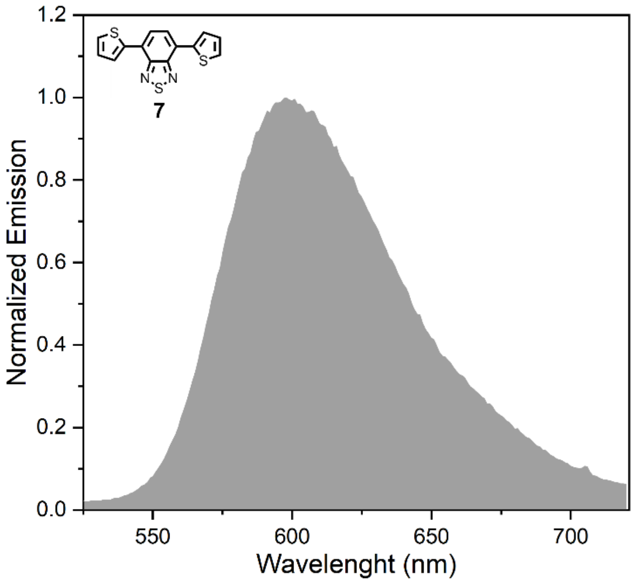

Figure S29.
Photoluminescence spectrum of as-cast samples of pure achiral π-conjugated dye **7**, prepared by spin coating technique from a 0.1 M $CHCl_3$ solution. Excitation wavelength: 365 nm.

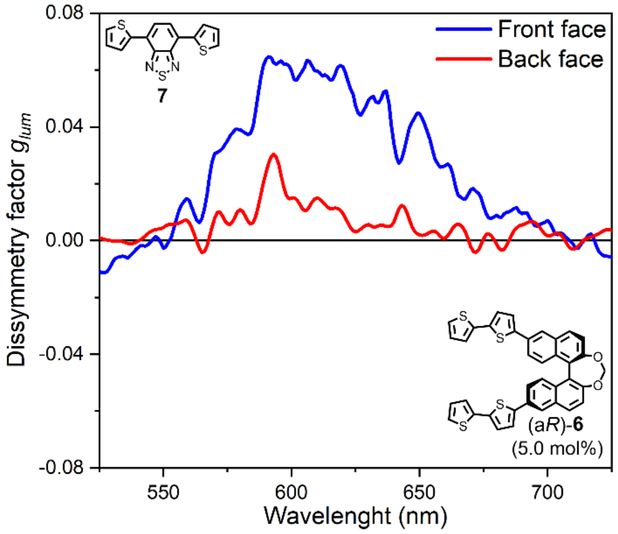

Figure S30.

Luminescence dissymmetry factor g_{lum} spectra for the front face (blue line) and the back face (red line) of as-cast thin film samples of blends of achiral π-conjugated dye **7** with methylene-bridged BINOL chiral inducer (a*R*)-**6**, prepared by spin coating technique from a 0.1 M $CHCl_3$ solution of **7** with 5.0 mol% of (a*R*)-**6**.

Supplementary Tables

Table S1.

Data for the first 24 calculated transitions of compound (a*R*)-**3** (8 conformers), at CAM-B3LYP/def2-TZVP/PCM//B3LYP-D3BJ/6-311+G(d,p) level. Rotational strengths are expressed in 10^{-40} cgs units. The structures and relative energies are reported in Figures S15.

Conformer 1					
Excited State 1:	Singlet-B	3.4406 eV	360.36 nm	f=1.4282	R(length)=530.2045
Excited State 2:	Singlet-A	3.4892 eV	355.34 nm	f=0.8279	R(length)=-578.1044
Excited State 3:	Singlet-B	3.9831 eV	311.28 nm	f=0.2047	R(length)=28.8874
Excited State 4:	Singlet-A	4.0284 eV	307.77 nm	f=0.0060	R(length)=-32.7544
Excited State 5:	Singlet-B	4.4182 eV	280.62 nm	f=0.2333	R(length)=52.0873
Excited State 6:	Singlet-A	4.4214 eV	280.42 nm	f=0.0004	R(length)=-13.2943
Excited State 7:	Singlet-B	4.6024 eV	269.39 nm	f=0.0013	R(length)=-1.3154
Excited State 8:	Singlet-A	4.6027 eV	269.37 nm	f=0.0001	R(length)=-0.2428
Excited State 9:	Singlet-B	4.8177 eV	257.35 nm	f=0.3349	R(length)=-67.5356
Excited State 10:	Singlet-A	4.8992 eV	253.07 nm	f=0.0503	R(length)=-31.0684
Excited State 11:	Singlet-B	5.0116 eV	247.39 nm	f=0.0012	R(length)=1.4199
Excited State 12:	Singlet-A	5.0124 eV	247.35 nm	f=0.0000	R(length)=0.8031
Excited State 13:	Singlet-B	5.0877 eV	243.69 nm	f=0.0226	R(length)=51.7832
Excited State 14:	Singlet-A	5.0902 eV	243.58 nm	f=0.0015	R(length)=-4.3874
Excited State 15:	Singlet-B	5.0932 eV	243.43 nm	f=0.2175	R(length)=-149.0217
Excited State 16:	Singlet-A	5.1371 eV	241.35 nm	f=0.0333	R(length)=90.7198
Excited State 17:	Singlet-B	5.2116 eV	237.90 nm	f=0.0298	R(length)=38.5818
Excited State 18:	Singlet-A	5.2265 eV	237.22 nm	f=0.0366	R(length)=-164.7688
Excited State 19:	Singlet-B	5.2774 eV	234.93 nm	f=0.2046	R(length)=-777.1127
Excited State 20:	Singlet-A	5.2926 eV	234.26 nm	f=0.1445	R(length)=777.5151
Excited State 21:	Singlet-B	5.4240 eV	228.59 nm	f=0.0872	R(length)=54.5458
Excited State 22:	Singlet-A	5.4267 eV	228.47 nm	f=0.0861	R(length)=28.5080
Excited State 23:	Singlet-B	5.4873 eV	225.95 nm	f=0.0001	R(length)=-2.0288
Excited State 24:	Singlet-A	5.4905 eV	225.82 nm	f=0.0116	R(length)=19.5131
Conformer 2					
Excited State 1:	Singlet-B	3.4563 eV	358.72 nm	f=1.6544	R(length)=123.6780
Excited State 2:	Singlet-A	3.5029 eV	353.95 nm	f=0.6219	R(length)=-7.9488
Excited State 3:	Singlet-B	3.9892 eV	310.80 nm	f=0.2136	R(length)=-11.6610
Excited State 4:	Singlet-A	4.0308 eV	307.59 nm	f=0.0014	R(length)=-9.8496
Excited State 5:	Singlet-B	4.4200 eV	280.50 nm	f=0.2273	R(length)=-34.1307
Excited State 6:	Singlet-A	4.4301 eV	279.87 nm	f=0.0029	R(length)=14.9479
Excited State 7:	Singlet-A	4.6088 eV	269.02 nm	f=0.0001	R(length)=0.3399
Excited State 8:	Singlet-B	4.6109 eV	268.90 nm	f=0.0010	R(length)=-2.7775
Excited State 9:	Singlet-B	4.8234 eV	257.05 nm	f=0.3118	R(length)=-117.0306
Excited State 10:	Singlet-A	4.9046 eV	252.79 nm	f=0.0502	R(length)=-35.2356
Excited State 11:	Singlet-B	5.0128 eV	247.34 nm	f=0.0017	R(length)=-0.6168
Excited State 12:	Singlet-A	5.0235 eV	246.81 nm	f=0.0026	R(length)=0.8655
Excited State 13:	Singlet-A	5.0863 eV	243.76 nm	f=0.0020	R(length)=-12.4620
Excited State 14:	Singlet-B	5.0868 eV	243.74 nm	f=0.0208	R(length)=-33.4376
Excited State 15:	Singlet-B	5.0964 eV	243.28 nm	f=0.1158	R(length)=-130.7568
Excited State 16:	Singlet-A	5.1469 eV	240.89 nm	f=0.1085	R(length)=-110.8497
Excited State 17:	Singlet-B	5.2228 eV	237.39 nm	f=0.0499	R(length)=62.3444
Excited State 18:	Singlet-A	5.2311 eV	237.01 nm	f=0.0241	R(length)=-117.8826
Excited State 19:	Singlet-B	5.2806 eV	234.79 nm	f=0.1504	R(length)=-757.1240
Excited State 20:	Singlet-A	5.2974 eV	234.05 nm	f=0.2219	R(length)=842.7899

Excited State 21:	Singlet-B	5.4283 eV	228.40 nm	f=0.0575	R(length)=-17.5641
Excited State 22:	Singlet-A	5.4346 eV	228.14 nm	f=0.1284	R(length)=-47.9502
Excited State 23:	Singlet-B	5.4958 eV	225.60 nm	f=0.0005	R(length)=3.8456
Excited State 24:	Singlet-A	5.4976 eV	225.53 nm	f=0.0029	R(length)=15.0881
Conformer 3					
Excited State 1:	Singlet-B	3.3951 eV	365.19 nm	f=1.4313	R(length)=634.0335
Excited State 2:	Singlet-A	3.4437 eV	360.04 nm	f=0.7937	R(length)=-488.1120
Excited State 3:	Singlet-B	3.9855 eV	311.09 nm	f=0.2052	R(length)=20.6329
Excited State 4:	Singlet-A	4.0308 eV	307.60 nm	f=0.0046	R(length)=-24.0516
Excited State 5:	Singlet-B	4.3963 eV	282.02 nm	f=0.2179	R(length)=-12.7353
Excited State 6:	Singlet-A	4.3980 eV	281.91 nm	f=0.0003	R(length)=9.9635
Excited State 7:	Singlet-B	4.5888 eV	270.19 nm	f=0.0006	R(length)=-0.7001
Excited State 8:	Singlet-A	4.5892 eV	270.17 nm	f=0.0000	R(length)=-0.0291
Excited State 9:	Singlet-B	4.8119 eV	257.66 nm	f=0.3223	R(length)=-71.6907
Excited State 10:	Singlet-A	4.8848 eV	253.81 nm	f=0.0402	R(length)=-44.2888
Excited State 11:	Singlet-A	4.9982 eV	248.06 nm	f=0.0019	R(length)=5.7125
Excited State 12:	Singlet-B	4.9988 eV	248.03 nm	f=0.0019	R(length)=-0.8573
Excited State 13:	Singlet-B	5.0557 eV	245.24 nm	f=0.1710	R(length)=-217.8862
Excited State 14:	Singlet-B	5.0897 eV	243.60 nm	f=0.0205	R(length)=-19.0732
Excited State 15:	Singlet-A	5.0947 eV	243.36 nm	f=0.0157	R(length)=-9.1124
Excited State 16:	Singlet-A	5.0995 eV	243.13 nm	f=0.0537	R(length)=35.5056
Excited State 17:	Singlet-B	5.1791 eV	239.39 nm	f=0.0450	R(length)=2.3898
Excited State 18:	Singlet-A	5.2121 eV	237.88 nm	f=0.0914	R(length)=-353.2294
Excited State 19:	Singlet-B	5.2565 eV	235.87 nm	f=0.2112	R(length)=-327.6914
Excited State 20:	Singlet-A	5.2671 eV	235.39 nm	f=0.0568	R(length)=542.2791
Excited State 21:	Singlet-B	5.3625 eV	231.20 nm	f=0.1199	R(length)=-218.5984
Excited State 22:	Singlet-A	5.3627 eV	231.20 nm	f=0.1239	R(length)=282.5044
Excited State 23:	Singlet-B	5.4586 eV	227.14 nm	f=0.0030	R(length)=-7.7801
Excited State 24:	Singlet-A	5.4631 eV	226.95 nm	f=0.0122	R(length)=16.5654
Conformer 4					
Excited State 1:	Singlet-B	3.3684 eV	368.08 nm	f=1.6192	R(length)=174.5763
Excited State 2:	Singlet-A	3.4176 eV	362.78 nm	f=0.6401	R(length)=-231.8805
Excited State 3:	Singlet-B	3.9803 eV	311.50 nm	f=0.1990	R(length)=0.2313
Excited State 4:	Singlet-A	4.0218 eV	308.28 nm	f=0.0015	R(length)=-12.2219
Excited State 5:	Singlet-B	4.3868 eV	282.63 nm	f=0.2159	R(length)=-3.6968
Excited State 6:	Singlet-A	4.3916 eV	282.32 nm	f=0.0036	R(length)=31.7136
Excited State 7:	Singlet-A	4.5724 eV	271.16 nm	f=0.0001	R(length)=0.0961
Excited State 8:	Singlet-B	4.5735 eV	271.09 nm	f=0.0010	R(length)=-1.4313
Excited State 9:	Singlet-B	4.8041 eV	258.08 nm	f=0.3088	R(length)=-110.6522
Excited State 10:	Singlet-A	4.8731 eV	254.43 nm	f=0.0376	R(length)=-15.2697
Excited State 11:	Singlet-B	4.9708 eV	249.42 nm	f=0.0011	R(length)=0.2911
Excited State 12:	Singlet-A	4.9776 eV	249.08 nm	f=0.0028	R(length)=6.7397
Excited State 13:	Singlet-B	5.0419 eV	245.91 nm	f=0.1569	R(length)=-196.5756
Excited State 14:	Singlet-A	5.0814 eV	244.00 nm	f=0.0298	R(length)=45.8110
Excited State 15:	Singlet-B	5.0839 eV	243.87 nm	f=0.0305	R(length)=0.8426
Excited State 16:	Singlet-A	5.0956 eV	243.32 nm	f=0.0274	R(length)=45.7290
Excited State 17:	Singlet-B	5.1800 eV	239.35 nm	f=0.0694	R(length)=50.5734
Excited State 18:	Singlet-A	5.2091 eV	238.02 nm	f=0.0207	R(length)=-86.8605
Excited State 19:	Singlet-B	5.2491 eV	236.20 nm	f=0.0891	R(length)=-624.3622
Excited State 20:	Singlet-A	5.2669 eV	235.40 nm	f=0.2275	R(length)=623.8929
Excited State 21:	Singlet-B	5.3501 eV	231.74 nm	f=0.0801	R(length)=-217.0339
Excited State 22:	Singlet-A	5.3544 eV	231.55 nm	f=0.1559	R(length)=275.2775
Excited State 23:	Singlet-B	5.4468 eV	227.63 nm	f=0.0000	R(length)=-0.2146

Excited State 24:	Singlet-A	5.4508 eV	227.46 nm	f=0.0030	R(length)=3.9524
Conformer 5					
Excited State 1:	Singlet-A	3.4128 eV	363.30 nm	f=1.2751	R(length)=-445.1220
Excited State 2:	Singlet-A	3.4817 eV	356.10 nm	f=1.0303	R(length)=254.5586
Excited State 3:	Singlet-A	3.9546 eV	313.52 nm	f=0.0959	R(length)=15.3149
Excited State 4:	Singlet-A	4.0151 eV	308.79 nm	f=0.0636	R(length)=-13.9526
Excited State 5:	Singlet-A	4.3982 eV	281.90 nm	f=0.1198	R(length)=8.0772
Excited State 6:	Singlet-A	4.4247 eV	280.21 nm	f=0.0243	R(length)=4.7575
Excited State 7:	Singlet-A	4.5847 eV	270.43 nm	f=0.0003	R(length)=0.0660
Excited State 8:	Singlet-A	4.6087 eV	269.02 nm	f=0.0005	R(length)=-0.1172
Excited State 9:	Singlet-A	4.7957 eV	258.53 nm	f=0.3254	R(length)=-110.6515
Excited State 10:	Singlet-A	4.8791 eV	254.11 nm	f=0.0482	R(length)=22.6225
Excited State 11:	Singlet-A	4.9688 eV	249.52 nm	f=0.0014	R(length)=-2.6602
Excited State 12:	Singlet-A	5.0001 eV	247.96 nm	f=0.0005	R(length)=0.2497
Excited State 13:	Singlet-A	5.0662 eV	244.73 nm	f=0.1080	R(length)=-175.7269
Excited State 14:	Singlet-A	5.0717 eV	244.46 nm	f=0.0957	R(length)=44.9266
Excited State 15:	Singlet-A	5.0949 eV	243.35 nm	f=0.0181	R(length)=13.9123
Excited State 16:	Singlet-A	5.1336 eV	241.51 nm	f=0.1094	R(length)=59.3173
Excited State 17:	Singlet-A	5.2072 eV	238.10 nm	f=0.0761	R(length)=160.8511
Excited State 18:	Singlet-A	5.2443 eV	236.42 nm	f=0.0306	R(length)=-63.9755
Excited State 19:	Singlet-A	5.2627 eV	235.59 nm	f=0.1545	R(length)=239.4485
Excited State 20:	Singlet-A	5.2856 eV	234.57 nm	f=0.1341	R(length)=-109.3164
Excited State 21:	Singlet-A	5.3634 eV	231.17 nm	f=0.1206	R(length)=-1.4274
Excited State 22:	Singlet-A	5.4241 eV	228.58 nm	f=0.0958	R(length)=-20.0239
Excited State 23:	Singlet-A	5.4889 eV	225.88 nm	f=0.0028	R(length)=-1.9288
Excited State 24:	Singlet-A	5.4942 eV	225.67 nm	f=0.0013	R(length)=-1.1207
Conformer 6					
Excited State 1:	Singlet-A	3.4695 eV	357.36 nm	f=1.4882	R(length)=-2104.6786
Excited State 2:	Singlet-A	3.5167 eV	352.56 nm	f=0.9244	R(length)=1951.1232
Excited State 3:	Singlet-A	3.9545 eV	313.53 nm	f=0.1186	R(length)=-62.4980
Excited State 4:	Singlet-A	3.9959 eV	310.28 nm	f=0.0030	R(length)=13.3547
Excited State 5:	Singlet-A	4.4315 eV	279.78 nm	f=0.0103	R(length)=-6.1764
Excited State 6:	Singlet-A	4.4317 eV	279.76 nm	f=0.0602	R(length)=-79.8181
Excited State 7:	Singlet-A	4.6060 eV	269.18 nm	f=0.0009	R(length)=0.4060
Excited State 8:	Singlet-A	4.6064 eV	269.16 nm	f=0.0000	R(length)=0.0074
Excited State 9:	Singlet-A	4.7993 eV	258.34 nm	f=0.3670	R(length)=-126.7517
Excited State 10:	Singlet-A	4.8855 eV	253.78 nm	f=0.0430	R(length)=109.0370
Excited State 11:	Singlet-A	4.9969 eV	248.12 nm	f=0.0026	R(length)=-0.0071
Excited State 12:	Singlet-A	5.0008 eV	247.93 nm	f=0.0003	R(length)=3.3655
Excited State 13:	Singlet-A	5.1038 eV	242.93 nm	f=0.1479	R(length)=-206.9934
Excited State 14:	Singlet-A	5.1046 eV	242.89 nm	f=0.0118	R(length)=73.6216
Excited State 15:	Singlet-A	5.1225 eV	242.04 nm	f=0.0708	R(length)=83.2201
Excited State 16:	Singlet-A	5.1588 eV	240.34 nm	f=0.0654	R(length)=148.9705
Excited State 17:	Singlet-A	5.1807 eV	239.32 nm	f=0.0331	R(length)=13.4938
Excited State 18:	Singlet-A	5.2173 eV	237.64 nm	f=0.1608	R(length)=446.9262
Excited State 19:	Singlet-A	5.2931 eV	234.24 nm	f=0.2412	R(length)=108.3326
Excited State 20:	Singlet-A	5.2986 eV	233.99 nm	f=0.0313	R(length)=-314.2166
Excited State 21:	Singlet-A	5.4614 eV	227.02 nm	f=0.1193	R(length)=163.2686
Excited State 22:	Singlet-A	5.4621 eV	226.99 nm	f=0.2183	R(length)=-197.3425
Excited State 23:	Singlet-A	5.5185 eV	224.67 nm	f=0.0121	R(length)=-9.6225
Excited State 24:	Singlet-A	5.5222 eV	224.52 nm	f=0.0170	R(length)=38.6069
Conformer 7					
Excited State 1:	Singlet-B	3.4663 eV	357.69 nm	f=1.0535	R(length)=-1753.5272

Excited State 2:	Singlet-A	3.5189 eV	352.33 nm	f=1.3171	R(length)=1619.4430
Excited State 3:	Singlet-B	3.9559 eV	313.42 nm	f=0.1167	R(length)=48.8970
Excited State 4:	Singlet-A	3.9912 eV	310.64 nm	f=0.0128	R(length)=8.4401
Excited State 5:	Singlet-B	4.4258 eV	280.14 nm	f=0.0539	R(length)=63.7568
Excited State 6:	Singlet-A	4.4350 eV	279.56 nm	f=0.0183	R(length)=12.8965
Excited State 7:	Singlet-A	4.5963 eV	269.75 nm	f=0.0006	R(length)=0.8433
Excited State 8:	Singlet-B	4.5996 eV	269.55 nm	f=0.0002	R(length)=-0.3453
Excited State 9:	Singlet-B	4.7986 eV	258.37 nm	f=0.3693	R(length)=-251.0340
Excited State 10:	Singlet-A	4.8853 eV	253.79 nm	f=0.0564	R(length)=72.6144
Excited State 11:	Singlet-B	4.9714 eV	249.39 nm	f=0.0001	R(length)=0.6937
Excited State 12:	Singlet-A	4.9846 eV	248.73 nm	f=0.0005	R(length)=3.1247
Excited State 13:	Singlet-B	5.1038 eV	242.92 nm	f=0.1749	R(length)=-356.8839
Excited State 14:	Singlet-A	5.1087 eV	242.69 nm	f=0.0217	R(length)=82.7706
Excited State 15:	Singlet-B	5.1279 eV	241.79 nm	f=0.1500	R(length)=-149.4150
Excited State 16:	Singlet-A	5.1600 eV	240.28 nm	f=0.0152	R(length)=97.1962
Excited State 17:	Singlet-B	5.1846 eV	239.14 nm	f=0.0197	R(length)=-1.8364
Excited State 18:	Singlet-A	5.2203 eV	237.51 nm	f=0.1419	R(length)=322.2365
Excited State 19:	Singlet-B	5.2927 eV	234.26 nm	f=0.2463	R(length)=2.5377
Excited State 20:	Singlet-A	5.2972 eV	234.06 nm	f=0.0001	R(length)=-11.0172
Excited State 21:	Singlet-B	5.4594 eV	227.10 nm	f=0.1829	R(length)=-269.1433
Excited State 22:	Singlet-A	5.4612 eV	227.03 nm	f=0.1785	R(length)=234.4349
Excited State 23:	Singlet-B	5.5096 eV	225.03 nm	f=0.0055	R(length)=-0.3417
Excited State 24:	Singlet-A	5.5141 eV	224.85 nm	f=0.0025	R(length)=-1.8202
Conformer 8					
Excited State 1:	Singlet-B	3.4736 eV	356.93 nm	f=1.4377	R(length)=-1997.9486
Excited State 2:	Singlet-A	3.5233 eV	351.90 nm	f=0.9625	R(length)=2045.6800
Excited State 3:	Singlet-B	3.9499 eV	313.89 nm	f=0.1010	R(length)=-32.8393
Excited State 4:	Singlet-A	3.9861 eV	311.04 nm	f=0.0010	R(length)=7.1831
Excited State 5:	Singlet-B	4.4317 eV	279.77 nm	f=0.0590	R(length)=-40.5905
Excited State 6:	Singlet-A	4.4324 eV	279.72 nm	f=0.0026	R(length)=18.0851
Excited State 7:	Singlet-A	4.6145 eV	268.68 nm	f=0.0000	R(length)=0.0614
Excited State 8:	Singlet-B	4.6146 eV	268.68 nm	f=0.0006	R(length)=1.4222
Excited State 9:	Singlet-B	4.7903 eV	258.82 nm	f=0.3681	R(length)=-246.7551
Excited State 10:	Singlet-A	4.8798 eV	254.08 nm	f=0.0340	R(length)=82.1675
Excited State 11:	Singlet-B	4.9876 eV	248.59 nm	f=0.0033	R(length)=-0.9020
Excited State 12:	Singlet-A	4.9935 eV	248.29 nm	f=0.0002	R(length)=2.3421
Excited State 13:	Singlet-A	5.0765 eV	244.23 nm	f=0.0071	R(length)=53.1932
Excited State 14:	Singlet-B	5.0783 eV	244.14 nm	f=0.0608	R(length)=-142.3270
Excited State 15:	Singlet-B	5.1049 eV	242.87 nm	f=0.2588	R(length)=-214.7121
Excited State 16:	Singlet-A	5.1766 eV	239.51 nm	f=0.1132	R(length)=385.2204
Excited State 17:	Singlet-A	5.2562 eV	235.88 nm	f=0.0455	R(length)=11.5813
Excited State 18:	Singlet-B	5.2594 eV	235.74 nm	f=0.0197	R(length)=-33.3101
Excited State 19:	Singlet-B	5.2869 eV	234.51 nm	f=0.2066	R(length)=427.1542
Excited State 20:	Singlet-A	5.2928 eV	234.25 nm	f=0.0687	R(length)=-495.1984
Excited State 21:	Singlet-A	5.4276 eV	228.43 nm	f=0.0528	R(length)=169.6285
Excited State 22:	Singlet-B	5.4315 eV	228.27 nm	f=0.1148	R(length)=-132.0193
Excited State 23:	Singlet-A	5.5276 eV	224.30 nm	f=0.0762	R(length)=227.4076
Excited State 24:	Singlet-B	5.5296 eV	224.22 nm	f=0.0027	R(length)=3.6331

Table S2.

Data for the first 24 calculated transitions of compound (a*R*)-**6** (8 conformers), at CAM-B3LYP/def2-TZVP/PCM//B3LYP-D3BJ/6-311+G(d,p) level. Rotational strengths are expressed in 10^{-40} cgs units. The structures and relative energies are reported in Figures S16.

Conformer 1					
Excited State 1:	Singlet-B	3.4381 eV	360.62 nm	f=1.3273	R(length)=-30.0788
Excited State 2:	Singlet-A	3.4907 eV	355.19 nm	f=0.8933	R(length)=-1.8669
Excited State 3:	Singlet-B	4.0004 eV	309.93 nm	f=0.4517	R(length)=-34.1783
Excited State 4:	Singlet-A	4.0357 eV	307.22 nm	f=0.0042	R(length)=0.3385
Excited State 5:	Singlet-B	4.1718 eV	297.20 nm	f=0.1519	R(length)=-30.7023
Excited State 6:	Singlet-A	4.3302 eV	286.33 nm	f=0.0005	R(length)=4.5542
Excited State 7:	Singlet-B	4.5571 eV	272.07 nm	f=0.0136	R(length)=114.4102
Excited State 8:	Singlet-A	4.5871 eV	270.29 nm	f=0.0088	R(length)=-2.0930
Excited State 9:	Singlet-B	4.7960 eV	258.52 nm	f=0.1344	R(length)=74.1088
Excited State 10:	Singlet-A	4.8592 eV	255.15 nm	f=0.0747	R(length)=38.5806
Excited State 11:	Singlet-B	4.9387 eV	251.05 nm	f=0.0357	R(length)=37.8738
Excited State 12:	Singlet-A	4.9970 eV	248.12 nm	f=0.0335	R(length)=-5.4128
Excited State 13:	Singlet-B	5.0729 eV	244.41 nm	f=0.0017	R(length)=30.3241
Excited State 14:	Singlet-A	5.0893 eV	243.62 nm	f=0.0149	R(length)=42.4545
Excited State 15:	Singlet-B	5.1147 eV	242.41 nm	f=0.0747	R(length)=22.7998
Excited State 16:	Singlet-A	5.1433 eV	241.06 nm	f=0.0117	R(length)=35.4697
Excited State 17:	Singlet-A	5.1858 eV	239.09 nm	f=0.2362	R(length)=210.4990
Excited State 18:	Singlet-B	5.1891 eV	238.93 nm	f=0.0160	R(length)=37.2696
Excited State 19:	Singlet-B	5.2600 eV	235.71 nm	f=0.1100	R(length)=555.9697
Excited State 20:	Singlet-A	5.2639 eV	235.54 nm	f=0.2438	R(length)=-570.8282
Excited State 21:	Singlet-A	5.3024 eV	233.83 nm	f=0.0895	R(length)=-228.5339
Excited State 22:	Singlet-B	5.3655 eV	231.08 nm	f=0.0606	R(length)=62.8917
Excited State 23:	Singlet-B	5.4449 eV	227.71 nm	f=0.0192	R(length)=-16.4676
Excited State 24:	Singlet-A	5.4674 eV	226.77 nm	f=0.0153	R(length)=50.5988
Conformer 2					
Excited State 1:	Singlet-B	3.4521 eV	359.16 nm	f=1.1034	R(length)=-646.1926
Excited State 2:	Singlet-A	3.5055 eV	353.68 nm	f=1.1025	R(length)=755.9058
Excited State 3:	Singlet-B	4.0010 eV	309.88 nm	f=0.4322	R(length)=-130.7173
Excited State 4:	Singlet-A	4.0395 eV	306.93 nm	f=0.0075	R(length)=13.0648
Excited State 5:	Singlet-B	4.1741 eV	297.04 nm	f=0.1491	R(length)=-48.5962
Excited State 6:	Singlet-A	4.3339 eV	286.08 nm	f=0.0052	R(length)=44.6222
Excited State 7:	Singlet-B	4.5658 eV	271.55 nm	f=0.0370	R(length)=155.8184
Excited State 8:	Singlet-A	4.5935 eV	269.91 nm	f=0.0089	R(length)=-8.2974
Excited State 9:	Singlet-B	4.7956 eV	258.54 nm	f=0.1429	R(length)=60.0544
Excited State 10:	Singlet-A	4.8645 eV	254.88 nm	f=0.0791	R(length)=20.0563
Excited State 11:	Singlet-B	4.9474 eV	250.60 nm	f=0.0380	R(length)=29.4109
Excited State 12:	Singlet-A	4.9979 eV	248.07 nm	f=0.0267	R(length)=-29.2312
Excited State 13:	Singlet-B	5.0809 eV	244.02 nm	f=0.0384	R(length)=-26.2131
Excited State 14:	Singlet-A	5.0976 eV	243.22 nm	f=0.0009	R(length)=-10.9136
Excited State 15:	Singlet-B	5.1188 eV	242.22 nm	f=0.1287	R(length)=103.2573
Excited State 16:	Singlet-A	5.1421 eV	241.11 nm	f=0.0029	R(length)=-12.8760
Excited State 17:	Singlet-A	5.1896 eV	238.91 nm	f=0.2279	R(length)=68.2584
Excited State 18:	Singlet-B	5.1952 eV	238.65 nm	f=0.0065	R(length)=27.0415
Excited State 19:	Singlet-A	5.2641 eV	235.53 nm	f=0.1619	R(length)=-590.2251
Excited State 20:	Singlet-B	5.2659 eV	235.45 nm	f=0.1667	R(length)=716.6425
Excited State 21:	Singlet-A	5.3041 eV	233.75 nm	f=0.0700	R(length)=-270.9423
Excited State 22:	Singlet-B	5.3572 eV	231.43 nm	f=0.0600	R(length)=71.4172

Excited State 23:	Singlet-B	5.4485 eV	227.56 nm	f=0.0312	R(length)=-14.2136
Excited State 24:	Singlet-A	5.4734 eV	226.52 nm	f=0.0286	R(length)=26.2975
Conformer 3					
Excited State 1:	Singlet-B	3.4020 eV	364.44 nm	f=1.2878	R(length)=-71.8473
Excited State 2:	Singlet-A	3.4548 eV	358.87 nm	f=0.9016	R(length)=228.7619
Excited State 3:	Singlet-B	4.0016 eV	309.84 nm	f=0.4520	R(length)=-59.8379
Excited State 4:	Singlet-A	4.0349 eV	307.28 nm	f=0.0049	R(length)=4.0789
Excited State 5:	Singlet-B	4.1615 eV	297.93 nm	f=0.1343	R(length)=-37.1342
Excited State 6:	Singlet-A	4.3157 eV	287.29 nm	f=0.0002	R(length)=6.7103
Excited State 7:	Singlet-B	4.5490 eV	272.55 nm	f=0.0175	R(length)=116.5230
Excited State 8:	Singlet-A	4.5748 eV	271.01 nm	f=0.0113	R(length)=-7.7143
Excited State 9:	Singlet-B	4.7783 eV	259.48 nm	f=0.1424	R(length)=91.9813
Excited State 10:	Singlet-A	4.8477 eV	255.76 nm	f=0.0589	R(length)=-3.8670
Excited State 11:	Singlet-B	4.9146 eV	252.28 nm	f=0.0300	R(length)=39.2961
Excited State 12:	Singlet-A	4.9831 eV	248.81 nm	f=0.0288	R(length)=-14.8019
Excited State 13:	Singlet-B	5.0508 eV	245.47 nm	f=0.0429	R(length)=140.9710
Excited State 14:	Singlet-A	5.0765 eV	244.23 nm	f=0.0178	R(length)=-54.4530
Excited State 15:	Singlet-B	5.1022 eV	243.00 nm	f=0.0725	R(length)=61.0095
Excited State 16:	Singlet-A	5.1317 eV	241.60 nm	f=0.0157	R(length)=-11.1252
Excited State 17:	Singlet-A	5.1752 eV	239.57 nm	f=0.2228	R(length)=-136.5747
Excited State 18:	Singlet-B	5.1851 eV	239.12 nm	f=0.0156	R(length)=1.6413
Excited State 19:	Singlet-B	5.2474 eV	236.28 nm	f=0.0633	R(length)=369.6898
Excited State 20:	Singlet-A	5.2485 eV	236.23 nm	f=0.1679	R(length)=-240.2897
Excited State 21:	Singlet-A	5.2718 eV	235.18 nm	f=0.1859	R(length)=-220.1864
Excited State 22:	Singlet-B	5.3466 eV	231.89 nm	f=0.0571	R(length)=-10.8311
Excited State 23:	Singlet-B	5.3825 eV	230.35 nm	f=0.0292	R(length)=83.8600
Excited State 24:	Singlet-A	5.4121 eV	229.09 nm	f=0.0200	R(length)=-15.0345
Conformer 4					
Excited State 1:	Singlet-B	3.4113 eV	363.45 nm	f=1.1320	R(length)=-673.3389
Excited State 2:	Singlet-A	3.4644 eV	357.88 nm	f=1.0420	R(length)=585.5407
Excited State 3:	Singlet-B	4.0051 eV	309.56 nm	f=0.4433	R(length)=-108.0677
Excited State 4:	Singlet-A	4.0410 eV	306.82 nm	f=0.0074	R(length)=6.0629
Excited State 5:	Singlet-B	4.1630 eV	297.83 nm	f=0.1347	R(length)=-38.0996
Excited State 6:	Singlet-A	4.3183 eV	287.12 nm	f=0.0015	R(length)=17.1972
Excited State 7:	Singlet-B	4.5553 eV	272.18 nm	f=0.0289	R(length)=175.8752
Excited State 8:	Singlet-A	4.5811 eV	270.64 nm	f=0.0117	R(length)=-4.4251
Excited State 9:	Singlet-B	4.7801 eV	259.38 nm	f=0.1433	R(length)=61.4055
Excited State 10:	Singlet-A	4.8497 eV	255.65 nm	f=0.0616	R(length)=55.0523
Excited State 11:	Singlet-B	4.9226 eV	251.87 nm	f=0.0292	R(length)=39.4542
Excited State 12:	Singlet-A	4.9886 eV	248.54 nm	f=0.0272	R(length)=-12.3464
Excited State 13:	Singlet-B	5.0576 eV	245.15 nm	f=0.0481	R(length)=170.1082
Excited State 14:	Singlet-A	5.0791 eV	244.10 nm	f=0.0219	R(length)=10.7237
Excited State 15:	Singlet-B	5.1012 eV	243.05 nm	f=0.0613	R(length)=-19.3678
Excited State 16:	Singlet-A	5.1298 eV	241.69 nm	f=0.0129	R(length)=14.9978
Excited State 17:	Singlet-A	5.1776 eV	239.46 nm	f=0.3125	R(length)=298.6933
Excited State 18:	Singlet-B	5.1877 eV	239.00 nm	f=0.0110	R(length)=-12.1702
Excited State 19:	Singlet-A	5.2466 eV	236.31 nm	f=0.0921	R(length)=-493.5292
Excited State 20:	Singlet-B	5.2537 eV	235.99 nm	f=0.2167	R(length)=637.0539
Excited State 21:	Singlet-A	5.2707 eV	235.23 nm	f=0.0614	R(length)=-369.4091
Excited State 22:	Singlet-B	5.3353 eV	232.38 nm	f=0.0489	R(length)=-32.7300
Excited State 23:	Singlet-B	5.3888 eV	230.08 nm	f=0.0359	R(length)=100.2780
Excited State 24:	Singlet-A	5.4196 eV	228.77 nm	f=0.0387	R(length)=-45.9097
Conformer 5					

Excited State 1:	Singlet-B	3.4787 eV	356.41 nm	f=0.6207	R(length)=1266.7698
Excited State 2:	Singlet-A	3.5382 eV	350.41 nm	f=1.6665	R(length)=-1121.1602
Excited State 3:	Singlet-B	3.9618 eV	312.95 nm	f=0.2787	R(length)=-99.0755
Excited State 4:	Singlet-A	4.0143 eV	308.86 nm	f=0.0195	R(length)=-18.9982
Excited State 5:	Singlet-B	4.1882 eV	296.03 nm	f=0.1155	R(length)=-35.2832
Excited State 6:	Singlet-A	4.3232 eV	286.79 nm	f=0.0442	R(length)=-3.7989
Excited State 7:	Singlet-B	4.5436 eV	272.87 nm	f=0.0348	R(length)=85.7350
Excited State 8:	Singlet-A	4.5851 eV	270.41 nm	f=0.0015	R(length)=-0.7422
Excited State 9:	Singlet-B	4.8017 eV	258.21 nm	f=0.1551	R(length)=144.9455
Excited State 10:	Singlet-A	4.8628 eV	254.96 nm	f=0.0911	R(length)=-64.7237
Excited State 11:	Singlet-B	4.9366 eV	251.15 nm	f=0.0165	R(length)=-17.6392
Excited State 12:	Singlet-A	4.9832 eV	248.80 nm	f=0.0124	R(length)=8.2864
Excited State 13:	Singlet-B	5.1085 eV	242.70 nm	f=0.1552	R(length)=239.1888
Excited State 14:	Singlet-A	5.1284 eV	241.76 nm	f=0.0034	R(length)=-15.9668
Excited State 15:	Singlet-B	5.1286 eV	241.75 nm	f=0.0266	R(length)=124.7005
Excited State 16:	Singlet-A	5.1481 eV	240.84 nm	f=0.0190	R(length)=-70.7892
Excited State 17:	Singlet-A	5.2029 eV	238.30 nm	f=0.3367	R(length)=-343.6232
Excited State 18:	Singlet-B	5.2087 eV	238.03 nm	f=0.0114	R(length)=5.0915
Excited State 19:	Singlet-A	5.2720 eV	235.17 nm	f=0.0001	R(length)=-7.5873
Excited State 20:	Singlet-B	5.2838 eV	234.65 nm	f=0.1947	R(length)=114.2279
Excited State 21:	Singlet-A	5.3049 eV	233.72 nm	f=0.0059	R(length)=-55.4048
Excited State 22:	Singlet-B	5.3627 eV	231.20 nm	f=0.1121	R(length)=50.7667
Excited State 23:	Singlet-B	5.4897 eV	225.85 nm	f=0.0505	R(length)=77.2777
Excited State 24:	Singlet-A	5.5562 eV	223.15 nm	f=0.0712	R(length)=61.2360
Conformer 6					
Excited State 1:	Singlet-B	3.4863 eV	355.63 nm	f=0.9321	R(length)=1992.4673
Excited State 2:	Singlet-A	3.5407 eV	350.17 nm	f=1.3956	R(length)=-1786.3978
Excited State 3:	Singlet-B	3.9682 eV	312.44 nm	f=0.2956	R(length)=128.8487
Excited State 4:	Singlet-A	4.0197 eV	308.44 nm	f=0.0099	R(length)=-29.7772
Excited State 5:	Singlet-B	4.1863 eV	296.16 nm	f=0.1288	R(length)=20.3984
Excited State 6:	Singlet-A	4.3240 eV	286.73 nm	f=0.0163	R(length)=-36.5787
Excited State 7:	Singlet-B	4.5437 eV	272.87 nm	f=0.0210	R(length)=-64.1493
Excited State 8:	Singlet-A	4.5922 eV	269.99 nm	f=0.0026	R(length)=2.2103
Excited State 9:	Singlet-B	4.8059 eV	257.98 nm	f=0.1454	R(length)=70.0383
Excited State 10:	Singlet-A	4.8614 eV	255.04 nm	f=0.0944	R(length)=-125.6609
Excited State 11:	Singlet-B	4.9473 eV	250.61 nm	f=0.0158	R(length)=31.0414
Excited State 12:	Singlet-A	4.9927 eV	248.33 nm	f=0.0162	R(length)=29.0665
Excited State 13:	Singlet-B	5.1072 eV	242.76 nm	f=0.0655	R(length)=-132.4876
Excited State 14:	Singlet-B	5.1341 eV	241.49 nm	f=0.0479	R(length)=142.1285
Excited State 15:	Singlet-A	5.1398 eV	241.22 nm	f=0.0053	R(length)=-30.1273
Excited State 16:	Singlet-A	5.1466 eV	240.90 nm	f=0.0368	R(length)=-6.1163
Excited State 17:	Singlet-A	5.2036 eV	238.27 nm	f=0.3719	R(length)=-510.9965
Excited State 18:	Singlet-B	5.2087 eV	238.03 nm	f=0.0343	R(length)=3.5169
Excited State 19:	Singlet-A	5.2755 eV	235.02 nm	f=0.0084	R(length)=94.0942
Excited State 20:	Singlet-B	5.2863 eV	234.54 nm	f=0.2130	R(length)=173.5525
Excited State 21:	Singlet-A	5.3026 eV	233.82 nm	f=0.0003	R(length)=20.1766
Excited State 22:	Singlet-B	5.3717 eV	230.81 nm	f=0.0743	R(length)=-39.2489
Excited State 23:	Singlet-B	5.4815 eV	226.19 nm	f=0.0793	R(length)=62.0008
Excited State 24:	Singlet-A	5.5434 eV	223.66 nm	f=0.0484	R(length)=95.8263
Conformer 7					
Excited State 1:	Singlet-B	3.4714 eV	357.16 nm	f=0.6310	R(length)=-1416.7539
Excited State 2:	Singlet-A	3.5323 eV	351.00 nm	f=1.6437	R(length)=1085.3669
Excited State 3:	Singlet-B	3.9557 eV	313.43 nm	f=0.2451	R(length)=59.8922

Excited State 4:	Singlet-A	4.0064 eV	309.47 nm	f=0.0090	R(length)=10.7212
Excited State 5:	Singlet-B	4.1937 eV	295.64 nm	f=0.1292	R(length)=20.7198
Excited State 6:	Singlet-A	4.3160 eV	287.27 nm	f=0.0307	R(length)=5.3682
Excited State 7:	Singlet-B	4.5278 eV	273.83 nm	f=0.0459	R(length)=-27.1388
Excited State 8:	Singlet-A	4.5954 eV	269.80 nm	f=0.0010	R(length)=0.0472
Excited State 9:	Singlet-B	4.8013 eV	258.23 nm	f=0.1268	R(length)=-41.9094
Excited State 10:	Singlet-A	4.8517 eV	255.55 nm	f=0.0786	R(length)=45.6917
Excited State 11:	Singlet-B	4.9442 eV	250.77 nm	f=0.0100	R(length)=13.8455
Excited State 12:	Singlet-A	4.9746 eV	249.23 nm	f=0.0098	R(length)=-11.8700
Excited State 13:	Singlet-B	5.0885 eV	243.65 nm	f=0.0026	R(length)=-40.6774
Excited State 14:	Singlet-A	5.0904 eV	243.56 nm	f=0.0016	R(length)=10.2635
Excited State 15:	Singlet-B	5.1120 eV	242.54 nm	f=0.1903	R(length)=1.2955
Excited State 16:	Singlet-A	5.1674 eV	239.94 nm	f=0.2145	R(length)=112.1286
Excited State 17:	Singlet-A	5.2040 eV	238.25 nm	f=0.1807	R(length)=142.1771
Excited State 18:	Singlet-B	5.2324 eV	236.95 nm	f=0.0289	R(length)=170.6553
Excited State 19:	Singlet-B	5.2757 eV	235.01 nm	f=0.1711	R(length)=-221.8370
Excited State 20:	Singlet-A	5.2769 eV	234.96 nm	f=0.0084	R(length)=18.6719
Excited State 21:	Singlet-A	5.3153 eV	233.26 nm	f=0.0105	R(length)=30.4869
Excited State 22:	Singlet-B	5.3532 eV	231.61 nm	f=0.1196	R(length)=26.3708
Excited State 23:	Singlet-B	5.4612 eV	227.03 nm	f=0.0340	R(length)=-121.6725
Excited State 24:	Singlet-A	5.4874 eV	225.94 nm	f=0.0106	R(length)=6.8127
Conformer 8					
Excited State 1:	Singlet-B	3.4587 eV	358.47 nm	f=0.8929	R(length)=1890.1689
Excited State 2:	Singlet-A	3.5156 eV	352.67 nm	f=1.4311	R(length)=-1889.9116
Excited State 3:	Singlet-B	3.9561 eV	313.40 nm	f=0.2511	R(length)=57.3604
Excited State 4:	Singlet-A	4.0036 eV	309.68 nm	f=0.0039	R(length)=-17.5723
Excited State 5:	Singlet-B	4.1848 eV	296.27 nm	f=0.1421	R(length)=10.7714
Excited State 6:	Singlet-A	4.3116 eV	287.56 nm	f=0.0135	R(length)=-34.7799
Excited State 7:	Singlet-B	4.5207 eV	274.26 nm	f=0.0334	R(length)=-6.8202
Excited State 8:	Singlet-A	4.5904 eV	270.09 nm	f=0.0013	R(length)=2.1857
Excited State 9:	Singlet-B	4.7903 eV	258.82 nm	f=0.1284	R(length)=104.9515
Excited State 10:	Singlet-A	4.8432 eV	255.99 nm	f=0.0772	R(length)=-97.7180
Excited State 11:	Singlet-B	4.9414 eV	250.91 nm	f=0.0089	R(length)=21.3803
Excited State 12:	Singlet-A	4.9688 eV	249.52 nm	f=0.0130	R(length)=22.8391
Excited State 13:	Singlet-B	5.0822 eV	243.96 nm	f=0.0119	R(length)=19.7231
Excited State 14:	Singlet-A	5.0911 eV	243.53 nm	f=0.0009	R(length)=-5.8075
Excited State 15:	Singlet-B	5.1104 eV	242.61 nm	f=0.1866	R(length)=311.9433
Excited State 16:	Singlet-A	5.1632 eV	240.13 nm	f=0.1445	R(length)=-351.4557
Excited State 17:	Singlet-A	5.1986 eV	238.50 nm	f=0.2268	R(length)=-118.1473
Excited State 18:	Singlet-B	5.2227 eV	237.40 nm	f=0.0014	R(length)=38.6693
Excited State 19:	Singlet-A	5.2760 eV	235.00 nm	f=0.0228	R(length)=243.4917
Excited State 20:	Singlet-B	5.2775 eV	234.93 nm	f=0.2387	R(length)=-59.7753
Excited State 21:	Singlet-A	5.3119 eV	233.41 nm	f=0.0000	R(length)=0.4817
Excited State 22:	Singlet-B	5.3540 eV	231.57 nm	f=0.0778	R(length)=-79.1999
Excited State 23:	Singlet-B	5.4405 eV	227.89 nm	f=0.0420	R(length)=109.8526
Excited State 24:	Singlet-A	5.4579 eV	227.17 nm	f=0.0125	R(length)=-10.8524

^{1}H NMR and ^{13}C{^{1}H} NMR spectra

(*R*)-6,6'-Dibromo-[1,1'-binaphthalene]-2,2'-diol ((a*R*)-2): ^{1}H NMR (400 MHz, $CDCl_3$)

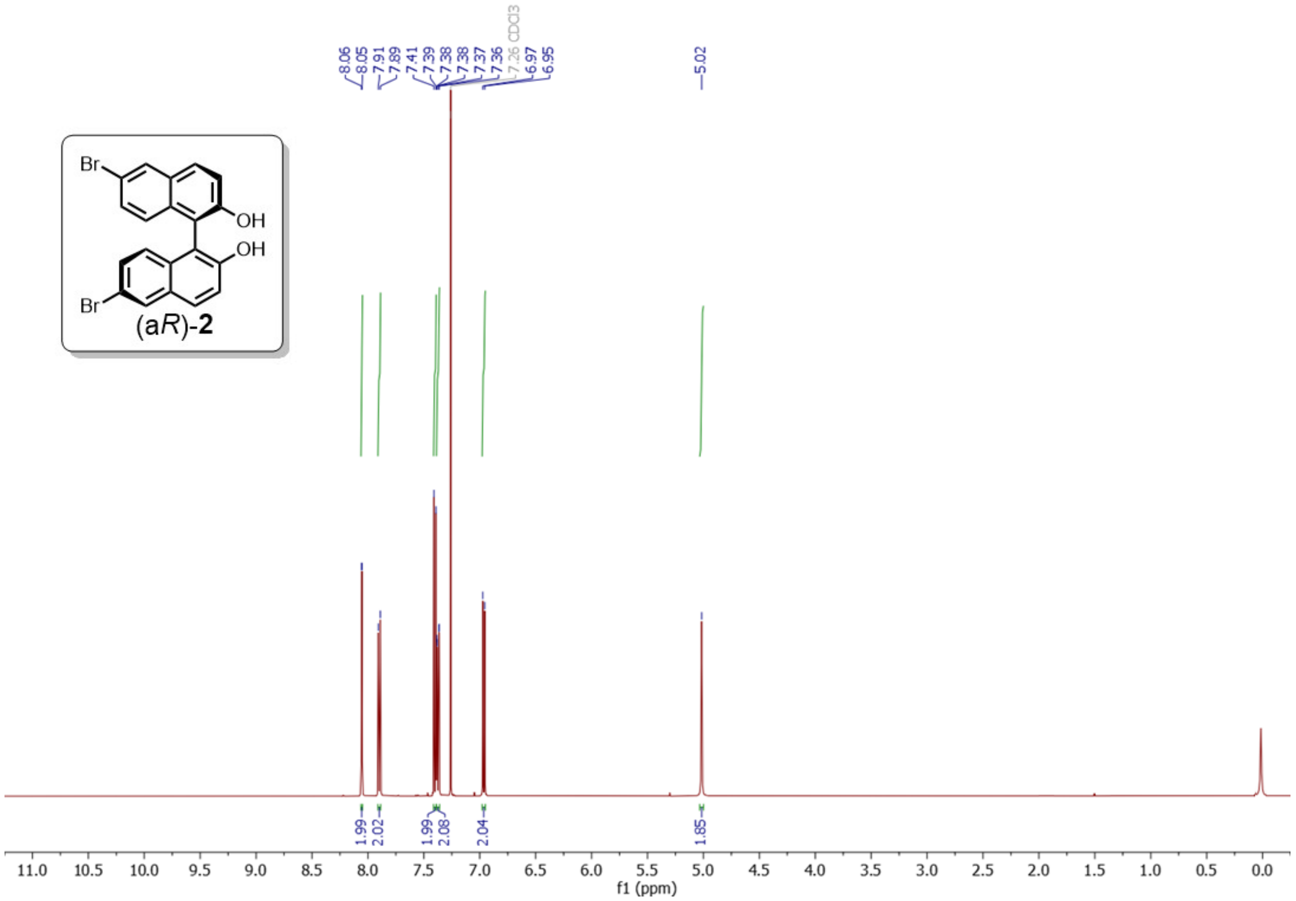

(*R*)-6,6'-Dibromo-[1,1'-binaphthalene]-2,2'-diol ((a*R*)-2): $^{13}C\{^{1}H\}$ NMR (100 MHz, $CDCl_3$)

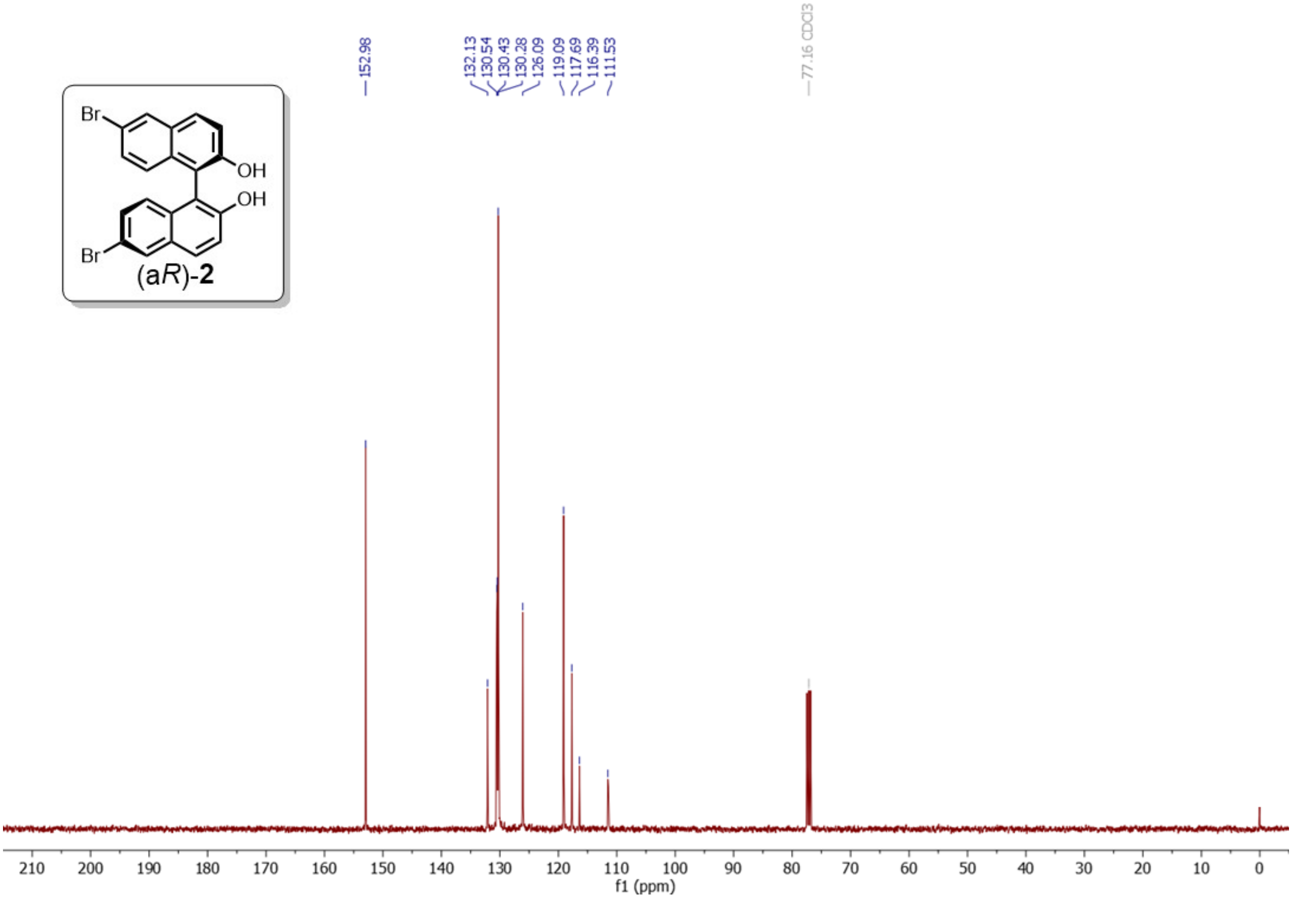

(*R*)-6,6'-Di([2,2'-bithiophen]-5-yl)-[1,1'-binaphthalene]-2,2'-diol ((a*R*)-3): ^{1}H NMR (400 MHz, $CDCl_3$)

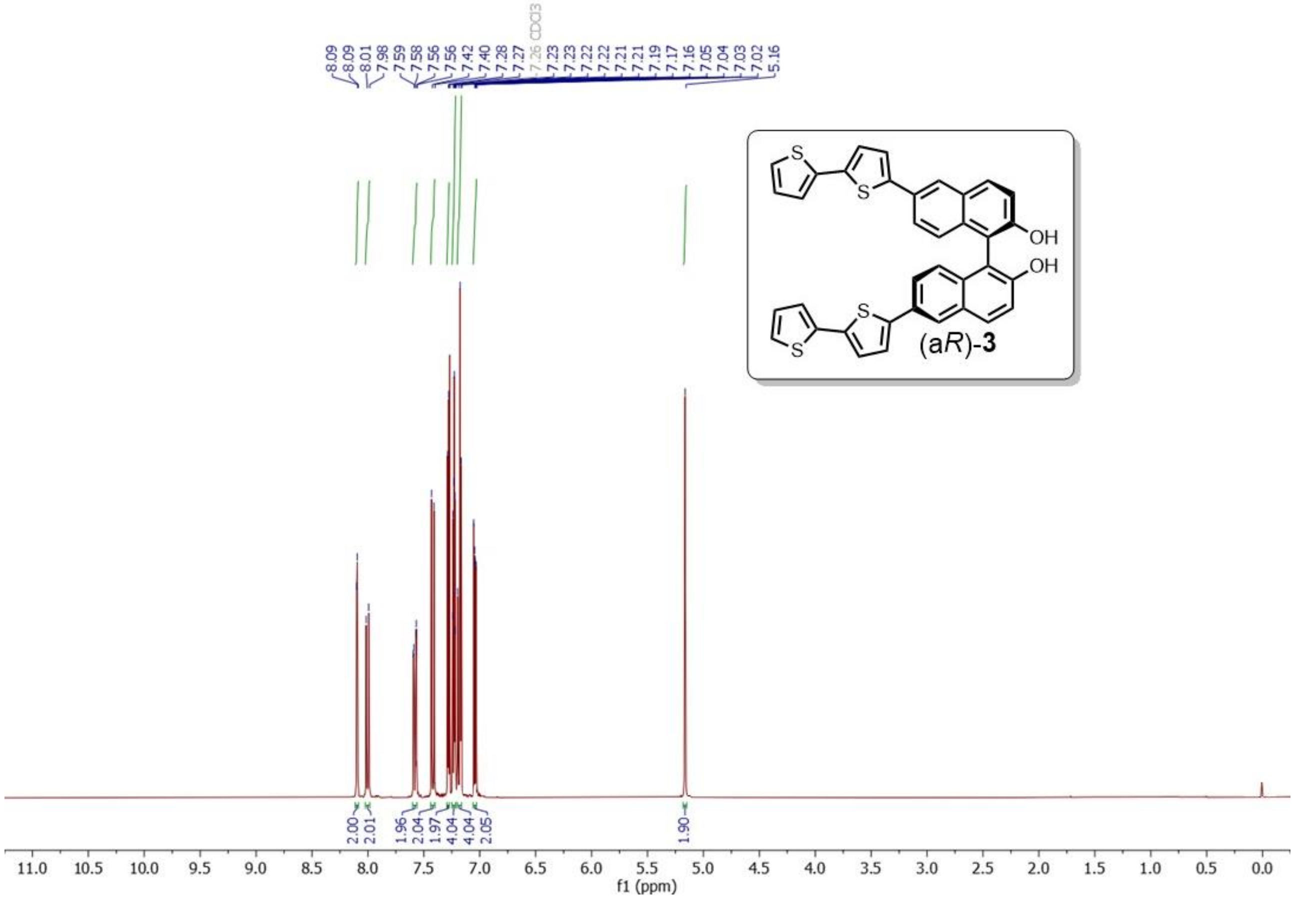

(*R*)-6,6'-Di([2,2'-bithiophen]-5-yl)-[1,1'-binaphthalene]-2,2'-diol ((a*R*)-3): $^{13}C\{^{1}H\}$ NMR (100 MHz, $CDCl_3$)

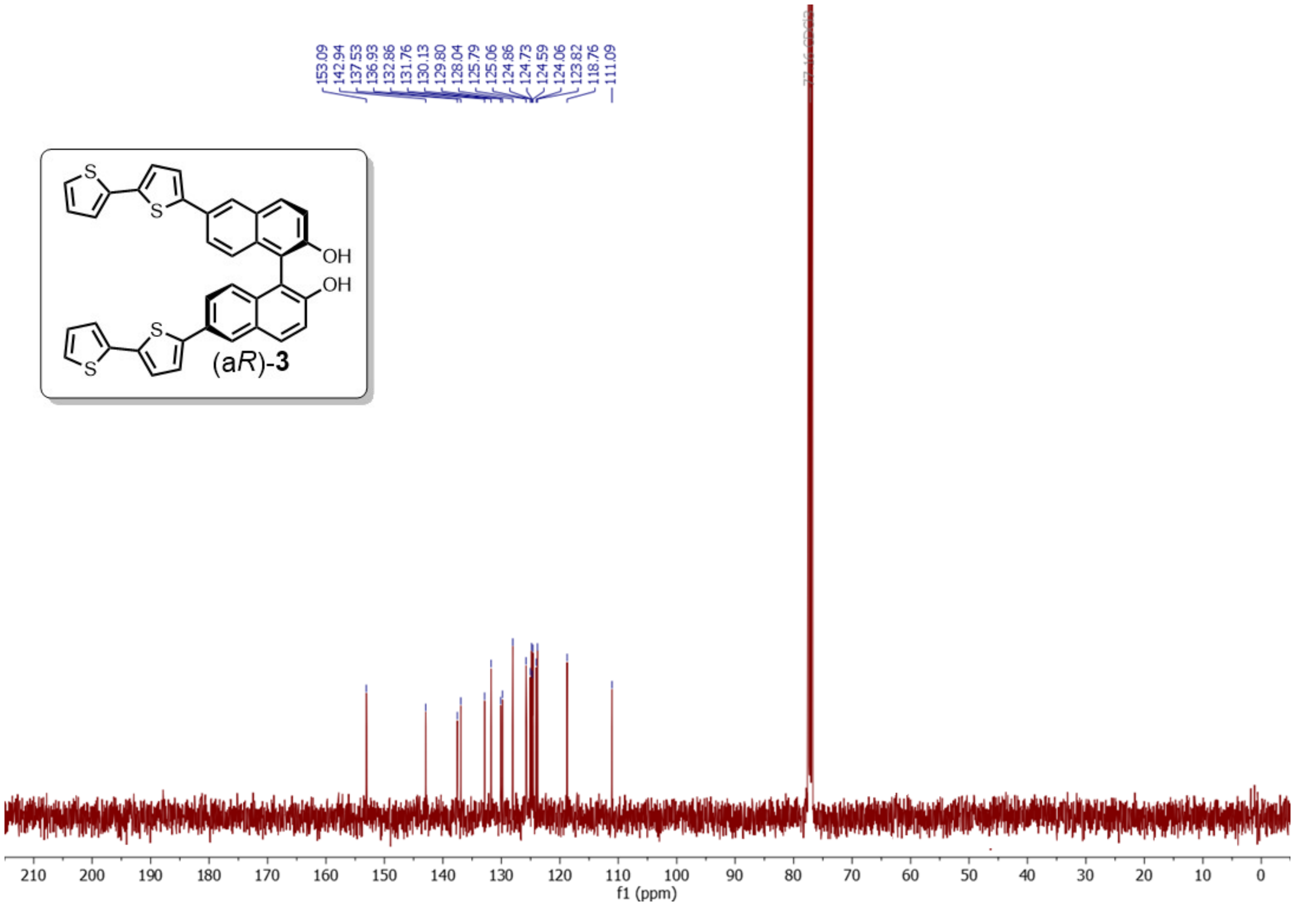

(*R*)-Dinaphtho[2,1-*d*:1',2'-*f*][1,3]dioxepine ((a*R*)-4): ^{1}H NMR (400 MHz, $CDCl_3$)

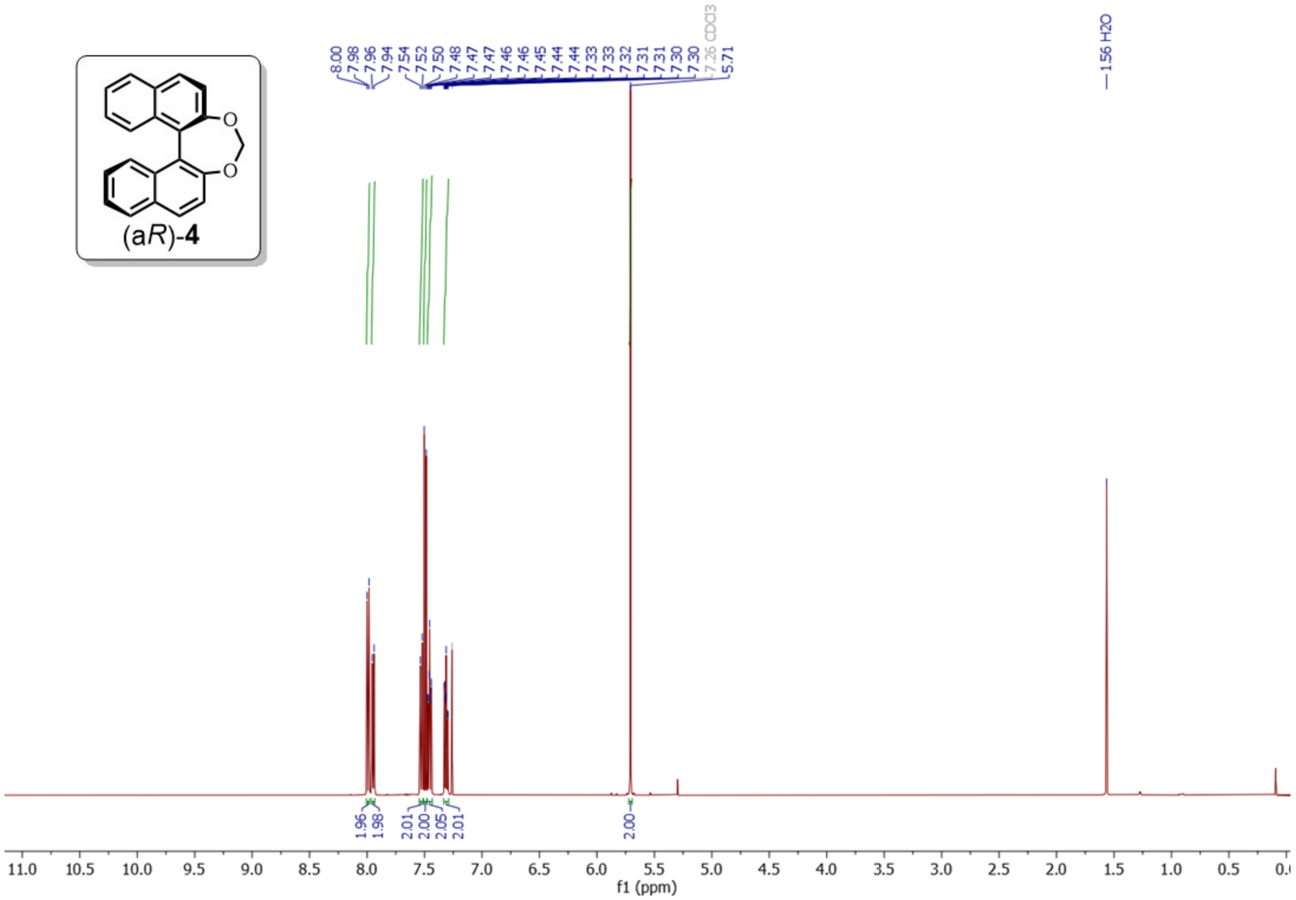

(*R*)-Dinaphtho[2,1-*d*:1',2'-*f*][1,3]dioxepine ((a*R*)-4): $^{13}C\{^{1}H\}$ NMR (100 MHz, $CDCl_3$)

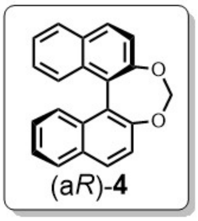

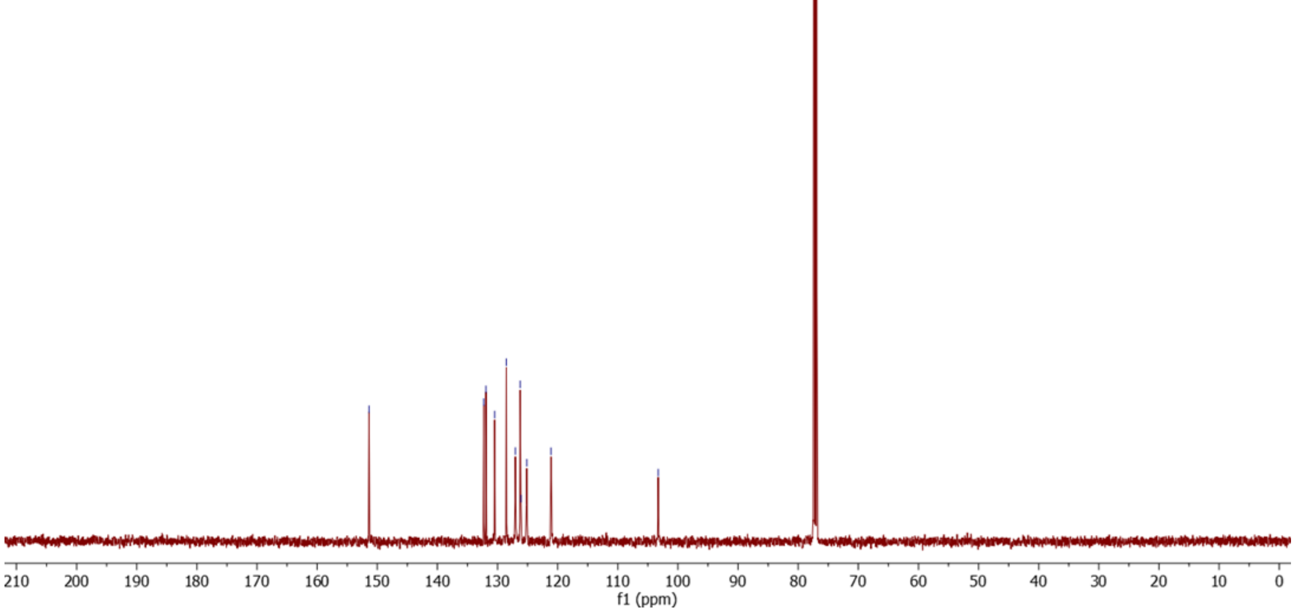

(*R*)-9,14-Dibromodinaphtho[2,1-*d*:1',2'-*f*][1,3]dioxepine ((a*R*)-5): ^{1}H NMR (400 MHz, $CDCl_3$)

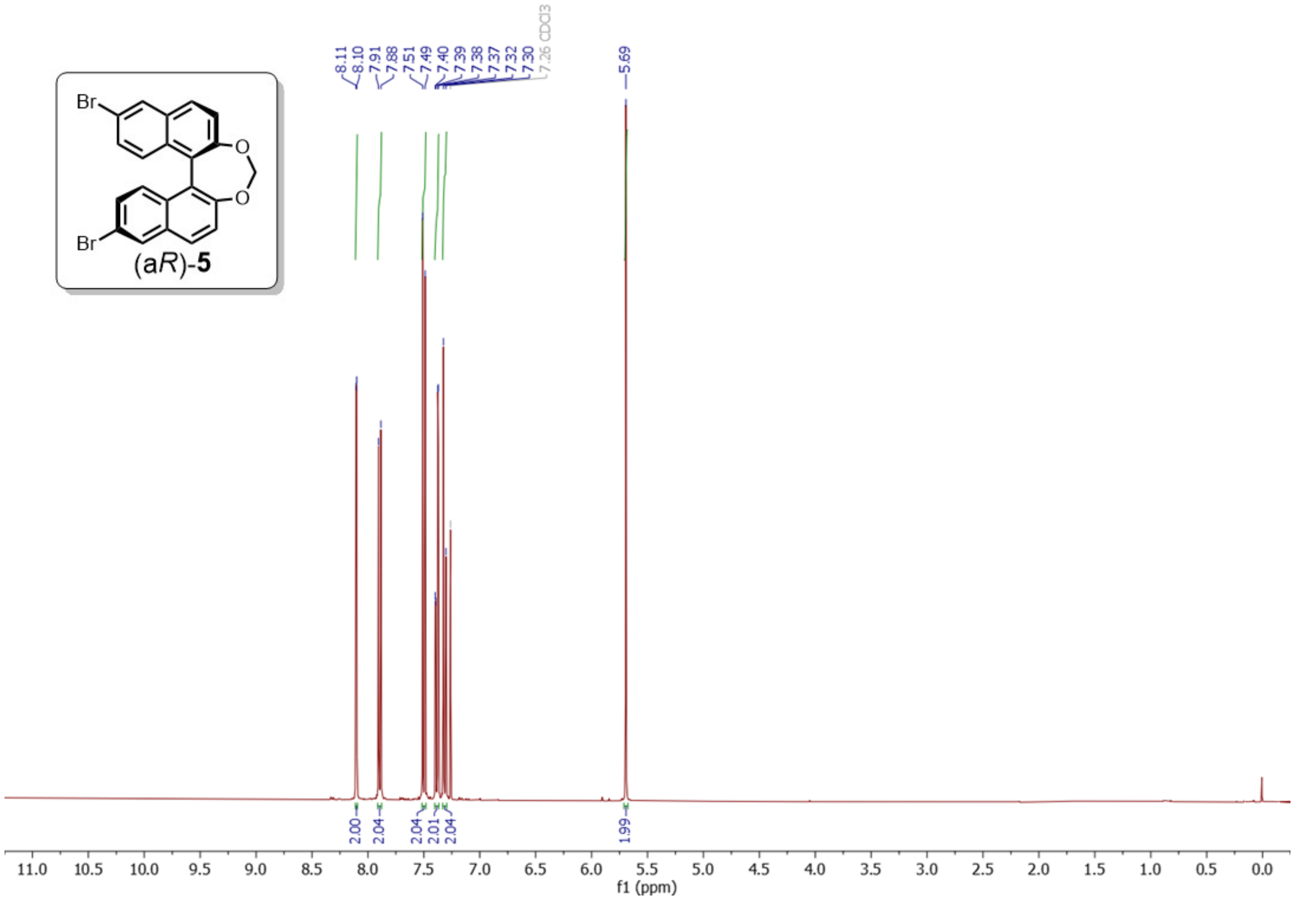

(*R*)-9,14-Dibromodinaphtho[2,1-*d*:1',2'-*f*][1,3]dioxepine ((a*R*)-5): $^{13}C\{^{1}H\}$ NMR (100 MHz, $CDCl_3$)

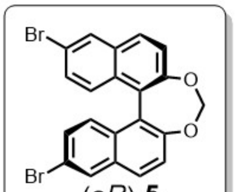

(a*R*)-**5**

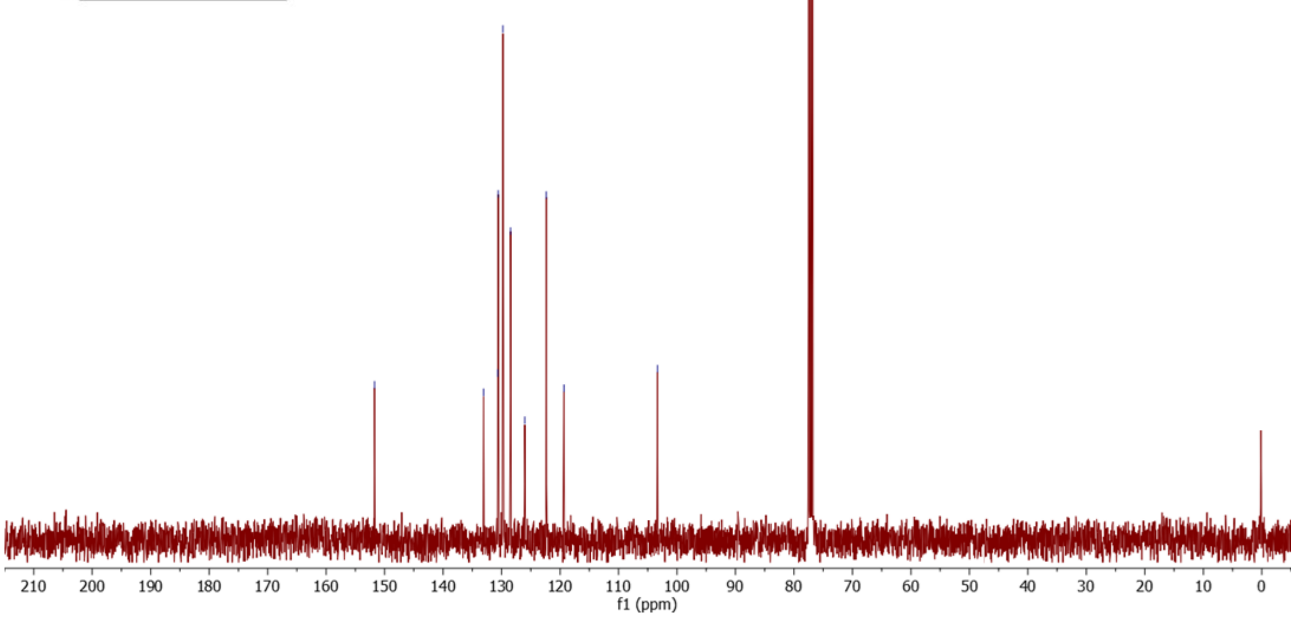

(*R*)-9,14-Di([2,2'-bithiophen]-5-yl)dinaphtho[2,1-*d*:1',2'-*f*][1,3]dioxepine ((a*R*)-6): ^{1}H NMR (400 MHz, $CDCl_3$)

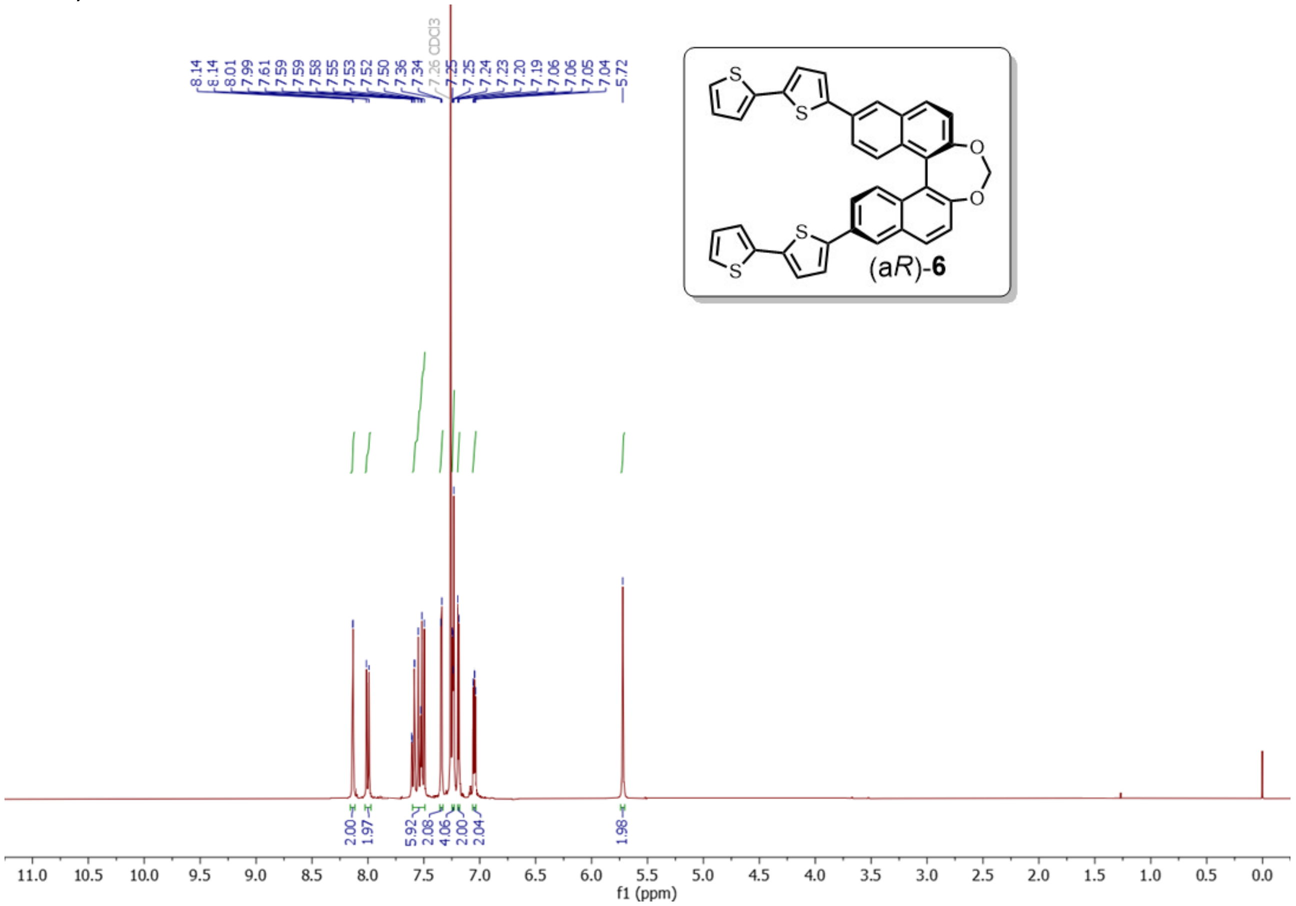

(*R*)-9,14-Di([2,2'-bithiophen]-5-yl)dinaphtho[2,1-*d*:1',2'-*f*][1,3]dioxepine ((a*R*)-6): $^{13}C\{^1H\}$ NMR (100 MHz, $CDCl_3$)

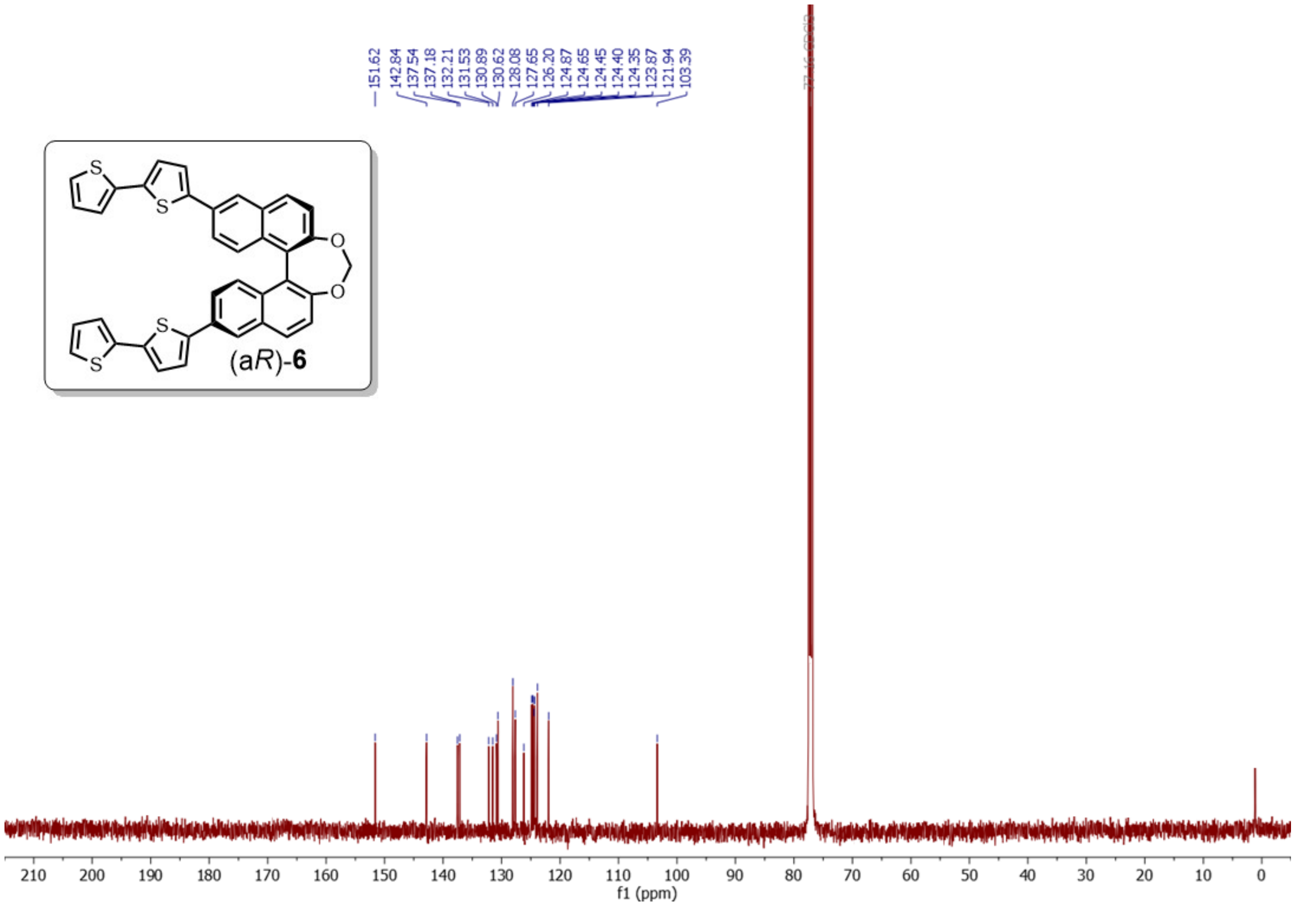

4,7-Di(thiophen-2-yl)benzo[*c*][1,2,5]thiadiazole (7): ^1H NMR (400 MHz, $CDCl_3$)

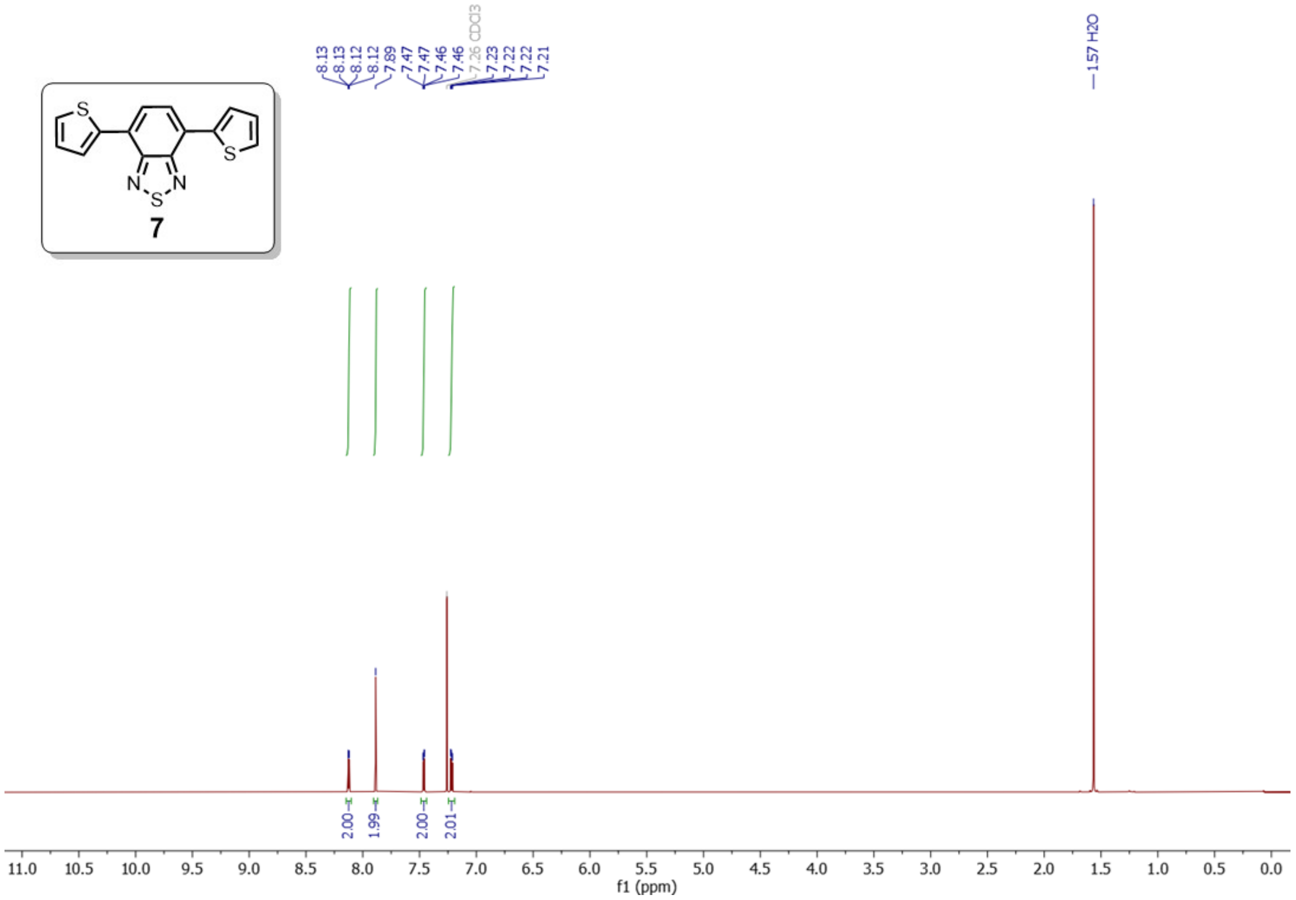

4,7-Di(thiophen-2-yl)benzo[*c*][1,2,5]thiadiazole (7): $^{13}C\{^{1}H\}$ NMR (100 MHz, $CDCl_3$)

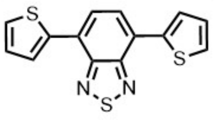

7

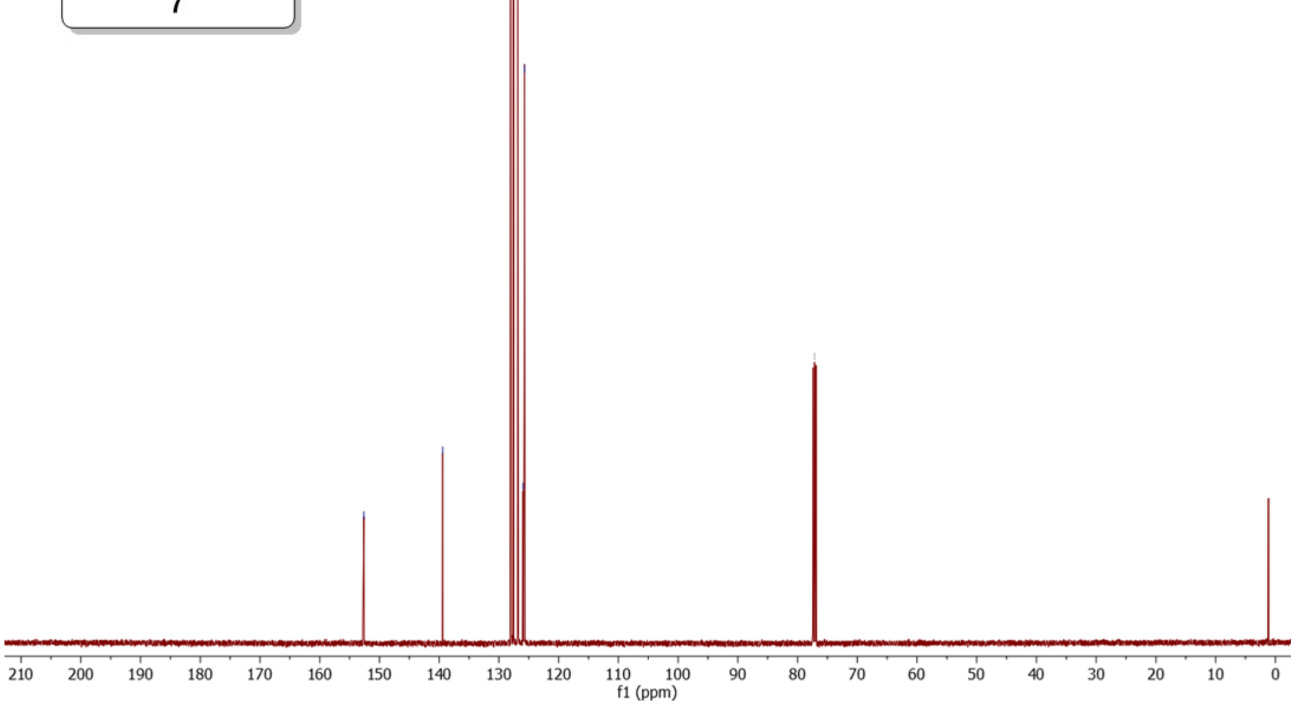

Cartesian Coordinates

Coordinates for geometries optimized at B3LYP-D3BJ/6-311+G(d,p)/PCM level

(*R*)-[1,1'-Binaphthalene]-2,2'-diol ((a*R*)-**1**):

Energy –921.391377 h, no imaginary frequencies

Symbol	X	Y	Z
H	2.4850150	2.7551810	2.0864310
C	1.6709740	2.4790700	1.4267410
H	1.3868450	4.4378080	0.6379190
C	1.0599560	3.4039140	0.6225770
C	0.2066380	0.7160570	0.5823960
C	-0.0048470	3.0354870	-0.2420150
C	1.2413770	1.1303520	1.4042140
C	-0.4367160	1.6719470	-0.2639930
C	-0.6449290	3.9807040	-1.0827190
H	-1.8288050	0.2811500	-1.1643710
C	-1.6709740	3.6013750	-1.9172630
H	-0.3075740	5.0113960	-1.0547710
H	-2.1540730	4.3310760	-2.5568040
C	-2.0964660	2.2534550	-1.9424870
H	-2.9040790	1.9596410	-2.6036460
C	-1.4962520	1.3117570	-1.1374630
C	-1.2413770	-1.1303520	1.4042140
C	-0.2066380	-0.7160570	0.5823960
C	-1.0599560	-3.4039140	0.6225770
C	0.4367160	-1.6719470	-0.2639930
C	-1.6709740	-2.4790700	1.4267410
C	0.0048470	-3.0354870	-0.2420150
C	1.4962520	-1.3117570	-1.1374630
H	-2.4850150	-2.7551810	2.0864310
H	0.3075740	-5.0113960	-1.0547710
H	-1.3868450	-4.4378080	0.6379190
C	2.0964660	-2.2534550	-1.9424870
H	1.8288050	-0.2811500	-1.1643710
H	2.9040790	-1.9596410	-2.6036460
C	1.6709740	-3.6013750	-1.9172630
H	2.1540730	-4.3310760	-2.5568040
C	0.6449290	-3.9807040	-1.0827190
O	-1.9054950	-0.2727770	2.2280950
H	-1.5323410	0.6183670	2.1305500
O	1.9054950	0.2727770	2.2280950
H	1.5323410	-0.6183670	2.1305500

(*R*)-6,6'-Dibromo-[1,1'-binaphthalene]-2,2'-diol ((a*R*)-**2**):

Energy –6068.489681 h, no imaginary frequencies

Symbol	X	Y	Z
H	2.5835180	2.6337650	3.1648460
C	1.7792650	2.3943160	2.4795420
H	1.6395770	4.3534870	1.6531420
C	1.2547810	3.3400770	1.6408710

C	0.2479130	0.7030790	1.6066800
C	0.2041450	3.0155010	0.7419710
C	1.2755220	1.0710510	2.4598650
C	-0.3056210	1.6807690	0.7243880
C	-0.3402450	3.9873440	-0.1344980
H	-1.7472740	0.3623170	-0.2102870
C	-1.3506160	3.6336120	-0.9914160
H	0.0542900	4.9954430	-0.1114910
C	-1.8689740	2.3228890	-1.0285320
H	-2.6662200	2.0685810	-1.7149710
C	-1.3506160	1.3695330	-0.1832050
C	-1.2755220	-1.0710510	2.4598650
C	-0.2479130	-0.7030790	1.6066800
C	-1.2547810	-3.3400770	1.6408710
C	0.3056210	-1.6807690	0.7243880
C	-1.7792650	-2.3943160	2.4795420
C	-0.2041450	-3.0155010	0.7419710
C	1.3506160	-1.3695330	-0.1832050
H	-2.5835180	-2.6337650	3.1648460
H	-0.0542900	-4.9954430	-0.1114910
H	-1.6395770	-4.3534870	1.6531420
C	1.8689740	-2.3228890	-1.0285320
H	1.7472740	-0.3623170	-0.2102870
H	2.6662200	-2.0685810	-1.7149710
C	1.3506160	-3.6336120	-0.9914160
C	0.3402450	-3.9873440	-0.1344980
O	-1.8635310	-0.1937070	3.3173570
H	-1.4583750	0.6825570	3.2123270
O	1.8635310	0.1937070	3.3173570
H	1.4583750	-0.6825570	3.2123270
Br	2.0910940	-4.9503770	-2.1836120
Br	-2.0910940	4.9503770	-2.1836120

(*R*)-6,6’-Di([2,2’-bithiophen]-5-yl)-[1,1’-binaphthalene]-2,2’-diol ((a*R*)-**3**):

Conformer 1, Energy –3129.009274 h, no imaginary frequencies

Symbol	X	Y	Z
H	1.3611180	4.7883850	1.4787770
C	0.7209340	3.9143310	1.4255020
C	-0.9195050	1.6543880	1.3716620
C	-0.3267890	3.8613830	0.5207200
C	0.9866910	2.8510700	2.3177240
C	0.1524600	1.6915220	2.3024100
C	-1.1485940	2.6969160	0.5107420
H	-1.5584580	0.7805060	1.3379190
H	-1.9628840	2.6271710	-0.1997000
C	0.4201030	0.6155110	3.1997110
C	1.4940400	0.7152980	4.0707830
C	2.3186320	1.8656060	4.0940890
C	2.0680310	2.9047460	3.2381900
H	3.1455610	1.8985450	4.7935170
H	2.7002850	3.7855990	3.2522200
C	-0.4201030	-0.6155110	3.1997110
C	-2.0680310	-2.9047460	3.2381900

C	-0.1524600	-1.6915220	2.3024100
C	-1.4940400	-0.7152980	4.0707830
C	-2.3186320	-1.8656060	4.0940890
C	-0.9866910	-2.8510700	2.3177240
H	-2.7002850	-3.7855990	3.2522200
H	-3.1455610	-1.8985450	4.7935170
C	-0.7209340	-3.9143310	1.4255020
C	0.3267890	-3.8613830	0.5207200
C	1.1485940	-2.6969160	0.5107420
C	0.9195050	-1.6543880	1.3716620
H	-1.3611180	-4.7883850	1.4787770
H	1.9628840	-2.6271710	-0.1997000
H	1.5584580	-0.7805060	1.3379190
O	1.8205660	-0.2786340	4.9422940
O	-1.8205660	0.2786340	4.9422940
C	-0.6064010	4.9526050	-0.4106170
S	0.6064010	6.1674860	-0.7822310
C	-0.4840360	7.0200810	-1.8600050
C	-1.6953900	6.3698210	-1.9249780
C	-1.7617120	5.2089080	-1.1137570
H	-2.5253150	6.7276640	-2.5209280
H	-2.6485630	4.5950360	-1.0331090
C	-0.0540680	8.2209800	-2.5400290
C	0.9783640	9.0754730	-2.2231970
C	1.1101200	10.1653670	-3.1296700
C	0.1822890	10.1405160	-4.1324030
S	-0.8715910	8.7677520	-3.9946790
H	1.6121720	8.9379610	-1.3564810
H	1.8596050	10.9400770	-3.0336960
H	0.0450820	10.8392510	-4.9446220
C	0.6064010	-4.9526050	-0.4106170
C	1.7617120	-5.2089080	-1.1137570
C	1.6953900	-6.3698210	-1.9249780
C	0.4840360	-7.0200810	-1.8600050
S	-0.6064010	-6.1674860	-0.7822310
H	2.6485630	-4.5950360	-1.0331090
H	2.5253150	-6.7276640	-2.5209280
C	0.0540680	-8.2209800	-2.5400290
S	0.8715910	-8.7677520	-3.9946790
C	-0.1822890	-10.1405160	-4.1324030
C	-1.1101200	-10.1653670	-3.1296700
C	-0.9783640	-9.0754730	-2.2231970
H	-0.0450820	-10.8392510	-4.9446220
H	-1.8596050	-10.9400770	-3.0336960
H	-1.6121720	-8.9379610	-1.3564810
H	1.2143590	-1.0268560	4.8170590
H	-1.2143590	1.0268560	4.8170590

Conformer 2, Energy –3129.009258 h, no imaginary frequencies

Symbol	X	Y	Z
H	1.6402880	4.6820180	1.2149530
C	0.9352200	3.8581310	1.1871120
C	-0.8156440	1.6821680	1.1328180
C	-0.1033280	3.8488400	0.2704360

C	1.1311310	2.7954530	2.0979440
C	0.2448480	1.6752200	2.0771860
C	-0.9858290	2.7296270	0.2640420
H	-1.5048630	0.8470760	1.1079350
H	-1.8165880	2.7121830	-0.4306470
C	0.4484610	0.5952610	2.9866070
C	1.5129640	0.6521760	3.8730760
C	2.3902860	1.7628110	3.9001990
C	2.2016240	2.8051270	3.0325020
H	3.2078430	1.7622980	4.6112980
H	2.8744280	3.6553940	3.0487990
C	-0.4484610	-0.5952610	2.9866070
C	-2.2016240	-2.8051270	3.0325020
C	-0.2448480	-1.6752200	2.0771860
C	-1.5129640	-0.6521760	3.8730760
C	-2.3902860	-1.7628110	3.9001990
C	-1.1311310	-2.7954530	2.0979440
H	-2.8744280	-3.6553940	3.0487990
H	-3.2078430	-1.7622980	4.6112980
C	-0.9352200	-3.8581310	1.1871120
C	0.1033280	-3.8488400	0.2704360
C	0.9858290	-2.7296270	0.2640420
C	0.8156440	-1.6821680	1.1328180
H	-1.6402880	-4.6820180	1.2149530
H	1.8165880	-2.7121830	-0.4306470
H	1.5048630	-0.8470760	1.1079350
O	1.7804540	-0.3491120	4.7560380
O	-1.7804540	0.3491120	4.7560380
C	-0.3121920	4.9402070	-0.6792750
S	0.3577670	6.5383660	-0.3948190
C	-0.2951060	7.1517390	-1.9035030
C	-1.0005930	6.1646170	-2.5535780
C	-1.0117990	4.9257140	-1.8643130
H	-1.4795860	6.3156910	-3.5125540
H	-1.4992520	4.0398700	-2.2483610
C	-0.0692370	8.5239660	-2.2984680
C	0.9033580	9.4012720	-1.8732090
C	0.8156440	10.6842610	-2.4841300
C	-0.2188900	10.7833350	-3.3711190
S	-1.1214530	9.3036000	-3.4685830
H	1.6706070	9.1305960	-1.1592920
H	1.5002090	11.4965690	-2.2778290
H	-0.5119560	11.6283640	-3.9766770
C	0.3121920	-4.9402070	-0.6792750
C	1.0117990	-4.9257140	-1.8643130
C	1.0005930	-6.1646170	-2.5535780
C	0.2951060	-7.1517390	-1.9035030
S	-0.3577670	-6.5383660	-0.3948190
H	1.4992520	-4.0398700	-2.2483610
H	1.4795860	-6.3156910	-3.5125540
C	0.0692370	-8.5239660	-2.2984680
S	1.1214530	-9.3036000	-3.4685830
C	0.2188900	-10.7833350	-3.3711190
C	-0.8156440	-10.6842610	-2.4841300
C	-0.9033580	-9.4012720	-1.8732090
H	0.5119560	-11.6283640	-3.9766770

H	-1.5002090	-11.4965690	-2.2778290
H	-1.6706070	-9.1305960	-1.1592920
H	1.1460570	-1.0721530	4.6225360
H	-1.1460570	1.0721530	4.6225360

Conformer 3, Energy –3129.009100 h, no imaginary frequencies

Symbol	X	Y	Z
H	1.4076650	4.7740680	1.4400290
C	0.7582180	3.9068020	1.3872010
C	-0.9029720	1.6620980	1.3333180
C	-0.2907860	3.8642700	0.4833260
C	1.0141040	2.8411360	2.2794500
C	0.1689460	1.6895090	2.2644060
C	-1.1228500	2.7070590	0.4730430
H	-1.5497410	0.7940090	1.2992110
H	-1.9382080	2.6448180	-0.2367930
C	0.4258200	0.6115660	3.1624390
C	1.4996850	0.7020000	4.0346200
C	2.3352880	1.8443530	4.0575530
C	2.0954900	2.8850230	3.2004040
H	3.1619190	1.8699570	4.7576450
H	2.7361900	3.7597620	3.2141470
C	-0.4258200	-0.6115660	3.1624390
C	-2.0954900	-2.8850230	3.2004040
C	-0.1689460	-1.6895090	2.2644060
C	-1.4996850	-0.7020000	4.0346200
C	-2.3352880	-1.8443530	4.0575530
C	-1.0141040	-2.8411360	2.2794500
H	-2.7361900	-3.7597620	3.2141470
H	-3.1619190	-1.8699570	4.7576450
C	-0.7582180	-3.9068020	1.3872010
C	0.2907860	-3.8642700	0.4833260
C	1.1228500	-2.7070590	0.4730430
C	0.9029720	-1.6620980	1.3333180
H	-1.4076650	-4.7740680	1.4400290
H	1.9382080	-2.6448180	-0.2367930
H	1.5497410	-0.7940090	1.2992110
O	1.8156180	-0.2941350	4.9074610
O	-1.8156180	0.2941350	4.9074610
C	-0.5645170	4.9603340	-0.4440620
S	0.6477360	6.1817120	-0.7981970
C	-0.4258200	7.0200910	-1.9052230
C	-1.6387310	6.3725710	-1.9732290
C	-1.7149920	5.2182270	-1.1540860
H	-2.4547460	6.7102770	-2.5993450
H	-2.6004930	4.6010060	-1.0858710
C	0.0107140	8.2147750	-2.5915680
C	1.2839140	8.7227460	-2.7256380
C	1.3370780	9.9301550	-3.4778320
C	0.1096260	10.3401230	-3.9157670
S	-1.1467040	9.2560740	-3.4052750
H	2.1590950	8.2405160	-2.3093560
H	2.2525290	10.4671490	-3.6891800
H	-0.1432780	11.2085010	-4.5062380

C	0.5645170	-4.9603340	-0.4440620
C	1.7149920	-5.2182270	-1.1540860
C	1.6387310	-6.3725710	-1.9732290
C	0.4258200	-7.0200910	-1.9052230
S	-0.6477360	-6.1817120	-0.7981970
H	2.6004930	-4.6010060	-1.0858710
H	2.4547460	-6.7102770	-2.5993450
C	-0.0107140	-8.2147750	-2.5915680
S	1.1467040	-9.2560740	-3.4052750
C	-0.1096260	-10.3401230	-3.9157670
C	-1.3370780	-9.9301550	-3.4778320
C	-1.2839140	-8.7227460	-2.7256380
H	0.1432780	-11.2085010	-4.5062380
H	-2.2525290	-10.4671490	-3.6891800
H	-2.1590950	-8.2405160	-2.3093560
H	1.2030590	-1.0370490	4.7814810
H	-1.2030590	1.0370490	4.7814810

Conformer 4, Energy –3129.009069 h, no imaginary frequencies

Symbol	X	Y	Z
H	1.5833750	4.6999710	1.2579650
C	0.8864410	3.8690190	1.2303480
C	-0.8452410	1.6780770	1.1870830
C	-0.1604900	3.8535540	0.3230210
C	1.0995420	2.8065970	2.1376830
C	0.2228780	1.6788160	2.1229320
C	-1.0320920	2.7255240	0.3223780
H	-1.5262980	0.8362440	1.1648570
H	-1.8662460	2.6989400	-0.3673680
C	0.4423890	0.5997390	3.0293370
C	1.5122760	0.6650430	3.9086800
C	2.3800180	1.7832250	3.9304700
C	2.1765880	2.8244820	3.0646170
H	3.2024720	1.7894210	4.6358950
H	2.8424790	3.6802430	3.0769090
C	-0.4423890	-0.5997390	3.0293370
C	-2.1765880	-2.8244820	3.0646170
C	-0.2228780	-1.6788160	2.1229320
C	-1.5122760	-0.6650430	3.9086800
C	-2.3800180	-1.7832250	3.9304700
C	-1.0995420	-2.8065970	2.1376830
H	-2.8424790	-3.6802430	3.0769090
H	-3.2024720	-1.7894210	4.6358950
C	-0.8864410	-3.8690190	1.2303480
C	0.1604900	-3.8535540	0.3230210
C	1.0320920	-2.7255240	0.3223780
C	0.8452410	-1.6780770	1.1870830
H	-1.5833750	-4.6999710	1.2579650
H	1.8662460	-2.6989400	-0.3673680
H	1.5262980	-0.8362440	1.1648570
O	1.7942110	-0.3348530	4.7887810
O	-1.7942110	0.3348530	4.7887810
C	-0.3900630	4.9438400	-0.6226000
S	0.3900630	6.5058510	-0.4260700

C	-0.3839600	7.1475430	-1.8652820
C	-1.1889070	6.1891360	-2.4387530
C	-1.1913900	4.9552650	-1.7416600
H	-1.7691390	6.3635410	-3.3357790
H	-1.7647480	4.0969070	-2.0643200
C	-0.1353390	8.5054600	-2.2930570
C	0.5046850	9.5241060	-1.6220200
C	0.5623480	10.7410730	-2.3581830
C	-0.0315290	10.6508100	-3.5851320
S	-0.6705190	9.0621500	-3.8715310
H	0.9134270	9.4110090	-0.6260430
H	1.0224240	11.6471750	-1.9863440
H	-0.1387820	11.4129020	-4.3429860
C	0.3900630	-4.9438400	-0.6226000
C	1.1913900	-4.9552650	-1.7416600
C	1.1889070	-6.1891360	-2.4387530
C	0.3839600	-7.1475430	-1.8652820
S	-0.3900630	-6.5058510	-0.4260700
H	1.7647480	-4.0969070	-2.0643200
H	1.7691390	-6.3635410	-3.3357790
C	0.1353390	-8.5054600	-2.2930570
S	0.6705190	-9.0621500	-3.8715310
C	0.0315290	-10.6508100	-3.5851320
C	-0.5623480	-10.7410730	-2.3581830
C	-0.5046850	-9.5241060	-1.6220200
H	0.1387820	-11.4129020	-4.3429860
H	-1.0224240	-11.6471750	-1.9863440
H	-0.9134270	-9.4110090	-0.6260430
H	1.1645000	-1.0627210	4.6594260
H	-1.1645000	1.0627210	4.6594260

Conformer 5, Energy –3129.008686 h, no imaginary frequencies

Symbol	X	Y	Z
H	4.3669450	0.9727240	1.8174820
C	3.6051390	1.1177530	1.0588570
C	1.5869000	1.5054150	-0.8326490
C	3.6443800	0.4028350	-0.1271770
C	2.5715780	2.0406190	1.3364820
C	1.5318480	2.2451490	0.3783560
C	2.6052480	0.6204510	-1.0781710
H	0.8123330	1.6520190	-1.5754700
H	2.6253030	0.0843890	-2.0187630
C	0.4808270	3.1668200	0.6612800
C	0.4859930	3.8451900	1.8703990
C	1.5163520	3.6481750	2.8206950
C	2.5306990	2.7667110	2.5573610
H	1.4786490	4.2019290	3.7512440
H	3.3195470	2.6120740	3.2850790
C	-0.6320540	3.3900440	-0.3047320
C	-2.6926340	3.8554380	-2.1756940
C	-1.7863980	2.5508900	-0.3053650
C	-0.5459030	4.4178610	-1.2302170
C	-1.5809840	4.6540330	-2.1675990
C	-2.8327770	2.7848070	-1.2505610

H	-3.4851240	4.0328170	-2.8943140
H	-1.4676250	5.4698260	-2.8716290
C	-3.9702400	1.9478000	-1.2570430
C	-4.1070880	0.9021570	-0.3570970
C	-3.0584570	0.6790830	0.5793070
C	-1.9384370	1.4716720	0.6029610
H	-4.7376880	2.1337470	-1.9992820
H	-3.1470910	-0.1236650	1.3026390
H	-1.1592640	1.2792190	1.3299860
O	-0.4934540	4.7255130	2.2161890
O	0.5299000	5.2492980	-1.3002840
C	4.7040880	-0.5598710	-0.4211530
S	6.2114120	-0.5625920	0.4807840
C	6.8417100	-1.9107930	-0.4491350
C	5.9197150	-2.3128760	-1.3889740
C	4.7219430	-1.5547030	-1.3721650
H	6.0975570	-3.1269510	-2.0801650
H	3.8928260	-1.7457750	-2.0398040
C	8.1567770	-2.4436750	-0.1740050
C	9.1823360	-1.8901920	0.5601440
C	10.3454460	-2.7090720	0.6170140
C	10.2066830	-3.8812630	-0.0708750
S	8.6361690	-4.0136950	-0.7988670
H	9.1127230	-0.9195440	1.0343900
H	11.2498150	-2.4332890	1.1434320
H	10.9234450	-4.6788540	-0.2005670
C	-5.3016040	0.0585220	-0.3688650
C	-6.5435790	0.3276960	-0.8968430
C	-7.4726110	-0.7320770	-0.7486770
C	-6.9582050	-1.8325070	-0.0997550
S	-5.2841220	-1.5486540	0.3395050
H	-6.7880340	1.2685780	-1.3714440
H	-8.4907790	-0.6848630	-1.1135920
C	-7.6066010	-3.0804170	0.2352330
S	-9.3567190	-3.2226220	0.1859860
C	-9.2869650	-4.8863940	0.6775010
C	-7.9998690	-5.2985230	0.8771700
C	-7.0429740	-4.2745800	0.6263680
H	-10.2023690	-5.4494110	0.7851650
H	-7.7391980	-6.3022560	1.1864210
H	-5.9744040	-4.4213920	0.7185130
H	-1.1544290	4.7650770	1.5059590
H	1.1857620	4.9830640	-0.6353030

Conformer 6, Energy –3129.008482 h, no imaginary frequencies

Symbol	X	Y	Z
H	-4.2485330	1.5351120	-2.6062010
C	-3.5783070	1.4897370	-1.7556630
C	-1.8550000	1.4444030	0.4455100
C	-3.8172790	0.5929920	-0.7257710
C	-2.4771240	2.3736200	-1.7250960
C	-1.5906900	2.3619540	-0.6037700
C	-2.9271820	0.5900340	0.3849670
H	-1.1923810	1.4154860	1.3016190

H	-3.0846020	-0.1122390	1.1956460
C	-0.4770260	3.2536630	-0.5724890
C	-0.2746080	4.1170550	-1.6372620
C	-1.1549160	4.1375290	-2.7464970
C	-2.2272010	3.2874730	-2.7853440
H	-0.9566280	4.8316880	-3.5544050
H	-2.9011460	3.2999470	-3.6348100
C	0.4769340	3.2536480	0.5724650
C	2.2269540	3.2872590	2.7854460
C	1.5905910	2.3619340	0.6037360
C	0.2744610	4.1169560	1.6373000
C	1.1546740	4.1373260	2.7466070
C	2.4769450	2.3735190	1.7251220
H	2.9008270	3.2996420	3.6349700
H	0.9563130	4.8313700	3.5545940
C	3.5781510	1.4896690	1.7557060
C	3.8172550	0.5930140	0.7257640
C	2.9272250	0.5901340	-0.3850370
C	1.8550120	1.4444600	-0.4455860
H	4.2482780	1.5350510	2.6063200
H	3.0847240	-0.1120760	-1.1957590
H	1.1924580	1.4155730	-1.3017470
O	0.7741950	4.9839030	-1.6845390
O	-0.7743140	4.9838360	1.6845440
C	-4.9524580	-0.3270210	-0.7861770
S	-5.6397880	-1.0156480	0.6754960
C	-6.8397990	-1.9151470	-0.2335150
C	-6.7030510	-1.6638250	-1.5807080
C	-5.6447530	-0.7727150	-1.8886980
H	-7.3274110	-2.1270560	-2.3341250
H	-5.3822060	-0.4887110	-2.8990590
C	-7.7895270	-2.7664860	0.4474400
C	-7.7307530	-3.2850610	1.7220000
C	-8.8572940	-4.0870160	2.0605230
C	-9.7697670	-4.1798410	1.0478950
S	-9.2731460	-3.2711390	-0.3456000
H	-6.8979810	-3.1118200	2.3915630
H	-8.9785720	-4.5811630	3.0156030
H	-10.7027400	-4.7234700	1.0257690
C	4.9525320	-0.3268810	0.7861270
C	5.6453220	-0.7720160	1.8885730
C	6.7036610	-1.6630440	1.5805570
C	6.8399890	-1.9148880	0.2334170
S	5.6394470	-1.0159970	-0.6755220
H	5.3831080	-0.4876920	2.8989280
H	7.3283910	-2.1258260	2.3339420
C	7.7896790	-2.7662950	-0.4474910
S	9.2729600	-3.2715110	0.3458460
C	9.7698430	-4.1798340	-1.0478000
C	8.8576060	-4.0866680	-2.0606070
C	7.7311250	-3.2845890	-1.7221790
H	10.7027500	-4.7235720	-1.0255870
H	8.9790430	-4.5805820	-3.0157870
H	6.8985840	-3.1110260	-2.3919430
H	1.3214240	4.8690290	-0.8904470
H	-1.3216620	4.8688250	0.8905540

Conformer 7, Energy –3129.008464 h, no imaginary frequencies

Symbol	X	Y	Z
H	-1.9127180	4.5746150	-1.8559810
C	-1.1636340	3.7916740	-1.8372140
C	0.7133400	1.7210100	-1.7889300
C	-0.1241320	3.8411160	-0.9213900
C	-1.2930520	2.7236320	-2.7519200
C	-0.3418240	1.6568140	-2.7350960
C	0.8190290	2.7749930	-0.9161930
H	1.4488990	0.9266930	-1.7631080
H	1.6504170	2.7975720	-0.2207400
C	-0.4819310	0.5688680	-3.6484250
C	-1.5473670	0.5672850	-4.5348950
C	-2.4888750	1.6250250	-4.5584290
C	-2.3628950	2.6728700	-3.6870680
H	-3.3045770	1.5785030	-5.2700620
H	-3.0848460	3.4818630	-3.7002960
C	0.4819310	-0.5688680	-3.6484250
C	2.3628950	-2.6728700	-3.6870680
C	0.3418240	-1.6568140	-2.7350960
C	1.5473670	-0.5672850	-4.5348950
C	2.4888750	-1.6250250	-4.5584290
C	1.2930520	-2.7236320	-2.7519200
H	3.0848460	-3.4818630	-3.7002960
H	3.3045770	-1.5785030	-5.2700620
C	1.1636340	-3.7916740	-1.8372140
C	0.1241320	-3.8411160	-0.9213900
C	-0.8190290	-2.7749930	-0.9161930
C	-0.7133400	-1.7210100	-1.7889300
H	1.9127180	-4.5746150	-1.8559810
H	-1.6504170	-2.7975720	-0.2207400
H	-1.4488990	-0.9266930	-1.7631080
O	-1.7577670	-0.4441680	-5.4216510
O	1.7577670	0.4441680	-5.4216510
C	-0.0047790	4.9627740	0.0094140
S	0.8838920	4.8018320	1.5150310
C	0.5486280	6.4625650	1.9673960
C	-0.2213360	7.0725250	1.0020950
C	-0.5311270	6.2301240	-0.0947280
H	-0.5343810	8.1069700	1.0638260
H	-1.1051520	6.5571500	-0.9514300
C	1.0596260	7.0032680	3.2071630
C	2.0922010	6.5433260	3.9939500
C	2.3153420	7.3346940	5.1561910
C	1.4573530	8.3933560	5.2538270
S	0.3418240	8.4371720	3.9240130
H	2.6863670	5.6748040	3.7398210
H	3.0889640	7.1289290	5.8843120
H	1.4056990	9.1532880	6.0197370
C	0.0047790	-4.9627740	0.0094140
C	0.5311270	-6.2301240	-0.0947280
C	0.2213360	-7.0725250	1.0020950
C	-0.5486280	-6.4625650	1.9673960

S	-0.8838920	-4.8018320	1.5150310
H	1.1051520	-6.5571500	-0.9514300
H	0.5343810	-8.1069700	1.0638260
C	-1.0596260	-7.0032680	3.2071630
S	-0.3418240	-8.4371720	3.9240130
C	-1.4573530	-8.3933560	5.2538270
C	-2.3153420	-7.3346940	5.1561910
C	-2.0922010	-6.5433260	3.9939500
H	-1.4056990	-9.1532880	6.0197370
H	-3.0889640	-7.1289290	5.8843120
H	-2.6863670	-5.6748040	3.7398210
H	-1.0821060	-1.1295020	-5.2920730
H	1.0821060	1.1295020	-5.2920730

Conformer 8, Energy –3129.008272 h, no imaginary frequencies

Symbol	X	Y	Z
H	2.5885100	4.2569230	1.5988560
C	1.7415340	3.5823960	1.5516830
C	-0.4491140	1.8462200	1.5028440
C	0.7149840	3.8117670	0.6486350
C	1.7133600	2.4845190	2.4396210
C	0.5977400	1.5910840	2.4257950
C	-0.3907370	2.9153960	0.6445370
H	-1.3015520	1.1789420	1.4730070
H	-1.2000420	3.0654360	-0.0610580
C	0.5695610	0.4803690	3.3211830
C	1.6316380	0.2878200	4.1902560
C	2.7349800	1.1754150	4.2130840
C	2.7708450	2.2448890	3.3594080
H	3.5408520	0.9847800	4.9117200
H	3.6158260	2.9244140	3.3733610
C	-0.5695610	-0.4803690	3.3211830
C	-2.7708450	-2.2448890	3.3594080
C	-0.5977400	-1.5910840	2.4257950
C	-1.6316380	-0.2878200	4.1902560
C	-2.7349800	-1.1754150	4.2130840
C	-1.7133600	-2.4845190	2.4396210
H	-3.6158260	-2.9244140	3.3733610
H	-3.5408520	-0.9847800	4.9117200
C	-1.7415340	-3.5823960	1.5516830
C	-0.7149840	-3.8117670	0.6486350
C	0.3907370	-2.9153960	0.6445370
C	0.4491140	-1.8462200	1.5028440
H	-2.5885100	-4.2569230	1.5988560
H	1.2000420	-3.0654360	-0.0610580
H	1.3015520	-1.1789420	1.4730070
O	1.6815720	-0.7577650	5.0608410
O	-1.6815720	0.7577650	5.0608410
C	0.7758280	4.9408470	-0.2791530
S	-0.6802880	5.5941180	-1.0123550
C	0.2256360	6.8256230	-1.8721390
C	1.5693260	6.7079850	-1.5946470
C	1.8762370	5.6499070	-0.7032580
H	2.3185610	7.3669050	-2.0145170

H	2.8838780	5.4109010	-0.3905680
C	-0.4491140	7.7702910	-2.7345460
C	-1.7800660	8.1224270	-2.7650690
C	-2.0870420	9.0949460	-3.7586410
C	-0.9945470	9.4822390	-4.4818270
S	0.4400450	8.6507450	-3.9673550
H	-2.5156700	7.7116040	-2.0854470
H	-3.0792680	9.4948990	-3.9217690
H	-0.9346340	10.2030440	-5.2841080
C	-0.7758280	-4.9408470	-0.2791530
C	-1.8762370	-5.6499070	-0.7032580
C	-1.5693260	-6.7079850	-1.5946470
C	-0.2256360	-6.8256230	-1.8721390
S	0.6802880	-5.5941180	-1.0123550
H	-2.8838780	-5.4109010	-0.3905680
H	-2.3185610	-7.3669050	-2.0145170
C	0.4491140	-7.7702910	-2.7345460
S	-0.4400450	-8.6507450	-3.9673550
C	0.9945470	-9.4822390	-4.4818270
C	2.0870420	-9.0949460	-3.7586410
C	1.7800660	-8.1224270	-2.7650690
H	0.9346340	-10.2030440	-5.2841080
H	3.0792680	-9.4948990	-3.9217690
H	2.5156700	-7.7116040	-2.0854470
H	0.8926590	-1.3117030	4.9425590
H	-0.8926590	1.3117030	4.9425590

(*R*)-Dinaphtho[2,1-*d*:1’,2’-*f*][1,3]dioxepine ((a*R*)-**4**):

Energy –959.473645 h, no imaginary frequencies

Symbol	X	Y	Z
H	1.5149350	3.0501050	2.5931860
C	1.0175310	2.6743650	1.7070500
H	0.9297200	4.5572000	0.6997450
C	0.6889130	3.5006580	0.6619580
C	0.1104250	0.7340150	0.5188120
C	0.0000000	2.9947170	-0.4702090
C	0.7099180	1.3021260	1.6299680
C	-0.3196590	1.5996680	-0.5386980
C	-0.4066570	3.8544650	-1.5247750
H	-1.3861710	0.1031780	-1.6864800
C	-1.1294860	3.3728910	-2.5891750
H	-0.1448710	4.9053600	-1.4638710
H	-1.4400750	4.0386550	-3.3862050
C	-1.4879230	2.0055990	-2.6351320
H	-2.0842580	1.6355240	-3.4614670
C	-1.0936200	1.1432010	-1.6393570
C	-0.7099180	-1.3021260	1.6299680
C	-0.1104250	-0.7340150	0.5188120
C	-0.6889130	-3.5006580	0.6619580
C	0.3196590	-1.5996680	-0.5386980
C	-1.0175310	-2.6743650	1.7070500
C	0.0000000	-2.9947170	-0.4702090
C	1.0936200	-1.1432010	-1.6393570

H	-1.5149350	-3.0501050	2.5931860
H	0.1448710	-4.9053600	-1.4638710
H	-0.9297200	-4.5572000	0.6997450
C	1.4879230	-2.0055990	-2.6351320
H	1.3861710	-0.1031780	-1.6864800
H	2.0842580	-1.6355240	-3.4614670
C	1.1294860	-3.3728910	-2.5891750
H	1.4400750	-4.0386550	-3.3862050
C	0.4066570	-3.8544650	-1.5247750
O	-1.0780540	-0.4861000	2.6850040
O	1.0780540	0.4861000	2.6850040
C	0.0000000	0.0000000	3.4722870
H	0.4315380	-0.7939500	4.0855470
H	-0.4315380	0.7939500	4.0855470

(*R*)-9,14-Dibromodinaphtho[2,1-*d*:1’,2’-*f*][1,3]dioxepine ((a*R*)-**5**):

Energy –6106.571877 h, no imaginary frequencies

Symbol	X	Y	Z
H	1.5082300	3.0554930	3.8144750
C	1.0123410	2.6771920	2.9286870
H	0.9259750	4.5587600	1.9178370
C	0.6858600	3.5023630	1.8824590
C	0.1090490	0.7338380	1.7438220
C	0.0000000	2.9908640	0.7510950
C	0.7063960	1.3045110	2.8551580
C	-0.3183610	1.5968260	0.6850760
C	-0.3989470	3.8544480	-0.3033520
H	-1.3888850	0.1009910	-0.4645940
C	-1.1134120	3.3496550	-1.3567610
H	-0.1384190	4.9039390	-0.2461190
C	-1.4836740	1.9897680	-1.4197140
H	-2.0770280	1.6206840	-2.2461530
C	-1.0896040	1.1387750	-0.4158400
C	-0.7063960	-1.3045110	2.8551580
C	-0.1090490	-0.7338380	1.7438220
C	-0.6858600	-3.5023630	1.8824590
C	0.3183610	-1.5968260	0.6850760
C	-1.0123410	-2.6771920	2.9286870
C	0.0000000	-2.9908640	0.7510950
C	1.0896040	-1.1387750	-0.4158400
H	-1.5082300	-3.0554930	3.8144750
H	0.1384190	-4.9039390	-0.2461190
H	-0.9259750	-4.5587600	1.9178370
C	1.4836740	-1.9897680	-1.4197140
H	1.3888850	-0.1009910	-0.4645940
H	2.0770280	-1.6206840	-2.2461530
C	1.1134120	-3.3496550	-1.3567610
C	0.3989470	-3.8544480	-0.3033520
O	-1.0756530	-0.4914710	3.9098020
O	1.0756530	0.4914710	3.9098020
Br	1.6570050	-4.5146380	-2.7874660
Br	-1.6570050	4.5146380	-2.7874660
C	0.0000000	0.0000000	4.6977670

H	-0.4354540	0.7919840	5.3103610
H	0.4354540	-0.7919840	5.3103610

(*R*)-9,14-Di([2,2′-bithiophen]-5-yl)dinaphtho[2,1-*d*:1′,2′-*f*][1,3]dioxepine ((a*R*)-**6**):

Conformer 1, Energy –3167.091759 h, no imaginary frequencies

Symbol	X	Y	Z
H	2.2314200	2.5756510	5.4187380
C	1.6561500	2.3340230	4.5330050
H	2.0442480	4.1771610	3.5226760
C	1.5462390	3.2148920	3.4862800
C	0.2914990	0.6822830	3.3469000
C	0.7532130	2.8943370	2.3539600
C	1.0143240	1.0831230	4.4593340
C	0.0978920	1.6236740	2.2889960
C	0.5856120	3.8224020	1.2982800
H	-1.3150960	0.4537630	1.1335630
C	-0.2307850	3.5544360	0.2143140
H	1.1253970	4.7608310	1.3653290
C	-0.9235010	2.3081550	0.1869450
H	-1.6068130	2.0970240	-0.6260630
C	-0.7605200	1.3809090	1.1819260
C	-1.0143240	-1.0831230	4.4593340
C	-0.2914990	-0.6822830	3.3469000
C	-1.5462390	-3.2148920	3.4862800
C	-0.0978920	-1.6236740	2.2889960
C	-1.6561500	-2.3340230	4.5330050
C	-0.7532130	-2.8943370	2.3539600
C	0.7605200	-1.3809090	1.1819260
H	-2.2314200	-2.5756510	5.4187380
H	-1.1253970	-4.7608310	1.3653290
H	-2.0442480	-4.1771610	3.5226760
C	0.9235010	-2.3081550	0.1869450
H	1.3150960	-0.4537630	1.1335630
H	1.6068130	-2.0970240	-0.6260630
C	0.2307850	-3.5544360	0.2143140
C	-0.5856120	-3.8224020	1.2982800
O	-1.1654560	-0.2019450	5.5142870
O	1.1654560	0.2019450	5.5142870
C	0.8953920	-4.2769870	-2.1466820
H	1.2006980	-3.2982650	-2.4913100
C	0.4116120	-4.5070540	-0.8788480
C	0.9221090	-5.4308450	-2.9697600
H	1.2494440	-5.4179070	-4.0015050
C	0.4626020	-6.5667170	-2.3422460
S	0.0000000	-6.2037070	-0.6895410
C	-0.8953920	4.2769870	-2.1466820
H	-1.2006980	3.2982650	-2.4913100
C	-0.4116120	4.5070540	-0.8788480
C	-0.9221090	5.4308450	-2.9697600
H	-1.2494440	5.4179070	-4.0015050
C	-0.4626020	6.5667170	-2.3422460
S	0.0000000	6.2037070	-0.6895410
C	0.0000000	0.0000000	6.3016620

H	0.2197580	-0.8765670	6.9147510
H	-0.2197580	0.8765670	6.9147510
C	0.4208560	8.9563620	-2.3901540
H	1.0785260	8.8681700	-1.5349310
C	-0.3453230	7.9120520	-2.8580460
C	0.2815190	10.1486870	-3.1551940
H	0.8123740	11.0668130	-2.9399560
C	-0.5867710	10.0126360	-4.2014160
H	-0.8734030	10.7446430	-4.9420290
S	-1.2659400	8.4164440	-4.2657150
C	-0.4208560	-8.9563620	-2.3901540
H	-1.0785260	-8.8681700	-1.5349310
C	0.3453230	-7.9120520	-2.8580460
C	-0.2815190	-10.1486870	-3.1551940
H	-0.8123740	-11.0668130	-2.9399560
C	0.5867710	-10.0126360	-4.2014160
H	0.8734030	-10.7446430	-4.9420290
S	1.2659400	-8.4164440	-4.2657150

Conformer 2, Energy –3167.091727 h, no imaginary frequencies

Symbol	X	Y	Z
H	2.0735090	2.7054240	5.6203420
C	1.5142660	2.4293590	4.7344020
H	1.7938850	4.2910220	3.7221460
C	1.3536360	3.3009470	3.6864480
C	0.2504760	0.6984360	3.5497030
C	0.5833580	2.9321800	2.5529480
C	0.9483390	1.1421350	4.6615760
C	0.0000000	1.6266260	2.4919520
C	0.3561280	3.8495390	1.4989630
H	-1.3331750	0.3694510	1.3335600
C	-0.4401860	3.5299500	0.4139440
H	0.8076860	4.8322410	1.5814210
C	-1.0556160	2.2442600	0.3864290
H	-1.6951760	1.9789770	-0.4460450
C	-0.8442050	1.3324630	1.3866540
C	-0.9483390	-1.1421350	4.6615760
C	-0.2504760	-0.6984360	3.5497030
C	-1.3536360	-3.3009470	3.6864480
C	0.0000000	-1.6266260	2.4919520
C	-1.5142660	-2.4293590	4.7344020
C	-0.5833580	-2.9321800	2.5529480
C	0.8442050	-1.3324630	1.3866540
H	-2.0735090	-2.7054240	5.6203420
H	-0.8076860	-4.8322410	1.5814210
H	-1.7938850	-4.2910220	3.7221460
C	1.0556160	-2.2442600	0.3864290
H	1.3331750	-0.3694510	1.3335600
H	1.6951760	-1.9789770	-0.4460450
C	0.4401860	-3.5299500	0.4139440
C	-0.3561280	-3.8495390	1.4989630
O	-1.1513710	-0.2723360	5.7171680
O	1.1513710	0.2723360	5.7171680
C	1.7032880	-4.4778760	-1.5980980

H	2.5055080	-3.7523690	-1.5907150
C	0.6768330	-4.4679460	-0.6814240
C	1.6331530	-5.5468430	-2.5264800
H	2.3752730	-5.7160200	-3.2961350
C	0.5455970	-6.3699620	-2.3392880
S	-0.4218420	-5.8019020	-0.9908190
C	-1.7032880	4.4778760	-1.5980980
H	-2.5055080	3.7523690	-1.5907150
C	-0.6768330	4.4679460	-0.6814240
C	-1.6331530	5.5468430	-2.5264800
H	-2.3752730	5.7160200	-3.2961350
C	-0.5455970	6.3699620	-2.3392880
S	0.4218420	5.8019020	-0.9908190
C	0.0000000	0.0000000	6.5041440
H	0.2724230	-0.8614580	7.1173620
H	-0.2724230	0.8614580	7.1173620
C	0.7043170	8.5584490	-2.7178010
H	1.1877770	8.6007810	-1.7503020
C	-0.1482220	7.5418270	-3.0866530
C	0.8653030	9.5551310	-3.7214110
H	1.4904930	10.4306630	-3.6047970
C	0.1379920	9.2986470	-4.8492990
H	0.0661200	9.8830590	-5.7546920
S	-0.7553000	7.8166460	-4.7115130
C	-0.7043170	-8.5584490	-2.7178010
H	-1.1877770	-8.6007810	-1.7503020
C	0.1482220	-7.5418270	-3.0866530
C	-0.8653030	-9.5551310	-3.7214110
H	-1.4904930	-10.4306630	-3.6047970
C	-0.1379920	-9.2986470	-4.8492990
H	-0.0661200	-9.8830590	-5.7546920
S	0.7553000	-7.8166460	-4.7115130

Conformer 3, Energy –3167.091593 h, no imaginary frequencies

Symbol	X	Y	Z
H	2.2574000	2.5543140	5.4597530
C	1.6799390	2.3176900	4.5740920
H	2.0895840	4.1546750	3.5610200
C	1.5805620	3.1981340	3.5259090
C	0.2981810	0.6793480	3.3894290
C	0.7850220	2.8846280	2.3933430
C	1.0249270	1.0735450	4.5016650
C	0.1148500	1.6216760	2.3305650
C	0.6304130	3.8117220	1.3347820
H	-1.3128130	0.4672160	1.1775320
C	-0.1872350	3.5507350	0.2499670
H	1.1815970	4.7436540	1.4010900
C	-0.8964880	2.3136560	0.2262660
H	-1.5813930	2.1082650	-0.5867820
C	-0.7463650	1.3872410	1.2239030
C	-1.0249270	-1.0735450	4.5016650
C	-0.2981810	-0.6793480	3.3894290
C	-1.5805620	-3.1981340	3.5259090
C	-0.1148500	-1.6216760	2.3305650

C	-1.6799390	-2.3176900	4.5740920
C	-0.7850220	-2.8846280	2.3933430
C	0.7463650	-1.3872410	1.2239030
H	-2.2574000	-2.5543140	5.4597530
H	-1.1815970	-4.7436540	1.4010900
H	-2.0895840	-4.1546750	3.5610200
C	0.8964880	-2.3136560	0.2262660
H	1.3128130	-0.4672160	1.1775320
H	1.5813930	-2.1082650	-0.5867820
C	0.1872350	-3.5507350	0.2499670
C	-0.6304130	-3.8117220	1.3347820
O	-1.1673230	-0.1914220	5.5570010
O	1.1673230	0.1914220	5.5570010
C	0.8569830	-4.2727780	-2.1092670
H	1.2045620	-3.3031290	-2.4388910
C	0.3493010	-4.4988030	-0.8501520
C	0.8705900	-5.4221040	-2.9385410
H	1.2444120	-5.4170570	-3.9544700
C	0.3721990	-6.5504270	-2.3270720
S	-0.1282280	-6.1812530	-0.6862810
C	-0.8569830	4.2727780	-2.1092670
H	-1.2045620	3.3031290	-2.4388910
C	-0.3493010	4.4988030	-0.8501520
C	-0.8705900	5.4221040	-2.9385410
H	-1.2444120	5.4170570	-3.9544700
C	-0.3721990	6.5504270	-2.3270720
S	0.1282280	6.1812530	-0.6862810
C	0.0000000	0.0000000	6.3441870
H	0.2118300	-0.8785330	6.9572680
H	-0.2118300	0.8785330	6.9572680
C	0.0000000	9.0688080	-2.1894090
H	0.0902870	9.1303400	-1.1124690
C	-0.2284840	7.8869110	-2.8585880
C	0.0852830	10.2001220	-3.0492210
H	0.2537880	11.2101010	-2.6991520
C	-0.0777000	9.8813700	-4.3677790
H	-0.0674120	10.5341130	-5.2283860
S	-0.3297900	8.1781100	-4.5877460
C	0.0000000	-9.0688080	-2.1894090
H	-0.0902870	-9.1303400	-1.1124690
C	0.2284840	-7.8869110	-2.8585880
C	-0.0852830	-10.2001220	-3.0492210
H	-0.2537880	-11.2101010	-2.6991520
C	0.0777000	-9.8813700	-4.3677790
H	0.0674120	-10.5341130	-5.2283860
S	0.3297900	-8.1781100	-4.5877460

Conformer 4, Energy –3167.091488 h, no imaginary frequencies

Symbol	X	Y	Z
H	2.1400570	2.6513970	5.5688360
C	1.5739170	2.3901820	4.6827770
H	1.8966890	4.2464680	3.6736020
C	1.4331700	3.2671370	3.6365290
C	0.2680080	0.6919490	3.4960540

C	0.6534470	2.9187300	2.5030680
C	0.9768080	1.1172820	4.6082680
C	0.0400130	1.6271730	2.4394470
C	0.4460400	3.8440380	1.4521440
H	-1.3204730	0.4035060	1.2768400
C	-0.3579090	3.5464530	0.3664080
H	0.9212610	4.8152660	1.5381600
C	-1.0008950	2.2742680	0.3352430
H	-1.6464230	2.0243710	-0.4972740
C	-0.8098880	1.3550860	1.3328680
C	-0.9768080	-1.1172820	4.6082680
C	-0.2680080	-0.6919490	3.4960540
C	-1.4331700	-3.2671370	3.6365290
C	-0.0400130	-1.6271730	2.4394470
C	-1.5739170	-2.3901820	4.6827770
C	-0.6534470	-2.9187300	2.5030680
C	0.8098880	-1.3550860	1.3328680
H	-2.1400570	-2.6513970	5.5688360
H	-0.9212610	-4.8152660	1.5381600
H	-1.8966890	-4.2464680	3.6736020
C	1.0008950	-2.2742680	0.3352430
H	1.3204730	-0.4035060	1.2768400
H	1.6464230	-2.0243710	-0.4972740
C	0.3579090	-3.5464530	0.3664080
C	-0.4460400	-3.8440380	1.4521440
O	-1.1579490	-0.2418740	5.6633050
O	1.1579490	0.2418740	5.6633050
C	1.5852350	-4.5117020	-1.6598340
H	2.3804970	-3.7790330	-1.6820210
C	0.5777480	-4.4961710	-0.7225040
C	1.4939060	-5.5828730	-2.5836260
H	2.2026560	-5.7353250	-3.3874980
C	0.4113150	-6.4062090	-2.3699160
S	-0.5151340	-5.8447080	-0.9896820
C	-1.5852350	4.5117020	-1.6598340
H	-2.3804970	3.7790330	-1.6820210
C	-0.5777480	4.4961710	-0.7225040
C	-1.4939060	5.5828730	-2.5836260
H	-2.2026560	5.7353250	-3.3874980
C	-0.4113150	6.4062090	-2.3699160
S	0.5151340	5.8447080	-0.9896820
C	0.0000000	0.0000000	6.4506120
H	0.2496700	-0.8683900	7.0637970
H	-0.2496700	0.8683900	7.0637970
C	1.2097270	8.2406950	-3.0762080
H	2.0460080	7.9152300	-2.4707270
C	0.0000000	7.5828640	-3.1018890
C	1.2574120	9.3744270	-3.9355040
H	2.1279180	10.0072470	-4.0484090
C	0.0889140	9.5792490	-4.6132150
H	-0.1519130	10.3522530	-5.3279950
S	-1.1055350	8.3877320	-4.2044580
C	-1.2097270	-8.2406950	-3.0762080
H	-2.0460080	-7.9152300	-2.4707270
C	0.0000000	-7.5828640	-3.1018890
C	-1.2574120	-9.3744270	-3.9355040

H	-2.1279180	-10.0072470	-4.0484090
C	-0.0889140	-9.5792490	-4.6132150
H	0.1519130	-10.3522530	-5.3279950
S	1.1055350	-8.3877320	-4.2044580

Conformer 5, Energy –3167.090853 h, no imaginary frequencies

Symbol	X	Y	Z
H	2.4701050	2.3517910	6.0383710
C	1.8727550	2.1664740	5.1536550
H	2.4385110	3.9607600	4.1396050
C	1.8484380	3.0519120	4.1059080
C	0.3542840	0.6518200	3.9712230
C	1.0239910	2.8102560	2.9753980
C	1.1123140	0.9829210	5.0823470
C	0.2484860	1.6081010	2.9132080
C	0.9456630	3.7502480	1.9209510
H	-1.2704580	0.5770340	1.7596820
C	0.0977180	3.5637060	0.8430660
H	1.5735740	4.6318300	1.9769520
C	-0.7070720	2.3889680	0.8160430
H	-1.4139920	2.2414470	0.0076480
C	-0.6306550	1.4473170	1.8092430
C	-1.1123140	-0.9829210	5.0823470
C	-0.3542840	-0.6518200	3.9712230
C	-1.8484380	-3.0519120	4.1059080
C	-0.2484860	-1.6081010	2.9132080
C	-1.8727550	-2.1664740	5.1536550
C	-1.0239910	-2.8102560	2.9753980
C	0.6306550	-1.4473170	1.8092430
H	-2.4701050	-2.3517910	6.0383710
H	-1.5735740	-4.6318300	1.9769520
H	-2.4385110	-3.9607600	4.1396050
C	0.7070720	-2.3889680	0.8160430
H	1.2704580	-0.5770340	1.7596820
H	1.4139920	-2.2414470	0.0076480
C	-0.0977180	-3.5637060	0.8430660
C	-0.9456630	-3.7502480	1.9209510
O	-1.1794980	-0.0926130	6.1381930
O	1.1794980	0.0926130	6.1381930
C	-0.3479240	-5.8942560	-0.1879000
H	-0.7007110	-6.3857190	0.7090310
C	-0.0168230	-4.5590500	-0.2252150
C	-0.1454010	-6.5700180	-1.4169450
H	-0.3284150	-7.6285640	-1.5507590
C	0.3445660	-5.7608010	-2.4180160
S	0.5436770	-4.1213380	-1.8302110
C	0.3479240	5.8942560	-0.1879000
H	0.7007110	6.3857190	0.7090310
C	0.0168230	4.5590500	-0.2252150
C	0.1454010	6.5700180	-1.4169450
H	0.3284150	7.6285640	-1.5507590
C	-0.3445660	5.7608010	-2.4180160
S	-0.5436770	4.1213380	-1.8302110
C	0.0000000	0.0000000	6.9250710

H	0.1370340	-0.8931700	7.5382700
H	-0.1370340	0.8931700	7.5382700
C	-1.5009920	5.4300660	-4.6650540
H	-2.0376460	4.5271830	-4.4030860
C	-0.6793030	6.0970750	-3.7839470
C	-1.5956690	6.0591290	-5.9386480
H	-2.2015850	5.6819360	-6.7520670
C	-0.8498590	7.2005580	-6.0267250
H	-0.7436170	7.8759950	-6.8628530
S	0.0000000	7.5247890	-4.5481080
C	1.5009920	-5.4300660	-4.6650540
H	2.0376460	-4.5271830	-4.4030860
C	0.6793030	-6.0970750	-3.7839470
C	1.5956690	-6.0591290	-5.9386480
H	2.2015850	-5.6819360	-6.7520670
C	0.8498590	-7.2005580	-6.0267250
H	0.7436170	-7.8759950	-6.8628530
S	0.0000000	-7.5247890	-4.5481080

Conformer 6, Energy –3167.090791 h, no imaginary frequencies

Symbol	X	Y	Z
H	2.7138230	2.0619780	5.8279600
C	2.1019180	1.9436690	4.9417560
H	2.8638130	3.6652060	3.9298540
C	2.1777340	2.8264740	3.8942880
C	0.4260570	0.6073790	3.7569700
C	1.3385940	2.6739730	2.7589890
C	1.2157380	0.8511300	4.8685540
C	0.4299530	1.5687680	2.6983420
C	1.3626920	3.6201860	1.7075980
H	-1.1911050	0.7118770	1.5415850
C	0.5118870	3.5196010	0.6203280
H	2.0530810	4.4518430	1.7859030
C	-0.4222740	2.4451760	0.5945330
H	-1.1116210	2.3527500	-0.2369000
C	-0.4624090	1.5089440	1.5951010
C	-1.2157380	-0.8511300	4.8685540
C	-0.4260570	-0.6073790	3.7569700
C	-2.1777340	-2.8264740	3.8942880
C	-0.4299530	-1.5687680	2.6983420
C	-2.1019180	-1.9436690	4.9417560
C	-1.3385940	-2.6739730	2.7589890
C	0.4624090	-1.5089440	1.5951010
H	-2.7138230	-2.0619780	5.8279600
H	-2.0530810	-4.4518430	1.7859030
H	-2.8638130	-3.6652060	3.9298540
C	0.4222740	-2.4451760	0.5945330
H	1.1911050	-0.7118770	1.5415850
H	1.1116210	-2.3527500	-0.2369000
C	-0.5118870	-3.5196010	0.6203280
C	-1.3626920	-3.6201860	1.7075980
O	-1.1822700	0.0415030	5.9241500
O	1.1822700	-0.0415030	5.9241500
C	-1.6133950	-5.3111330	-0.8376170

H	-2.5796290	-5.2808760	-0.3518250
C	-0.5665120	-4.5006840	-0.4626550
C	-1.3201810	-6.1549910	-1.9377680
H	-2.0409900	-6.8331810	-2.3763390
C	-0.0402590	-6.0042370	-2.4235030
S	0.8262030	-4.8008290	-1.4884440
C	1.6133950	5.3111330	-0.8376170
H	2.5796290	5.2808760	-0.3518250
C	0.5665120	4.5006840	-0.4626550
C	1.3201810	6.1549910	-1.9377680
H	2.0409900	6.8331810	-2.3763390
C	0.0402590	6.0042370	-2.4235030
S	-0.8262030	4.8008290	-1.4884440
C	0.0000000	0.0000000	6.7113490
H	0.0351690	-0.9028830	7.3245900
H	-0.0351690	0.9028830	7.3245900
C	-1.7111010	6.3035680	-4.2517130
H	-2.2430790	5.3772290	-4.0761500
C	-0.6051920	6.6839710	-3.5245990
C	-2.0671820	7.2332000	-5.2694970
H	-2.9023330	7.0960410	-5.9438210
C	-1.2361830	8.3167170	-5.3170110
H	-1.2664260	9.1630630	-5.9875360
S	0.0000000	8.2283270	-4.1013770
C	1.7111010	-6.3035680	-4.2517130
H	2.2430790	-5.3772290	-4.0761500
C	0.6051920	-6.6839710	-3.5245990
C	2.0671820	-7.2332000	-5.2694970
H	2.9023330	-7.0960410	-5.9438210
C	1.2361830	-8.3167170	-5.3170110
H	1.2664260	-9.1630630	-5.9875360
S	0.0000000	-8.2283270	-4.1013770

Conformer 7, Energy –3167.090706 h, no imaginary frequencies

Symbol	X	Y	Z
H	-2.4619940	2.3587210	6.0226570
C	-1.8654700	2.1720700	5.1376700
H	-2.4242350	3.9695890	4.1254120
C	-1.8377510	3.0584580	4.0908590
C	-0.3524040	0.6529270	3.9537750
C	-1.0143630	2.8146220	2.9601150
C	-1.1093130	0.9858730	5.0651800
C	-0.2436410	1.6095140	2.8963050
C	-0.9325770	3.7555330	1.9067490
H	1.2696580	0.5730450	1.7401490
C	-0.0863700	3.5666350	0.8280790
H	-1.5566470	4.6397010	1.9643280
C	0.7130460	2.3883170	0.7988040
H	1.4181650	2.2379780	-0.0106850
C	0.6337250	1.4460630	1.7912560
C	1.1093130	-0.9858730	5.0651800
C	0.3524040	-0.6529270	3.9537750
C	1.8377510	-3.0584580	4.0908590
C	0.2436410	-1.6095140	2.8963050

C	1.8654700	-2.1720700	5.1376700
C	1.0143630	-2.8146220	2.9601150
C	-0.6337250	-1.4460630	1.7912560
H	2.4619940	-2.3587210	6.0226570
H	1.5566470	-4.6397010	1.9643280
H	2.4242350	-3.9695890	4.1254120
C	-0.7130460	-2.3883170	0.7988040
H	-1.2696580	-0.5730450	1.7401490
H	-1.4181650	-2.2379780	-0.0106850
C	0.0863700	-3.5666350	0.8280790
C	0.9325770	-3.7555330	1.9067490
O	1.1791140	-0.0953450	6.1206120
O	-1.1791140	0.0953450	6.1206120
C	0.3279720	-5.9004390	-0.1964320
H	0.6834990	-6.3896060	0.7006300
C	0.0000000	-4.5647600	-0.2374410
C	0.1424770	-6.5747840	-1.4288650
H	0.3602340	-7.6258670	-1.5697520
C	-0.3346490	-5.7651690	-2.4358300
S	-0.5636700	-4.1309390	-1.8427460
C	-0.3279720	5.9004390	-0.1964320
H	-0.6834990	6.3896060	0.7006300
C	0.0000000	4.5647600	-0.2374410
C	-0.1424770	6.5747840	-1.4288650
H	-0.3602340	7.6258670	-1.5697520
C	0.3346490	5.7651690	-2.4358300
S	0.5636700	4.1309390	-1.8427460
C	0.0000000	0.0000000	6.9077090
H	0.1391090	0.8928640	7.5209020
H	-0.1391090	-0.8928640	7.5209020
C	-0.8143330	-5.2677220	-4.8894440
H	-0.7030610	-4.1927290	-4.8286620
C	-0.6486800	-6.1021100	-3.8065650
C	-1.1213600	-5.9582220	-6.0959680
H	-1.2776200	-5.4662910	-7.0470990
C	-1.1888980	-7.3129920	-5.9327890
H	-1.3941920	-8.0739520	-6.6712080
S	-0.8883070	-7.7745300	-4.2864700
C	0.8143330	5.2677220	-4.8894440
H	0.7030610	4.1927290	-4.8286620
C	0.6486800	6.1021100	-3.8065650
C	1.1213600	5.9582220	-6.0959680
H	1.2776200	5.4662910	-7.0470990
C	1.1888980	7.3129920	-5.9327890
H	1.3941920	8.0739520	-6.6712080
S	0.8883070	7.7745300	-4.2864700

Conformer 8, Energy –3167.090648 h, no imaginary frequencies

Symbol	X	Y	Z
H	2.6900050	2.0927710	5.8825550
C	2.0801150	1.9671840	4.9959600
H	2.8250880	3.6952510	3.9825580
C	2.1476540	2.8495010	3.9475220
C	0.4193510	0.6119680	3.8111640

C	1.3117070	2.6864610	2.8113600
C	1.2059010	0.8650740	4.9230020
C	0.4142860	1.5721610	2.7514880
C	1.3293880	3.6300360	1.7575210
H	-1.1983200	0.6991460	1.5948520
C	0.4829330	3.5184150	0.6678710
H	2.0127640	4.4673720	1.8360140
C	-0.4420570	2.4358630	0.6441820
H	-1.1306680	2.3350860	-0.1869150
C	-0.4761650	1.5022130	1.6472620
C	-1.2059010	-0.8650740	4.9230020
C	-0.4193510	-0.6119680	3.8111640
C	-2.1476540	-2.8495010	3.9475220
C	-0.4142860	-1.5721610	2.7514880
C	-2.0801150	-1.9671840	4.9959600
C	-1.3117070	-2.6864610	2.8113600
C	0.4761650	-1.5022130	1.6472620
H	-2.6900050	-2.0927710	5.8825550
H	-2.0127640	-4.4673720	1.8360140
H	-2.8250880	-3.6952510	3.9825580
C	0.4420570	-2.4358630	0.6441820
H	1.1983200	-0.6991460	1.5948520
H	1.1306680	-2.3350860	-0.1869150
C	-0.4829330	-3.5184150	0.6678710
C	-1.3293880	-3.6300360	1.7575210
O	-1.1825620	0.0275210	5.9788340
O	1.1825620	-0.0275210	5.9788340
C	-1.5640750	-5.3338960	-0.7763610
H	-2.5170400	-5.3420520	-0.2643730
C	-0.5342990	-4.4931000	-0.4211860
C	-1.2655340	-6.1740060	-1.8777080
H	-1.9628150	-6.8991810	-2.2775990
C	0.0000000	-5.9873150	-2.3882950
S	0.8390910	-4.7384910	-1.4876780
C	1.5640750	5.3338960	-0.7763610
H	2.5170400	5.3420520	-0.2643730
C	0.5342990	4.4931000	-0.4211860
C	1.2655340	6.1740060	-1.8777080
H	1.9628150	6.8991810	-2.2775990
C	0.0000000	5.9873150	-2.3882950
S	-0.8390910	4.7384910	-1.4876780
C	0.0000000	0.0000000	6.7660510
H	0.0460070	-0.9024020	7.3793100
H	-0.0460070	0.9024020	7.3793100
C	-1.9825000	6.6997770	-3.8225130
H	-2.7512560	6.2224950	-3.2283420
C	-0.6447820	6.6519050	-3.4987970
C	-2.2550040	7.4549610	-4.9980760
H	-3.2488630	7.6059280	-5.3986720
C	-1.1297000	7.9810450	-5.5668460
H	-1.0430050	8.5949110	-6.4512550
S	0.2977070	7.5505220	-4.6774630
C	1.9825000	-6.6997770	-3.8225130
H	2.7512560	-6.2224950	-3.2283420
C	0.6447820	-6.6519050	-3.4987970
C	2.2550040	-7.4549610	-4.9980760

H	3.2488630	-7.6059280	-5.3986720
C	1.1297000	-7.9810450	-5.5668460
H	1.0430050	-8.5949110	-6.4512550
S	-0.2977070	-7.5505220	-4.6774630

Supplementary References

[S1] B. A. Jones, T. Balan, J. D. Jolliffe, C. D. Campbell, M. D. Smith, *Angew. Chem. Int. Ed.* **2019**, *58*, 4596-4600.

[S2] S. Kang, I. Cha, J. G. Han, C. Song, *Mater. Express* **2013**, *3*, 119-126.

[S3] Y. Zhang, H. Li, Z. Geng, W. Zheng, Y. Quan, Y. Cheng, *Nat. Commun.* **2022**, *13*, 4905.

[S4] H. J. Deussen, E. Hendrickx, C. Boutton, D. Krog, K. Clays, K. Bechgaard, A. Persoons, T. Bjørnholm, *J. Am. Chem. Soc.* **1996**, *118*, 6841-6852.

[S5] M. Li, P. Lincoln, J. Andersson, *J. Phys. Chem. B* **2011**, *115*, 7923-7931.

[S6] F. Zinna, T. Bruhn, C. A. Guido, J. Ahrens, M. Bröring, L. Di Bari, G. Pescitelli, *Chem. Eur. J.* **2016**, *22*, 16089-16098.

[S7] M. J. Frisch, G. W. Trucks, H. B. Schlegel, G. E. Scuseria, M. A. Robb, J. R. Cheeseman, G. Scalmani, V. Barone, G. A. Petersson, H. Nakatsuji, X. Li, M. Caricato, A. V. Marenich, J. Bloino, B. G. Janesko, R. Gomperts, B. Mennucci, H. P. Hratchian, J. V. Ortiz, A. F. Izmaylov, J. L. Sonnenberg, D. Williams-Young, F. Ding, F. Lipparini, F. Egidi, J. Goings, B. Peng, A. Petrone, T. Henderson, D. Ranasinghe, V. G. Zakrzewski, J. Gao, N. Rega, G. Zheng, W. Liang, M. Hada, M. Ehara, K. Toyota, R. Fukuda, J. Hasegawa, M. Ishida, T. Nakajima, Y. Honda, O. Kitao, H. Nakai, T. Vreven, K. Throssell, J. A. Montgomery Jr., J. E. Peralta, F. Ogliaro, M. J. Bearpark, J. J. Heyd, E. N. Brothers, K. N. Kudin, V. N. Staroverov, T. A. Keith, R. Kobayashi, J. Normand, K. Raghavachari, A. P. Rendell, J. C. Burant, S. S. Iyengar, J. Tomasi, M. Cossi, J. M. Millam, M. Klene, C. Adamo, R. Cammi, J. W. Ochterski, R. L. Martin, K. Morokuma, O. Farkas, J. B. Foresman, D. J. Fox in *Gaussian 16 Rev. B.01*, *Vol.* Wallingford, CT, **2016**.

[S8] a) T. Lu, F. Chen, *J. Comput. Chem.* **2012**, *33*, 580-592; b) T. Lu, *J. Chem. Phys.* **2024**, *161*, 082503.
